# Mesenchymal Stromal Cells Overexpressing Farnesoid X Receptor Exert Cardioprotective Effects Against Acute Ischemic Heart Injury by Binding Endogenous Bile Acids

**DOI:** 10.1002/advs.202200431

**Published:** 2022-07-03

**Authors:** Yunlong Xia, Xinyue Xu, Yongzhen Guo, Chen Lin, Xiaoming Xu, Fuyang Zhang, Miaomiao Fan, Tingting Qi, Congye Li, Guangyu Hu, Lu Peng, Shan Wang, Ling Zhang, Chunxu Hai, Rui Liu, Wenjun Yan, Ling Tao

**Affiliations:** ^1^ Cardiology Xijing Hospital Fourth Military Medical University Xi'an 710032 China; ^2^ State Key Laboratory of Military Stomatology National Clinical Research Center for Oral Diseases and Shaanxi Engineering Research Center for Dental Materials and Advanced Manufacture Department of Periodontology School of Stomatology Fourth Military Medical University Xi'an Shaanxi 710032 China; ^3^ Cardiology General Hospital of Eastern Theater Command of Chinese PLA Nanjing 210002 China; ^4^ Department of Toxicology Shanxi Provincial Key Lab of Free Radical Biology and Medicine Ministry of Education Key Lab of Hazard Assessment and Control in Special Operational Environment School of Public Health Fourth Military Medical University Xi'an Shaanxi 710032 P. R. China

**Keywords:** bile acids, farnesoid X receptor, ischemic heart injury, mesenchymal stromal cells, paracrine angiogenesis

## Abstract

Bile acid metabolites have been increasingly recognized as pleiotropic signaling molecules that regulate cardiovascular functions, but their role in mesenchymal stromal cells (MSC)‐based therapy has never been investigated. It is found that overexpression of farnesoid X receptor (FXR), a main receptor for bile acids, improves the retention and cardioprotection of adipose tissue‐derived MSC (ADSC) administered by intramyocardial injection in mice with myocardial infarction (MI), which shows enhanced antiapoptotic, proangiogenic, and antifibrotic effects. RNA sequencing, LC‐MS/MS, and loss‐of‐function studies reveal that FXR overexpression promotes ADSC paracrine angiogenesis via Angptl4. FXR overexpression improves ADSC survival in vivo but fails in vitro. By performing bile acid‐targeted metabolomics using ischemic heart tissue, 19 bile acids are identified. Among them, cholic acid and deoxycholic acid significantly increase Angptl4 secretion from ADSC overexpressing FXR and further improve their proangiogenic capability. Moreover, ADSC overexpressing FXR shows significantly lower apoptosis by upregulating Nqo‐1 expression only in the presence of FXR ligands. Retinoid X receptor *α* is identified as a coactivator of FXR. It is first demonstrated that there is a bile acid pool in the myocardial microenvironment. Targeting the bile acid‐FXR axis may be a novel strategy for improving the curative effect of MSC‐based therapy for MI.

## Graphical abstract

Adipose tissue‐derived MSC (ADSC) overexpressing farnesoid X receptor (FXR) are intramyocardially injected into the peri‐infarct area after myocardial infarction (MI) and respond to bile acids present in the myocardial microenvironment. Bile acid‐FXR axis activation improves the cardioprotection of ADSC via Angptl4‐mediated paracrine angiogenesis and Nqo‐1‐mediated survival. FXR activaton directly upregulates Angptl4 and Nqo‐1 expression by forming a heterodimer with RXRα.

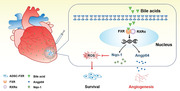

## 1 Introduction

Heart failure (HF) remains a major problem threatening human health, accounting for the high mortality and the rate of disability.^[^ ^1^, ^2^ ^]^ Myocardial infarction (MI) is a common cause of HF.^[^ ^3^ ^]^ Therefore, identifying novel therapeutic strategies that prevent the development of post‐MI HF is urgently needed.

In the past 20 years, the efficacy of stem cell therapy for cardiac repair after ischemic injury has been extensively studied.^[^ ^4^, ^5^ ^]^ Compared with other types of stem cells, mesenchymal stromal cells (MSC) have been more widely used in various basic and clinical studies because of several advantages, such as easy acquisition, low immunogenicity, rapid proliferation, and no ethical concerns.^[^ ^6^, ^7^ ^]^ Although it was once thought that MSC could differentiate into new cardiomyocytes, there is no evidence that new cardiomyocyte regeneration occurs after MSC transplantation. Increasing evidence shows that the paracrine mechanism mediated by cytokines or extracellular vesicles plays a vital role in myocardial repair mediated by MSC.^[^ ^8^, ^9^, ^10^ ^]^ Due to the harsh myocardial ischemia microenvironment, which is characterized by oxygen deficiency, limited nutrients, and increased reactive oxygen species (ROS) levels, it is difficult for MSC to survive and function sufficiently after transplantation.^[^ ^11^, ^12^ ^]^ Thus, the long‐term clinical efficacy of MSC in the treatment of MI is not satisfactory.^[^ ^6^, ^13^ ^]^ Therefore, improving the survival/retention rate of transplanted MSC and enhancing their paracrine cardioprotective effects are important strategies for improving the clinical therapeutic effects of MSC.

Farnesoid X receptor (FXR, Nr1h4), a nuclear receptor, is an important receptor for bile acids.^[^ ^14^ ^]^ Our previous study demonstrated that long‐term systemic FXR activation increases adipose tissue secretion of adiponectin, which circulates in plasma and improves cardiac function after MI.^[^ ^15^ ^]^ Several bile acids, which are the natural ligands for FXR, have been found to be involved in the process of metabolic and cardiovascular diseases.^[^ ^16^, ^17^ ^]^ However, the role of bile acids/FXR in MSC‐based therapy for post‐MI cardiac repair has never been investigated. In this study, we investigated whether FXR overexpression may enhance the cardioprotective effects of adipose tissue‐derived MSC (ADSC) against ischemic heart injury and elucidated the molecular mechanisms involved.

## 2 Results

### 2.1 Intramyocardial Injection of ADSC Overexpressing FXR Protects the Heart Against Post‐MI Cardiac Dysfunction and Remodeling

**Figure 1:**
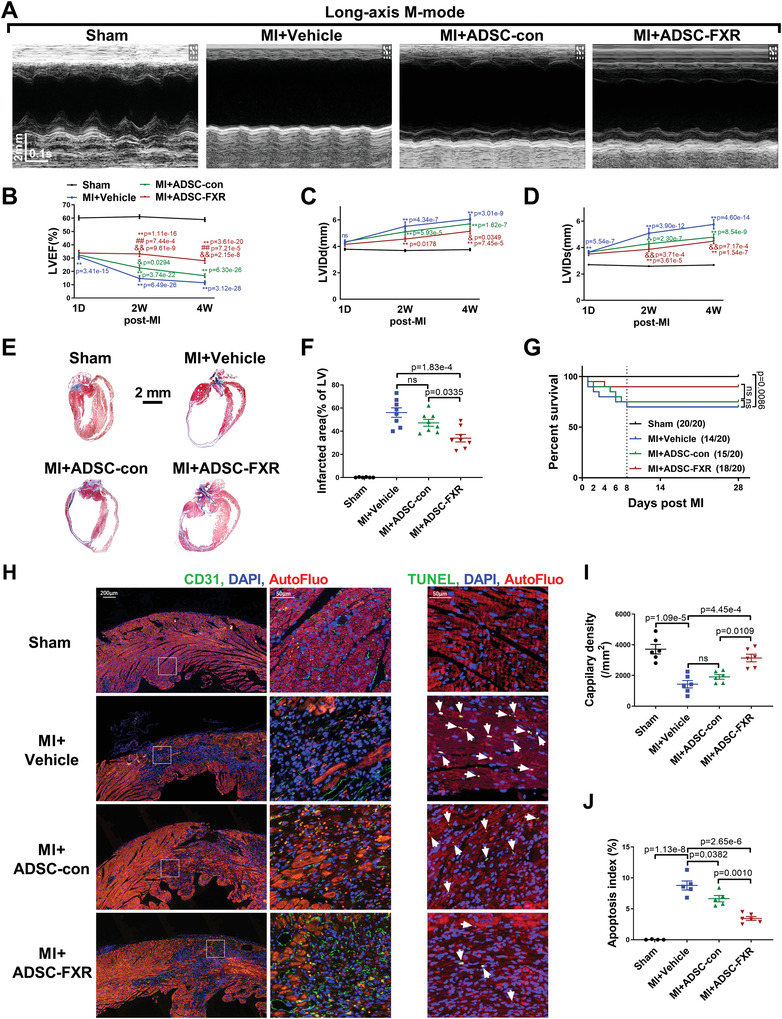
Intramyocardial injection of ADSC overexpressing FXR protected heart against post‐MI cardiac dysfunction and remodeling. A) Cardiac function was evaluated by long‐axis M‐mode echocardiography 4 weeks after MI, and representative images are shown. B–D) LVEF, LVIDd, and LVIDs were evaluated by long‐axis M‐mode echocardiography. A–D) *n* = 20, 18, 19, 19 at 1 D; *n* = 20, 14, 15, 18 at 2 and 4 W. *p and **p versus Sham, ^&^ *p* and ^&&^ *p* versus MI+vehicle, ^#^ *p* and ^##^ *p* versus MI+ADSC‐con. 1D means 1 day after MI surgery. 2 W means 2 weeks after MI surgery. 4 W means 4 weeks after MI surgery. E) The myocardial infarct area 4 weeks after MI was determined by Masson's trichrome staining. Representative images were shown. F) Quantification of the fibrotic area 4 weeks after MI (*n* = 6, 8, 8, 8). G) Survival of each group of mice. Numbers represent total animals studied (right) and surviving (left). H) Representative images of capillary density determined by CD31 immunofluorescence staining (left, green) and TUNEL‐positive cardiomyocytes (right, green, indicated by the white arrows) in the peri‐infarct area 7 days after MI. The nuclei were stained by DAPI (blue). I) Quantification of capillary density in the peri‐infarct area 7 days after MI (*n* = 6 per group). J) Quantification of cardiomyocyte apoptosis (the percentage of TUNEL‐positive nuclei/DAPI‐positive nuclei) in the peri‐infarct area 7 days after MI (*n* = 4, 5, 5, 6). Abbreviations: LVEF: left ventricular ejection fraction. LVIDd: left ventricular internal diameter at the end of diastole. LVIDs: left ventricular internal diameter at the end of systole. ADSC‐con: ADSC transfected with adenovirus control for 2 days. ADSC‐FXR: ADSC transfected with adenovirus‐FXR for 2 days. Data in G were analyzed using the Gehan–Breslow–Wilcoxon test. Other data were analyzed using one‐way ANOVA, followed by Bonferroni post hoc tests. Data are presented as the mean ± SEM. *p < 0.05, **p < 0.01. ns: not significant.

The ADSC used in this study were all derived from adult mouse adipose tissue and were identified by surface marker expression and differentiation potential as we previously described.^[^ ^18^ ^]^ ADSC were transfected with control adenovirus (ADSC‐con) or adenovirus harboring FXR (ADSC‐FXR). To investigate whether FXR overexpression provided benefits in terms of post‐MI cardiac injury, the mice were randomly divided into the following groups: sham MI, MI+vehicle, MI+ADSC‐con, or MI+ADSC‐FXR. The MI model was established by ligation of the left anterior descending coronary artery, and sham MI animals underwent the same procedure without coronary artery ligation. ADSC were intramyocardially injected immediately after MI. Echocardiography showed similar cardiac function in each group 24 h after MI (**Figure** **1**A–D; and Figure S1A–D (Supporting Information), time point 1 day). Compared to MI+vehicle treatment, intramyocardial delivery of ADSC‐con slightly improved cardiac function (time points 2 and 4 weeks). Compared to ADSC‐con, ADSC‐FXR significantly increased the left ventricular ejection fraction (LVEF) and decreased the left ventricular internal diameter at the end of systole (LVIDs) and diastole (LVIDd) after MI surgery (time points 2 and 4 weeks, Figure 1A–D; and Figure S1A–D, Supporting Information). Masson's trichome staining showed that ADSC‐con did not reduce the myocardial fibrotic area (*p* > 0.05 vs MI+vehicle, Figure 1E,F). However, ADSC‐FXR significantly reduced myocardial infarct size compared to the MI+vehicle or MI+ADSC‐con groups (*p* < 0.01 or *p* < 0.05; Figure 1E,F). More importantly, MI+ADSC‐FXR mice showed a higher survival rate than MI+ADSC‐con‐treated mice, although the difference was not statistically significant (90% vs 75%) (Figure 1G).

To clarify the cellular mechanism(s) responsible for FXR‐enhanced ADSC cardioprotection, angiogenesis and cardiomyocyte apoptosis were assessed. CD31 immunofluorescence staining (infarct border zone 7 days after MI; Figure 1H,I) demonstrated that the capillary density was significantly decreased in the MI+vehicle group compared to the sham group, and intramyocardial delivery of ADSC‐con did not increase the capillary density. Importantly, ADSC‐FXR conspicuously increased capillary density compared to that in the MI+vehicle or MI+ADSC‐con groups (both *p* < 0.01). At 7 days after MI, TUNEL staining was performed to evaluate cardiomyocyte apoptosis in the peri‐infarcted area. Compared to sham treatment, MI markedly increased the number of TUNEL‐positive cells (*p* < 0.01, Figure 1H,J). Notably, the MI+ADSC‐FXR group exhibited significantly fewer TUNEL‐positive cells in the peri‐infarcted areas than both the MI+vehicle and MI+ADSC‐con groups (both *p* < 0.01, Figure 1H,J).

### 2.2 FXR Overexpression Increases the ADSC Retention Rate In Vivo

The above results demonstrated that FXR overexpression enhances the cardioprotective capability of ADSC against ischemic heart disease (IHD). To explore whether FXR affects ADSC survival in ischemic heart tissue, we detected the ADSC retention rate at 3 and 7 days after intramyocardial injection. ADSC were isolated from FXR knockout mice (ADSC‐FXR^KO^) or wild‐type control mice (ADSC‐con), and were labeled with the lipophilic red fluorescent dye CM‐DiI. The mice were randomized to receive one of the following treatments by intramyocardial injection: MI+ADSC‐con, MI+ADSC‐FXR, or MI+ADSC‐FXR^KO^. The ADSC retention rate was determined by quantifying CM‐DiI‐positive cells in heart sections. As shown in Figure S2 (Supporting Information), CM‐DiI‐positive cells were visualized in the peri‐infarcted (troponin T negative) areas of heart sections. Significantly more CM‐DiI‐positive cells were present in the heart sections from the MI+ADSC‐FXR group than in those from the MI+ADSC‐con group (both *p* < 0.01, time points MI 3 and 7 days; Figure S2, Supporting Information). However, the number of CM‐DiI‐positive cells in the MI+ADSC‐FXR^KO^ group was not significantly different from that in the MI+ADSC‐con group 3 and 7 days after transplantation (*p* > 0.05, Figure S2, Supporting Information). Apoptosis is thought to be a main reason for MSC loss after transplantation.^[^ ^11^, ^19^, ^20^ ^]^ We performed TUNEL staining and revealed that FXR overexpression significantly reduced TUNEL‐positive ADSC 3 days and 7 days after intramyocardial injection (Figure S3, Supporting Information).

**Figure 2:**
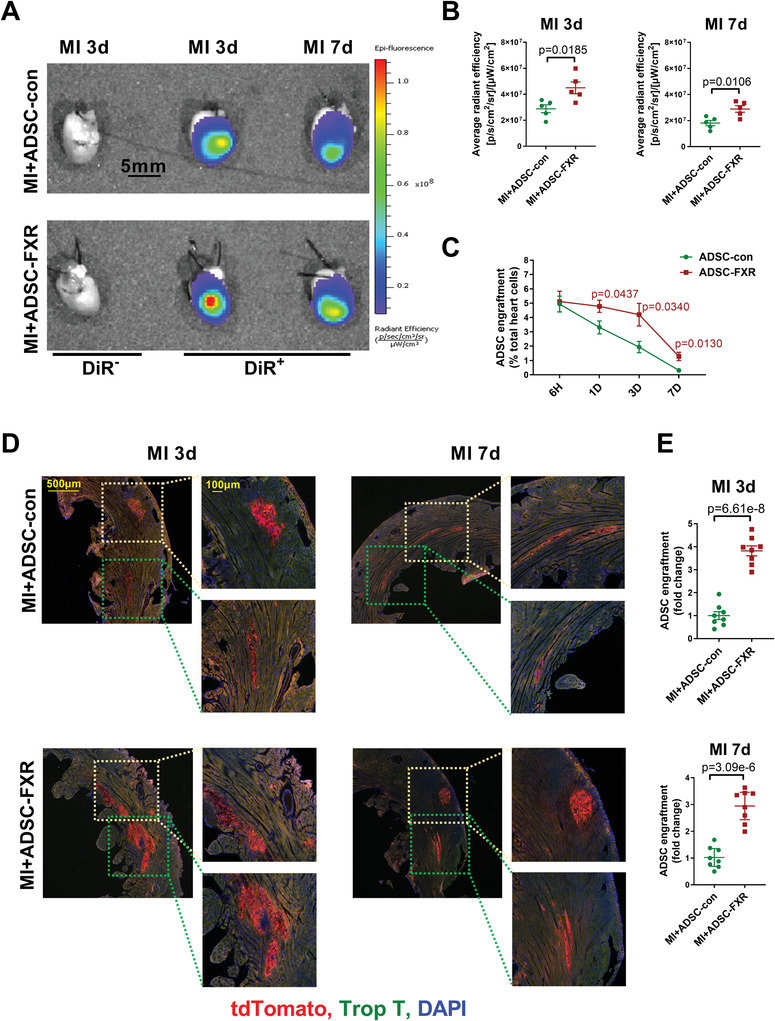
FXR overexpression increased the ADSC retention rate in vivo. A) Representative images of fluorescence intensity at 3 and 7 days after injection with DiR‐labeled ADSC. B) Quantification of DiR‐labeled ADSC was determined by the average radiant efficiency (left, MI3d; right, MI7d; *n* = 5 per group). C) ADSC engraftment was quantified as the number of tdTomato‐positve cells per 100 heart cells in apex region (*n* = 4 mice). D) Representative images of tdToamto‐ADSC in hearts 3 and 7 days after MI. Heart tissue was immunostained for troponin T (green) and DAPI (blue). E) Quantification of tdTomato‐ADSC in the peri‐infarct area was determined by the number of tdTomato‐positive cells per total nuclei (*n* = 8 per group). Abbreviations: ADSC‐con: ADSC transfected with adenovirus‐control for 2 days. ADSC‐FXR: ADSC transfected with adenovirus‐FXR for 2 days. All data were analyzed using unpaired Student's *t*‐test. Data are presented as the mean ± SEM. ns: not significant.

We also used ADSC with red fluorescence isolated from tdTomato transgenic mice (tdTomato‐ADSC) for intramyocardial injection. In addition, tdTomato‐ADSC were also labeled with the lipophilic near infrared fluorescent dye DiR for whole heart imaging. The results revealed that the fluorescence intensity of the hearts in the MI+ADSC‐FXR group was significantly stronger than that in the MI+ADSC‐con group, regardless of 3 days or 7 days after injection (**Figure** **2**A,B). According to the location of DIR fluorescence, we obtained the corresponding tissue from the heart to observe the retention of tdTomato‐ADSC. The actual number of tdTomato‐ADSC in the heart 6 h, 1, 3, and 7 days after transplantation was determined via quantitative PCR assessments of tdTomato DNA levels. The results showed that at 6 h after intramyocardial injection, the injected ADSC accounted for ≈5% of the total heart cells. After that, the retention rate of ADSC gradually decreased, especially from 3 days after injection. From 1 day after injection, the engraftment rate of ADSC overexpressing FXR was significantly increased compared with that of ADSC‐con at different time points (Figure 2C). Immunofluorescence staining showed that compared with the MI+ADSC‐con group, there were more tdTomato‐positive cells in the heart sections from the MI+ADSC‐FXR group, both at 3 and 7 days after injection (Figure 2D,E). The results demonstrated that the engraftment/survival rate of ADSC in the MI+ADSC‐FXR group was significantly increased compared with that in the MI+ADSC‐con group. Notably, FXR overexpression significantly promotes the survival of implanted ADSC, prolongs ADSC retention in the infarct border zone and protects the ischemic heart.

### 2.3 FXR Overexpression Enhances the Paracrine Proangiogenic Effect of ADSC but Fails to Reduce Hydrogen Peroxide‐Induced ADSC Apoptosis

**Figure 3:**
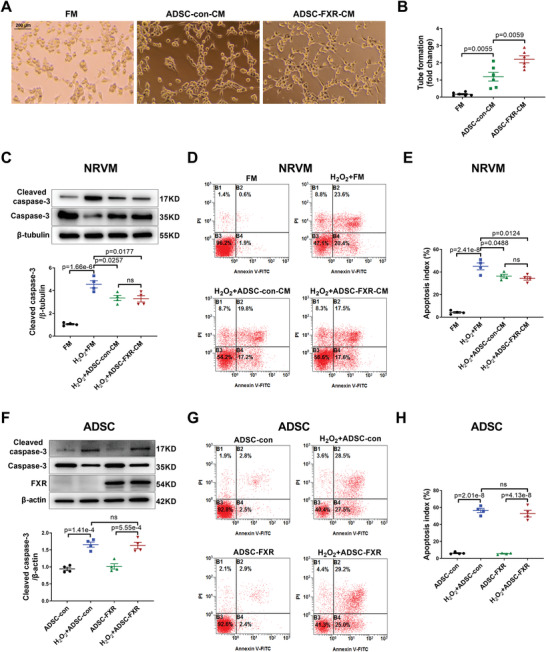
FXR overexpression enhanced the paracrine proangiogenic effect of ADSC but failed to affect the apoptosis and paracrine antiapoptotic effect of ADSC. A) A tube formation assay of RCAEC was performed to evaluate the paracrine proangiogenic capability of ADSC‐con and ADSC‐FXR, and representative images are shown. B) Quantification of tube formation (*n* = 6 per group). C) Representative western blotting images and quantification of protein expression of cleaved caspase‐3 and caspase‐3 in NRVM. The NRVM were incubated with FM, ADSC‐con‐CM, or ADSC‐FXR‐CM for 24 h followed by H_2_O_2_ (200 µm) treatment for another 8 h (*n* = 4 per group). D) Flow cytometric analysis of NRVM after incubation with FM, ADSC‐con‐CM, or ADSC‐FXR‐CM for 24 h followed by H_2_O_2_ (200 µm) treatment for another 8 h (*n* = 4 per group). E) NRVM apoptosis was quantified as the sum of the proportion of cells in quadrants B2 and B4 (*n* = 4 per group). F) Representative western blotting images and quantification of the protein expression of cleaved caspase‐3, caspase‐3, and FXR in ADSC. ADSC‐con and ADSC‐FXR were treated with H_2_O_2_ (200 µm) for 24 h (*n* = 4 per group). G) Flow cytometric analysis of ADSC‐con and ADSC‐FXR after treatment with H_2_O_2_ (200 µm) for 24 h (*n* = 4 per group). H) ADSC apoptosis was quantified as the sum of the proportion of cells in quadrants B2 and B4 (*n* = 4 per group). Abbreviations: ADSC‐con: ADSC transfected with adenovirus control for 2 days. ADSC‐FXR: ADSC transfected with adenovirus‐FXR for 2 days. RCAEC: rat coronary artery endothelial cells. FM: fresh medium. CM: conditioned medium. RCAEC: rat coronary artery endothelial cells. NRVM: neonatal rat ventricular myocytes. All data were analyzed using one‐way ANOVA, followed by Bonferroni post hoc test. Data are presented as the mean ± SEM. ns: not significant.

Because ADSC with FXR overexpression stimulated angiogenesis and reduced cardiomyocyte apoptosis after intramyocardial injection, we performed in vitro experiments to understand the cellular mechanisms by which FXR overexpression regulates ADSC‐mediated cardioprotection. First, we compared FXR expression in ADSC with that in several cell types in the heart (i.e., cardiomyocytes, endothelial cells, and fibroblasts).^[^ ^21^ ^]^ ADSC isolated from FXR KO mice were used as a negative control. The results showed that the expression of FXR in ADSC was similar to that in the above cells in the heart (Figure S4A,B, Supporting Information). Then, we performed a tube formation assay to determine whether FXR overexpression directly improves the paracrine proangiogenic effect of ADSC. Rat coronary artery endothelial cells (RCAEC) were seeded in Matrigel and then incubated with the following media: fresh medium (FM), conditioned medium (CM) of ADSC‐con (ADSC‐con‐CM), or CM of ADSC‐FXR (ADSC‐FXR‐CM), and then the number of tube formation was counted. As shown in **Figure** **3**A,B, compared to FM, ADSC‐con‐CM significantly increased tube formation. Notably, ADSC‐FXR‐CM resulted in a significantly higher number of tubes than ADSC‐con‐CM, indicating an enhanced paracrine proangiogenic effect of ADSC‐FXR (*p* < 0.01, Figure 3A,B). Next, we assessed whether ADSC overexpressing FXR have a direct paracrine function against cardiomyocyte apoptosis. Neonatal rat ventricular myocytes (NRVM) were treated with hydrogen peroxide (H_2_O_2_) after incubation with the above media. Western blotting and flow cytometry were used to detect cardiomyocyte apoptosis. Both ADSC‐con‐CM and ADSC‐FXR‐CM markedly ameliorated the apoptosis of NRVM treated with H_2_O_2_ (both *p* < 0.05 vs FM, Figure 3C–E). Compared to ADSC‐con‐CM, ADSC‐FXR‐CM did not further decrease NRVM apoptosis (*p* > 0.05, Figure 3C–E). These results suggested that FXR overexpression enhances the paracrine proangiogenic effect but not the paracrine antiapoptotic effect of ADSC.

Finally, we determined whether FXR overexpression affects ADSC apoptosis, proliferation, and differentiation. Unexpectedly, FXR overexpression failed to decrease cleaved caspase‐3 expression in ADSC treated with H_2_O_2_ (*p* > 0.05, Figure 3F). The apoptotic rate of ADSC was not significantly affected by FXR overexpression, as detected by flow cytometry (*p* > 0.05, Figure 3G,H). RT‐PCR demonstrated that FXR overexpression for 7 days did not significantly affect the cardiac, vasculogenic, myogenic, adipogenic, or osteogenic differentiation of ADSC (Figure S4C, Supporting Information). In addition, FXR overexpression for 7 days did not alter the mRNA levels of surface markers of MSC (Figure S4D, Supporting Information). Based on the Cell Counting Kit‐8 (CCK‐8) assay results, FXR overexpression did not increase ADSC proliferation (Figure S4E, Supporting Information). Collectively, these results revealed that FXR overexpression does not directly affect the apoptosis, proliferation, differentiation, or paracrine antiapoptotic effect of ADSC. Since we observed that FXR overexpression increased the ADSC retention/survival rate and reduced cardiomyocyte apoptosis in vivo, the effects of FXR on ADSC apoptosis and ADSC‐mediated antiapoptotic effects between in vivo and in vitro conditions were paradoxical.

### 2.4 FXR Overexpression Promotes ADSC Paracrine Angiogenesis via Angiopoietin‐Like Protein 4 (Angptl4)

**Figure 4:**
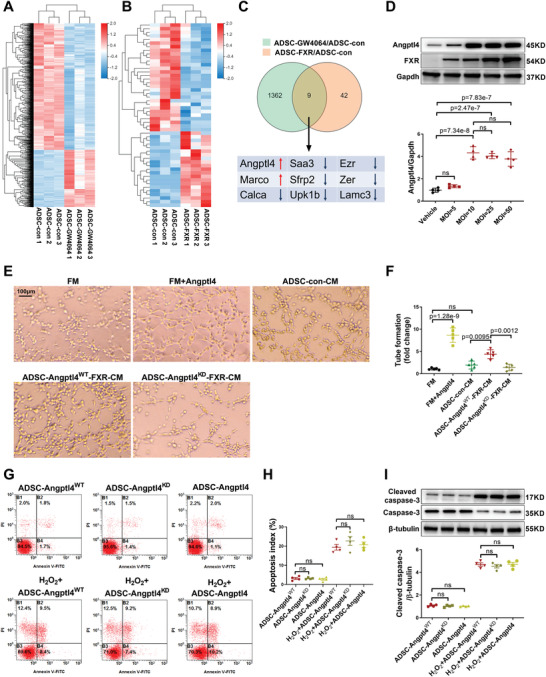
FXR overexpression promoted ADSC paracrine angiogenesis via Angptl4. A) The differential clustering heatmap between samples from ADSC‐con and ADSC‐GW4064 by RNA‐seq analysis (*n* = 3 per group). B) The differential clustering heatmap between samples from ADSC‐con and ADSC‐FXR according to RNA‐seq analysis (*n* = 3 per group). C) The Venn diagram of the changed genes for each of the two groups (ADSC‐FXR and ADSC‐GW4064) compared to ADSC‐con (*n* = 3 per group), and the 9 genes that changed with both interventions are shown. D) Representative western blotting images and quantification of Angptl4 and FXR protein expression in ADSC (*n* = 4 per group). E) Tube formation assay of RCAEC to evaluate the proangiogenic capability of Angptl4 and the paracrine proangiogenic effect of ADSC‐con, ADSC‐Angptl4^WT^‐FXR and ADSC‐Angptl4^KD^‐FXR. Representative images are shown. F) Quantification of tube formation (*n* = 5 per group). G) Flow cytometric analysis of ADSC‐Angptl4^WT^, ADSC‐Angptl4^KD^, and ADSC‐Angptl4 after treatment with H_2_O_2_ (200 µm) for 24 h (*n* = 4 per group). H) ADSC apoptosis was quantified as the sum of the proportion of cells in quadrants B2 and B4 (*n* = 4 per group). I) Representative western blotting images and quantification of the protein expression of cleaved caspase‐3 and caspase‐3 in ADSC. ADSC‐Angptl4^WT^, ADSC‐Angptl4^KD^, and ADSC‐Angptl4 were treated with H_2_O_2_ (200 µm) for 24 h (n = 4 per group). Abbreviations: ADSC‐con: ADSC transfected with adenovirus control for 2 days. ADSC‐FXR: ADSC transfected with adenovirus‐FXR for 2 days. ADSC‐GW4064: ADSC treated with GW4064 (10 µm) for 2 days. ADSC‐Angptl4^WT^‐FXR: ADSC transfected with scramble RNA for 24 h, followed by transfection with adenovirus‐FXR for another 2 days. ADSC‐Angptl4^KD^‐FXR: ADSC transfected with Angptl4 siRNA for 24 h, followed by transfection with adenovirus‐FXR for another 2 days. ADSC‐Angptl4^WT^: ADSC transfected with scramble RNA for 2 days. ADSC‐Angptl4^KD^: ADSC transfected with Angptl4 siRNA for 2 days. ADSC‐Angptl4: ADSC incubated with the recombinant Angptl4 protein (2.5 µm) for 2 days. FM: fresh medium. CM: conditioned medium. RCAEC: rat coronary artery endothelial cells. All data were analyzed using one‐way ANOVA, followed by Bonferroni post hoc test. Data are presented as the mean ± SEM. ns: not significant.

To clarify the molecular mechanisms responsible for FXR‐mediated ADSC cardioprotection, RNA sequencing (RNA‐seq) analysis was performed using RNA samples from the ADSC‐con, ADSC‐FXR, and ADSC treated with GW4064 (ADSC‐GW4064) groups. GW4064, a synthetic FXR agonist, is widely used to activate FXR.^[^ ^15^ ^]^ RNA‐seq analysis revealed 1371 differentially expressed genes between ADSC‐GW4064 and ADSC‐con samples and 51 differentially expressed genes between ADSC‐FXR and ADSC‐con samples (**Figure** **4**A,B). To identify genes that were simultaneously regulated by FXR overexpression and FXR activation, we performed Venn diagram analysis and found 9 common genes (Figure 4C). Among the proteins encoded by these 9 genes, only 2 exhibited elevated levels (Angptl4, Marco). As a secretory protein, Angptl4 has been identified as a multifunctional cytokine modulating important physiological events such as angiogenesis, vascular homeostasis, and inflammation.^[^ ^22^, ^23^, ^24^ ^]^

We thus assessed whether Angptl4 is involved in FXR‐mediated ADSC cardioprotection. As shown in Figure 4D, the protein expression of Angptl4 in ADSC was dose‐dependently increased by adenovirus‐FXR transfection (*p* < 0.01). To clarify whether FXR overexpression promoted the paracrine proangiogenic effect of ADSC via Angptl4, we transfected ADSC with small interfering RNA (siRNA) against Angptl4 or small conditional RNA (scRNA) 24 h before adenovirus‐FXR transfection (ADSC‐Angptl4^KD^‐FXR or ADSC‐Angptl4^WT^‐FXR) (Figure S4F,G, Supporting Information). Angptl4 siRNA largely abolished the proangiogenic effect of ADSC‐FXR‐CM (*p* < 0.01, Figure 4E,F). In contrast, the recombinant Angptl4 protein significantly increased the tube formation of RCAEC (*p* < 0.01, Figure 4E,F). However, both western blotting and flow cytometry revealed that neither siRNA nor recombinant Angptl4 protein affected H_2_O_2_‐induced ADSC apoptosis, suggesting that Angptl4 did not promote ADSC survival (Figure 4G–I). Taken together, these results demonstrated that FXR overexpression enhances the paracrine proangiogenic effect of ADSC via Angptl4.

### 2.5 When Binding a Synthetic Bile Acid, ADSC Overexpressing FXR Show Reduced Hydrogen Peroxide‐Induced Apoptosis and Further Enhance the Paracrine Proangiogenic Effect

**Figure 5:**
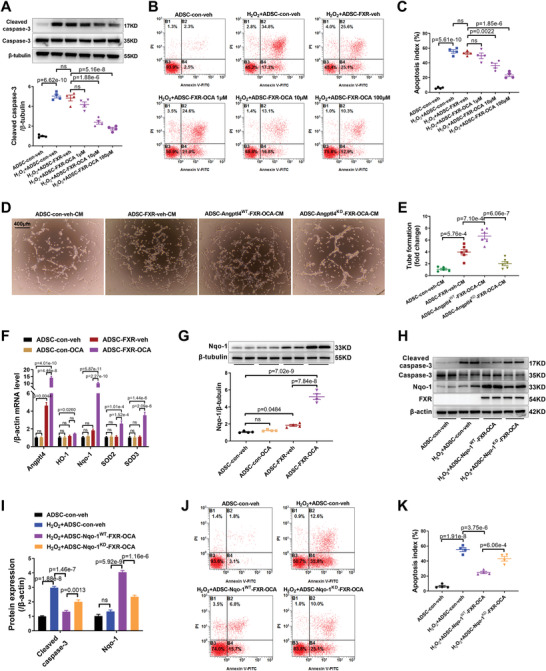
FXR overexpression was able to reduce ADSC apoptosis in the presence of its ligands, and bile acid‐FXR signal activation promoted ADSC survival via Nqo‐1. A) Representative western blotting images and quantification of the protein expression of cleaved caspase‐3 and caspase‐3 in ADSC. ADSC‐con‐veh, ADSC‐FXR‐veh and ADSC‐FXR‐OCA were treated with H_2_O_2_ (200 µm) for 24 h (*n* = 4 per group). B) Flow cytometric analysis of ADSC‐con‐veh, ADSC‐FXR‐veh, and ADSC‐FXR‐OCA after treatment with H_2_O_2_ (200 µm) for 24 h. C) ADSC apoptosis was quantified as the sum of the proportion of cells in quadrants B2 and B4 (*n* = 4 per group). D) Tube formation assay of RCAEC was determined to evaluate the capability of paracrine proangiogenesis of ADSC‐con‐veh, ADSC‐FXR‐veh, ADSC‐Angptl4^WT^‐FXR‐OCA and ADSC‐Angptl4^KD^‐FXR‐OCA, and representative images are shown. E) Quantification of tube formation (*n* = 5, 6, 6, 6). F) Angptl4, HO‐1, Nqo‐1, SOD2, and SOD3 mRNA levels in ADSC were determined by RT‐PCR (*n* = 5 per group). G) Representative western blotting images and quantification of Nqo‐1 protein expression in ADSC (*n* = 4 per group). H,I) Representative western blotting images and quantification of the protein expression of cleaved caspase‐3, caspase‐3, Nqo‐1, and FXR in ADSC. ADSC‐con‐veh, ADSC‐Angptl4^WT^‐FXR‐OCA, and ADSC‐Angptl4^KD^‐FXR‐OCA were treated with H_2_O_2_ (200 µm) for 24 h (*n* = 4 per group). J) Flow cytometric analysis of ADSC‐con‐veh, ADSC‐Angptl4^WT^‐FXR‐OCA, and ADSC‐Angptl4^KD^‐FXR‐OCA after treatment with H_2_O_2_ (200 µm) for 24 h. K) ADSC apoptosis was quantified as the sum of the proportion of cells in quadrants B2 and B4 (*n* = 4 per group). Abbreviations: OCA: obeticholic acid. ADSC‐con‐veh: ADSC transfected with adenovirus control for 24 h, followed by incubation with vehicle for another 24 h. ADSC‐FXR‐veh: ADSC transfected with adenovirus‐FXR for 24 h, followed by incubation with vehicle for another 24 h. ADSC‐FXR‐OCA: ADSC transfected with adenovirus‐FXR for 24 h, followed by incubation with 10 µm OCA for another 24 h. ADSC‐Angptl4^WT^‐FXR‐OCA: ADSC transfected with scramble RNA for 24 h and then transfected with adenovirus‐FXR for another 24 h, followed by incubation with 10 µm OCA for another 24 h. ADSC‐Angptl4^KD^‐FXR‐OCA: ADSC transfected with Angptl4 siRNA for 24 h and then transfected with adenovirus‐FXR for another 24 h, followed by incubation with 10 µm OCA for another 24 h. ADSC‐Nqo‐1^WT^‐FXR‐OCA: ADSC transfected with scramble RNA for 24 h and then transfected with adenovirus‐FXR for another 24 h, followed by incubation with 10 µM OCA for another 24 h. ADSC‐Nqo‐1^KD^‐FXR‐OCA: ADSC transfected with Nqo‐1 siRNA for 24 h and then transfected with adenovirus‐FXR for another 24 h, followed by incubation with 10 µM OCA for another 24 h. FM: fresh medium. CM: conditioned medium. RCAEC: rat coronary artery endothelial cells. All data were analyzed using one‐way ANOVA, followed by Bonferroni post hoc test. Data are presented as the mean ± SEM. ns: not significant.

The above results revealed that FXR overexpression improved the antiapoptotic ability of intramyocardially injected ADSC (Figure S3A–D, Supporting Information) but failed to directly suppress H_2_O_2_‐induced ADSC apoptosis in vitro (Figure 3F–H). Next, we sought to explain the paradoxical effects of FXR overexpression on ADSC apoptosis under in vivo and in vitro conditions. Since FXR is one of the receptors for bile acids, we hypothesized that FXR overexpression may increase ADSC survival by responding to its ligands. Obeticholic acid (OCA) is widely used as a synthetic FXR ligand and is a semisynthetic hydrophobic bile acid analog. OCA is a highly selective FXR agonist with an activation potency similar to endogenous chenodeoxycholic acid.^[^ ^25^ ^]^ Indeed, compared with FXR overexpression alone, FXR overexpression combined with OCA dose‐dependently decreased the level of cleaved caspase‐3 expression in ADSC (*p* < 0.01, **Figure** **5**A) and the apoptotic rate of ADSC (*p* < 0.01, Figure 5B,C) treated with H_2_O_2_. However, OCA administration without FXR overexpression did not decrease the level of cleaved caspase‐3 expression in ADSC or the apoptotic rate of ADSC treated with H_2_O_2_ (*p* > 0.05, Figure S5A–C, Supporting Information). These results suggested that FXR overexpression increases ADSC survival when its ligand is present.

In addition, we tested whether OCA administration increases the paracrine function of ADSC‐FXR. As shown in Figure 5D,E, CM of ADSC‐Angptl4^WT^‐FXR‐OCA (ADSC‐Angptl4^WT^‐FXR‐OCA‐CM) exhibited a stronger proangiogenic effect than ADSC‐FXR‐CM. Consistently, Angptl4 siRNA largely counteracted the proangiogenic effect of ADSC‐Angptl4^WT^‐FXR‐OCA‐CM (*p* < 0.01, Figure 5D,E). Western blotting and flow cytometric studies showed that compared to ADSC‐con‐veh‐CM, ADSC‐FXR‐OCA‐CM did not decrease cleaved caspase‐3 expression in NRVM or the apoptosis rate of NRVM treated with H_2_O_2_ (*p* > 0.05, Figure S5D–F, Supporting Information). In addition, FXR overexpression combined with OCA administration did not significantly affect ADSC proliferation (Figure S5G, Supporting Information) or the cardiac, vasculogenic, myogenic, adipogenic, or osteogenic differentiation of ADSC (Figure S5H, Supporting Information).

To further verify the molecules responsible for the cardiac protection present in ADSC‐FXR‐OCA‐CM, LC‐MS/MS was performed. The results revealed that ADSC‐FXR‐OCA increased 160 proteins but decreased 189 proteins compared to ADSC‐con (fold change>3, *p* < 0.0001; Figure S6A and Tables of LC‐MS/MS, Supporting Information). Importantly, Angptl4 was increased by 3.33‐fold in ADSC‐FXR‐OCA‐CM compared to ADSC‐con‐CM (*p* = 6.8e‐5, Figure S6B, Supporting Information). In addition, the proteins present in CM were also determined by western blotting. Consistent with the LC‐MS/MS results, the total protein concentration was significantly increased in ADSC‐FXR‐OCA‐CM compared with ADSC‐con‐CM (Figure S6C,D, Supporting Information). Compared with ADSC‐con‐CM, the Angptl4 level was markedly increased in ADSC‐FXR‐OCA‐CM (Figure S6E, Supporting Information). These results further confirmed that ADSC‐FXR played a strong paracrine proangiogenic role through Angptl4.

Many studies have shown that exosomes mediate the cardioprotection of MSC in MI. To assess whether exosomes played a role in the paracrine effects of ADSC‐FXR‐OCA, we first extracted and detected exosomes in the CM of ADSC‐con and ADSC‐FXR‐OCA. The results revealed that bile acid‐FXR axis activation does not alter the size of exosomes, but it increases the number of exosomes secreted from ADSC by nearly threefold (Figure S7A–D, Supporting Information). Second, we extracted proteins from CM and exosomes and tested Angptl4 expression by western blotting. Although Angptl4 protein expression was significantly increased in the supernatant from ADSC‐FXR‐OCA compared with that from ADSC‐con, Angptl4 was not detected in the exosomal proteins of the two groups (Figure S7E, Supporting Information). Finally, we treated ADSC with GW4869, an inhibitor of exosome release. GW4869 did not significantly affect the paracrine proangiogenic effect of ADSC with bile acid‐FXR axis activation (Figure S7F,G, Supporting Information). Based upon the above experiments, we concluded that although ADSC‐FXR‐OCA released more exosomes, they did not play an important role in the proangiogenic effect of ADSC‐FXR‐OCA.

### 2.6 Bile Acid‐FXR Signal Activation Promotes ADSC Survival via NADPH Quinone Oxidoreductase‐1 (Nqo‐1)

Having demonstrated that FXR overexpression combined with OCA administration significantly reduced H_2_O_2_‐induced ADSC apoptosis, we explored the molecular mechanism(s) involved. Increased oxidative stress is one of the key features of acute MI and is directly tied to ADSC apoptosis.^[^ ^26^, ^27^ ^]^ Previous studies have uncovered the antioxidative role of FXR activation in liver or renal cells.^[^ ^28^, ^29^, ^30^, ^31^ ^]^ To clarify whether bile acid‐FXR signal activation affects oxidative stress, several important antioxidative stress molecules were assessed in ADSC‐con/ADSC‐FXR treated with vehicle or OCA. Angptl4 was used as a positive indicator. RT‐PCR demonstrated that compared to ADSC‐con‐veh, the mRNA level of Angptl4 was significantly increased in ADSC‐FXR‐veh (*p* < 0.01, Figure 5F). However, FXR overexpression did not significantly alter the mRNA expression of HO‐1, Nqo‐1, Sod‐2, or Sod‐3 compared to ADSC‐con‐veh (Figure 5F). Interestingly, OCA administration further increased the transcription of Angptl4, Nqo‐1, Sod‐2, and Sod‐3 in ADSC overexpressing FXR (*p* < 0.05 or *p* < 0.01, Figure 5F). We assessed the four antioxidants (HO‐1, Nqo‐1, Sod‐2, and Sod‐3) by western blotting analysis and found that only Nqo‐1 protein expression was significantly increased in the ADSC‐FXR‐OCA group compared to the ADSC‐FXR‐veh group (Figure 5G; and Figure S8A,B, Supporting Information).

To determine whether upregulation of Nqo‐1 is responsible for the increased resistance to apoptosis of ADSC‐FXR‐OCA, ADSC were transfected with Nqo‐1 siRNA or scRNA 24 h before adenovirus‐FXR transfection and OCA incubation (ADSC‐Nqo‐1^KD^‐FXR‐OCA or ADSC‐Nqo‐1^WT^‐FXR‐OCA). Nqo‐1 siRNA significantly reduced the mRNA and protein levels of Nqo‐1 in ADSC (*p* < 0.01, Figure S8C (Supporting Information); Figure 5H,I). Consistent with Figure 5C, both western blotting and flow cytometry showed that bile acid‐FXR signal activation significantly inhibited H_2_O_2_‐induced ADSC apoptosis (*p* < 0.01, Figure 5H‐K). More importantly, Nqo‐1 siRNA largely abolished the antiapoptotic effect of bile acid‐FXR signal activation in ADSC (*p* < 0.01, Figure 5H–K). These results suggested that bile acid‐FXR signal activation promotes ADSC survival under oxidative stress via Nqo‐1.

### 2.7 Endogenous Bile Acids in the Ischemic Heart Enhance the Survival and Paracrine Function of ADSC‐FXR

**Figure 6:**
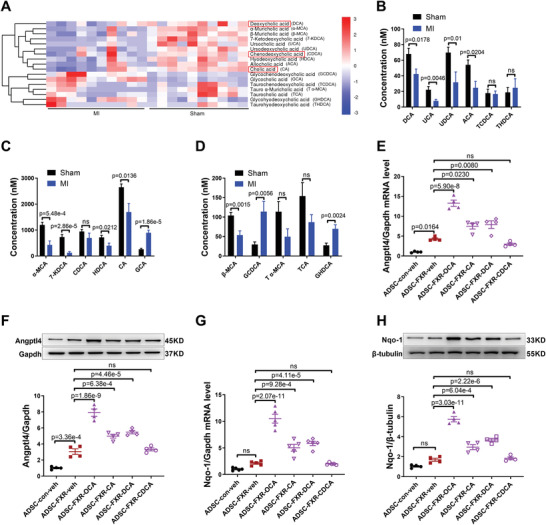
Endogenous CA and DCA in the ischemic heart enhanced the retention and paracrine function of ADSC‐FXR. A) Differential heatmap of bile acids in the peri‐infarct area of sham and MI mice according to targeted metabolomics analysis (*n* = 10 per group). B–D) The concentration of different bile acids in the peri‐infarct area of sham and MI mice (*n* = 10 per group). E) Angptl4 mRNA levels were determined by RT‐PCR (*n* = 4 per group). F) Representative western blotting images and quantification of Angptl4 protein expression in ADSC (*n* = 4 per group). G) Nqo‐1 mRNA levels were determined by RT‐PCR (*n* = 5 per group). H) Representative western blotting images and quantification of Nqo‐1 protein expression in ADSC (*n* = 4 per group). Abbreviations: OCA: obeticholic acid. ADSC‐con‐veh: ADSC transfected with adenovirus control for 24 h, followed by incubation with vehicle for another 24 h. ADSC‐FXR‐veh: ADSC transfected with adenovirus‐FXR for 24 h, followed by incubation with vehicle for another 24 h. ADSC‐FXR‐OCA: ADSC transfected with adenovirus‐FXR for 24 h, followed by incubation with 10 µm OCA for another 24 h. ADSC‐FXR‐CA: ADSC transfected with adenovirus‐FXR for 24 h, followed by incubation with 10 µm CA for another 24 h. ADSC‐FXR‐DCA: ADSC transfected with adenovirus‐FXR for 24 h, followed by incubation with 10 µm DCA for another 24 h. ADSC‐FXR‐CDCA: ADSC transfected with adenovirus‐FXR for 24 h, followed by incubation with 10 µm CDCA for another 24 h. All data were analyzed using one‐way ANOVA, followed by Bonferroni post hoc test. Data are presented as the mean ± SEM. ns: not significant.

According to the above results, FXR overexpression increased ADSC survival only in the presence of its ligands in vitro. However, FXR overexpression alone significantly increased the ADSC retention rate after intramyocardial injection in vivo. We thus hypothesized that ADSC‐FXR responds to endogenous bile acids in the myocardial ischemic microenvironment. To prove this hypothesis, targeted metabolomics was performed. The levels of 41 bile acids were measured in normal heart tissue collected from the sham MI group or peri‐infarcted heart tissue collected from the MI group at 7 days after MI surgery (*n* = 10 per group). Targeted metabolomics detected 19 bile acids in the heart tissue, and most of them showed a downward trend after MI (**Figure** **6**A–D). There are 4 reported to be the most effective endogenous ligands of FXR: cholic acid (CA), deoxycholic acid (DCA), lithocholic acid (LCA), and chenodeoxycholic acid (CDCA).^[^ ^32^ ^]^ Three of them (CA, DCA, CDCA) were detected under our experimental conditions (Figure 6A–D). More importantly, we treated ADSC‐FXR with these bile acids and found that two of them (CA and DCA) significantly increased the mRNA and protein expression of Angptl4 (*p* < 0.05 or *p* < 0.01, Figure 6E,F) and Nqo‐1 (*p* < 0.01, Figure 6G,H). To further demonstrate the significance of FXR overexpression in ADSC when bile acid levels decreased after MI, we incubated ADSC‐FXR with a mixture of reportedly effective ligands of FXR (CA, DCA, ursocholic acid (UCA), ursodeoxycholic acid (UDCA), and allocholic acid (ACA)),^[^ ^33^ ^]^ and the concentrations of each ligand were identical to the results of post‐MI metabolomics analysis. The results showed that the mixture of bile acids significantly promoted ADSC‐FXR survival and paracrine angiogenesis (Figure S9A–E, Supporting Information). In addition, the mixture of bile acids also significantly inhibited H_2_O_2_‐induced NRVM apoptosis (Figure S9F–H, Supporting Information). Immunostaining for Ki67 demonstrated that FXR overexpression did not affect ADSC proliferation in the presence of bile acids at physiological concentrations (Figure S9I,J, Supporting Information). These results revealed that endogenous bile acids in the ischemic heart promote cardiomyocyte survival and enhance the retention and paracrine proangiogenic effect of ADSC‐FXR.

### 2.8 FXR Overexpression‐Mediated Biological Effects of ADSC were Dependent on the Heterodimer Formed with Retinoid X Receptor *α* (RXR*α*)

FXR, a member of the nuclear hormone receptor superfamily, is also an important nuclear transcription factor. In most instances in vivo, FXR regulates downstream gene expression by forming a heterodimer with RXR*α* (Figure S10A, Supporting Information).^[^ ^32^, ^34^ ^]^ The coimmunoprecipitation assay showed that FXR binds to RXR*α* in ADSC‐FXR but not in ADSC‐con (Figure S10B, Supporting Information). To further clarify whether RXR*α* participates in the antiapoptotic and proangiogenic effects of bile acid‐FXR signal activation, in vivo and in vitro experiments with RXR*α* siRNA were performed. The mRNA and protein levels of RXR*α* were significantly decreased at 3 and 5 days after siRNA transfection (*p* < 0.01, Figure S10C,D, Supporting Information).

**Figure 7:**
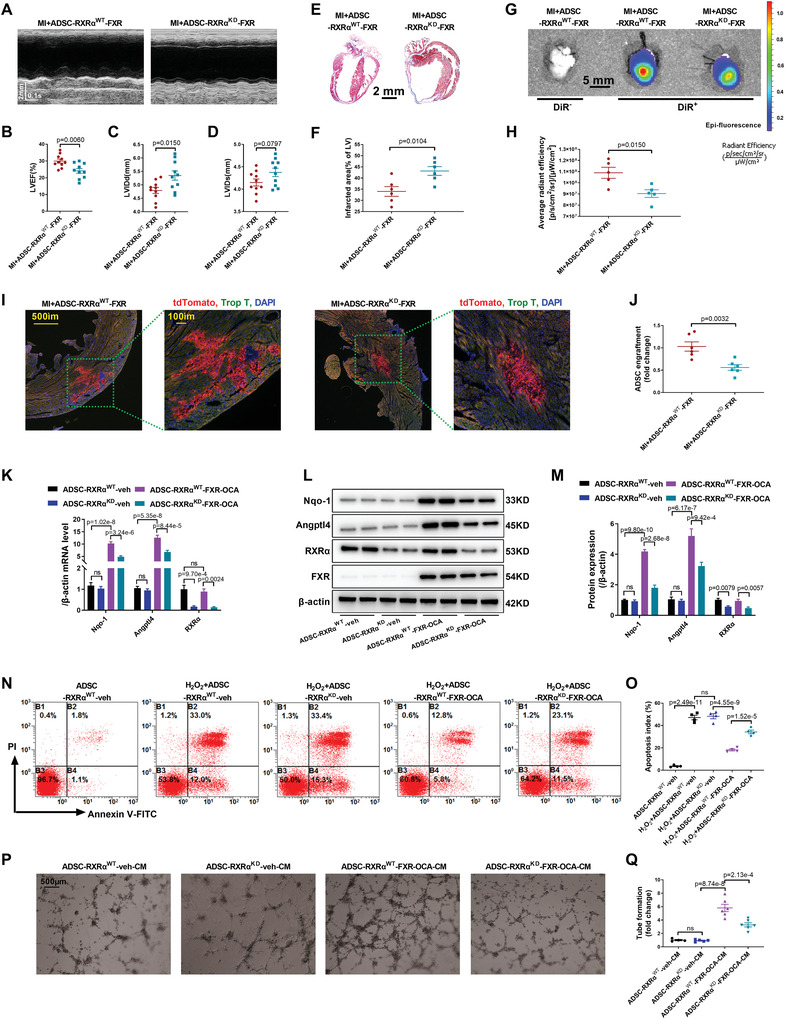
RXR*α* is important for the FXR overexpression‐mediated biological effects of ADSC. A) Cardiac function was evaluated by short‐axis M‐mode echocardiography 4 weeks after MI, and representative images are shown. B–D) LVEF, LVIDd, and LVIDs were evaluated by short‐axis M‐mode echocardiography. B–D) *n* = 10 per group. E) The myocardial infarct area 4 weeks after MI was determined by Masson's trichrome staining. Representative images were shown. F) Quantification of the fibrotic area 4 weeks after MI (*n* = 6 per group). G) Representative images of fluorescence intensity at 3 days after injection with DiR‐labeled ADSC. H) Quantification of DiR‐labeled ADSC was determined by the average radiant efficiency (*n* = 5 per group). I) Representative images of tdToamto‐ADSC in hearts 3 days after MI. Heart tissue was immunostained for troponin T (green) and DAPI (blue). J) Quantification of tdTomato‐ADSC in the peri‐infarct area was determined by the number of tdTomato‐positive cells per total nuclei (*n* = 6 per group). K) RXR*α*, Nqo‐1, and Angptl4 mRNA levels were determined by RT‐PCR (*n* = 4 per group). L,M) Representative western blotting images and quantification of Nqo‐1, Angptl4, RXR*α*, and FXR protein expression in ADSC (*n* = 4 per group). N) Flow cytometric analysis of ADSC‐RXRα^WT^‐veh, ADSC‐RXRα^KD^‐veh, ADSC‐RXRα^WT^‐FXR‐OCA, and ADSC‐RXRα^KD^‐FXR‐OCA after treatment with H_2_O_2_ (200 µm) for 24 h. O) ADSC apoptosis was quantified as the sum of the proportion of cells in quadrants B2 and B4 (*n* = 4 per group). P) A tube formation assay of RCAEC was performed to evaluate the paracrine proangiogenic effect of ADSC‐RXR^WT^‐veh, ADSC‐RXR^KD^‐veh, ADSC‐RXRα^WT^‐FXR‐OCA, and ADSC‐RXRα^KD^‐FXR‐OCA, and representative images are shown. Q) Quantification of tube formation (*n* = 4, 5, 6, 6). Abbreviations: OCA: Obeticholic acid. ADSC‐RXRα^WT^‐FXR: ADSC transfected with scramble RNA for 24 h and then transfected with adenovirus‐FXR for another 24 h. ADSC‐RXRα^KD^‐FXR: ADSC transfected with RXRα siRNA for 24 h and then transfected with adenovirus‐FXR for another 24 h. ADSC‐RXRα^WT^‐veh: ADSC transfected with scramble RNA for 3 days. ADSC‐RXRα^KD^‐veh: ADSC transfected with RXRα siRNA for 3 days. ADSC‐RXRα^WT^‐FXR‐OCA: ADSC transfected with scramble RNA for 24 h and then transfected with adenovirus‐FXR for another 24 h, followed by incubation with 10 µm OCA for another 24 h. ADSC‐RXRα^KD^‐FXR‐OCA: ADSC transfected with RXRα siRNA for 24 h and then transfected with adenovirus‐FXR for another 24 h, followed by incubation with 10 µm OCA for another 24 h. FM: fresh medium. CM: conditioned medium. RCAEC: rat coronary artery endothelial cells. All data were analyzed using one‐way ANOVA, followed by Bonferroni post hoc test. Data are presented as the mean ± SEM. ns: not significant.

First, ADSC were transfected with RXR*α* siRNA or scRNA 24 h before control adenovirus or Ad‐FXR transfection (ADSC‐RXR*α* ^WT^‐veh, ADSC‐RXR*α* ^KD^‐veh, ADSC‐RXR*α* ^WT^‐FXR, or ADSC‐RXR*α* ^KD^‐FXR). ADSC‐RXR*α* ^WT^‐FXR and ADSC‐RXR*α* ^KD^‐FXR were intramyocardially injected into mice immediately after MI surgery. Echocardiography showed that intramyocardial delivery of ADSC‐RXR*α* ^KD^‐FXR significantly decreased cardiac function compared to ADSC‐RXR*α* ^WT^‐FXR after MI surgery (**Figure** **7**A–D). Masson's trichome staining showed that RXR*α* siRNA significantly increased myocardial infarct size compared to ADSC‐RXR*α* ^WT^‐FXR treatment (*p* < 0.05; Figure 7E,F). Quantification of CM‐DiI‐labeled cells and the fluorescence intensity of DiR‐labeled cells both revealed that the retention rate of ADSC‐RXR*α* ^KD^‐FXR was significantly lower than that of ADSC‐RXR*α* ^WT^‐FXR (*p* < 0.01 or *p* < 0.05, time point MI 3 days; Figure S10E,F (Supporting Information), Figure 7G,H). In addition, we verified that RXR*α* siRNA attenuated the retention of ADSC with FXR overexpression by tdTomato‐ADSC quantification (Figure 7I,J).

Next, we tested whether RXR*α* siRNA affects the levels of Nqo‐1 and Angptl4, the two key downstream molecules of bile acid‐FXR signaling. RXR*α* siRNA did not affect the expression of Angptl4 and Nqo‐1 in control ADSC, but it significantly downregulated Nqo‐1 and Angptl4 expression after bile acid‐FXR signaling activation (*p* < 0.01, Figure 7K–M). Finally, at the cellular level, flow cytometry showed that RXR*α* siRNA did not change the H_2_O_2_‐induced apoptosis of ADSC in the control group, but it increased the H_2_O_2_‐induced ADSC apoptosis rate after bile acid‐FXR axis activation (*p* < 0.01, Figure 7N,O). In addition, tube formation assays showed that RXR*α* siRNA did not affect the proangiogenic effect of ADSC in the control group, but it partly blocked the paracrine proangiogenic effect of ADSC‐RXR*α* ^WT^‐FXR‐OCA (*p* < 0.01, Figure 7P,Q). Taken together, these results demonstrated that FXR overexpression‐mediated antiapoptotic and proangiogenic effects of ADSC after bile acid‐FXR signaling activation were dependent on the heterodimer formed with RXR*α*.

### 2.9 The Transcription of Angptl4 and Nqo‐1 was Directly Regulated by FXR

To clarify whether FXR directly regulate Angptl4 and Nqo‐1 expression at the transcriptional level, chromatin immunoprecipitation (ChIP), and dual luciferase reporter assays were performed. FXR‐associated DNA fragments were immunoprecipitated using anti‐FXR antibody. The PCR results of the ChIP assay revealed that anti‐FXR antibody precipitated proteins bound to the amplified sequence of the Angptl4 and Nqo‐1 promoter regions, as indicated by the blots for both the ADSC‐con and ADSC‐FXR‐OCA groups (Figure S10G,H, Supporting Information).

The mouse full‐length Angptl4 or Nqo‐1 promoter (‐2000‐0) was cloned into the PGL3.0‐Basic vector upstream of the luciferase cassette (PGL‐FL‐Angptl4 promoter reporter or PGL‐FL‐Nqo‐1 promoter reporter). As shown in Online Figure 10I,J, the PGL‐FL‐Angptl4 or PGL‐FL‐Nqo‐1 promoter reporter was cotransfected with Ad‐FXR or Ad‐con into HEK‐293T cells. In Ad‐con transfected HEK‐293T cells, the luciferase activity of PGL‐FL‐Angptl4 promoter was significantly higher than PGL‐Basic, while the luciferase activity of PGL‐FL‐Nqo‐1 promoter was equal to PGL‐Basic. However, Ad‐FXR+OCA treatment robustly increased the relative luciferase activity of PGL‐FL‐Angptl4 and PGL‐FL‐Nqo‐1 promoter reporters separately in HEK‐293T cells (Figure S10I,J, Supporting Information). These results demonstrated that FXR directly increases Angptl4 and Nqo‐1 transcription.

## 3 Discussion

In the past decade, there have been impressive advances in stem cell‐based therapy for ischemic heart diseases.^[^ ^35^ ^]^ The results achieved in this field are apparent despite ongoing controversies. Some studies have shown that MSC or their secreted extracellular vesicles significantly improve cardiac function after MI, but some other studies have revealed that MSC do not provide significant myocardial protection.^[^ ^36^, ^37^, ^38^, ^39^ ^]^ The reason for the contradiction is mainly due to rapid loss and insufficient paracrine function. Previous studies showed that the low cell survival/retention (low quantity) and deficient paracrine function (poor quality) of MSC after transplantation are the main limitations of their clinical efficacy.^[^ ^6^, ^11^, ^40^ ^]^ Recent studies have confirmed that increasing the antiapoptotic or adhesion properties could significantly improve the retention rate of MSC and that enhancing paracrine function could improve the paracrine cardioprotective potential of MSC.^[^ ^6^, ^11^, ^18^, ^41^, ^42^ ^]^ We previously identified CTRP9 as a cardiokine that promotes the survival and cardioprotection of implanted MSC, highlighting the important role of the ischemic microenvironment in MSC‐based therapy.^[^ ^43^ ^]^ In the present study, we made several novel observations.

First, we clarified that FXR overexpression significantly enhanced the cardioprotective effects of ADSC against post‐MI cardiac remodeling and dysfunction. Nuclear receptors are transcriptional regulators that exist widely in organisms. It was reported that FXR, a nuclear receptor, is a critical regulator of intestinal cancer stem cell proliferation and may be a potential therapeutic target for treating colorectal cancer.^[^ ^44^ ^]^ However, the role of FXR in MSC‐based therapy for heart repair has never been studied. In the present study, we demonstrated that FXR overexpression significantly increased the myocardial retention rate of engrafted ADSC and improved the cardioprotective effects of ADSC against ischemic heart injury, as evidenced by improved cardiac function, reduced myocardial infarct size, increased survival rate, enhanced angiogenesis, and reduced cardiomyocyte apoptosis. FXR ablation did not alter ADSC retention/survival significantly in vivo, which might be due to the low basal expression level of FXR in ADSC. Our results indicate for the first time that low FXR expression limits its cardioprotective ability and that FXR is an important nuclear receptor that enhances ADSC cardioreparative potential.

Second, we demonstrated that bile acids present in the myocardial microenvironment are critical to the myocardial repair function of ADSC‐FXR. Inconsistent with the in vivo results, FXR overexpression failed to improve ADSC survival in vitro and exerted limited paracrine proangiogenic effects. Compared with the cell culture environment in vitro, the myocardial ischemic microenvironment in vivo is more complicated.^[^ ^26^, ^45^ ^]^ There is increasing evidence that multiple metabolic disorders occur in the progression of myocardial ischemia, such as glucose, branched chain amino acids, and lipid metabolism disorders, and those metabolic alterations further influence disease progression.^[^ ^46^, ^47^, ^48^, ^49^, ^50^ ^]^ It has been reported that as one of the central tenets of microbe‐host crosstalk, bile acids have the potential to influence host cardiometabolic health.^[^ ^51^ ^]^ As one of the crucial receptors for bile acids, FXR plays an important role in regulating bile acid, glucose, and lipid metabolism homeostasis.^[^ ^14^, ^52^, ^53^ ^]^ In a recent study, it was reported that the activation of TGR5 (another receptor of bile acid) by bile acid signaling significantly improved intestinal stem cell and epithelial cell regeneration, thereby promoting the repair of the intestine after injury.^[^ ^54^ ^]^ Our experiments confirmed that FXR overexpression combined with OCA administration could significantly promote ADSC survival under oxidative stress and further enhance the angiogenic effect of MSC.

Targeted metabolomics analysis showed that 19 bile acids were present in the mouse cardiac tissue, and most of them showed a downward trend after MI. Despite this, their levels were still on the same order of magnitude before and after MI. More importantly, two of the 19 bile acids (CA and DCA) significantly enhanced Angptl4 and Nqo‐1 expression in ADSC‐FXR. Wang et al. reported that bile acid levels were altered in the plasma of MI patients and that most bile acids were reduced post‐MI. They further elucidated that TGR5 activated by DCA reduced inflammatory responses and ameliorated cardiac dysfunction after MI.^[^ ^55^ ^]^ Another study showed that ursodeoxycholic acid (UDCA) could improve peripheral blood flow and liver function in patients with chronic heart failure.^[^ ^56^ ^]^ Due to the low FXR expression, which is hardly detectable by western blotting, the injected MSC could not respond to bile acid signals in the microenvironment. Here, we demonstrated for the first time that implanted ADSC with FXR overexpression could respond to bile acids in the myocardial ischemic microenvironment and that bile acid‐FXR axis activation significantly improves ADSC retention and cardioprotection, including suppression of cardiomyocyte apoptosis and promotion of angiogenesis.

Third, we revealed that Angptl4 upregulation is responsible for the paracrine angiogenic effect of bile acid‐FXR axis activation. Proangiogenic therapy facilitates the de novo formation of microvessels in the peri‐infarcted area and has the potential to improve the blood supply and salvage ischemic‐damaged cardiomyocytes.^[^ ^57^ ^]^ In recent years, a large number of studies have confirmed that various extracellular vesicles, microRNAs, or proteins can reduce ischemic damage after MI by promoting myocardial angiogenesis, thereby exerting myocardial protection and improving cardiac function.^[^ ^58^, ^59^, ^60^, ^61^, ^62^, ^63^ ^]^ In the present study, RNA‐seq screening followed by RT‐qPCR/western blotting and LC–MS/MS suggested that bile acid‐FXR axis activation significantly increases Angptl4 expression and secretion. However, the exosome inhibitor GW4869 did not significantly affect the paracrine proangiogenic effect of ADSC‐FXR‐OCA, which revealed that bile acid‐FXR axis activation promotes Angptl4 expression and secretion through a non‐exosome dependent pathway, thus playing a paracrine role in promoting angiogenesis. The role of Angptl4 in angiogenesis is controversial, and both proangiogenic and antiangiogenic effects have been reported.^[^ ^64^, ^65^, ^66^, ^67^ ^]^ Recently, mounting evidence has demonstrated that Angptl4 is a proangiogenic factor in most cases.^[^ ^24^, ^68^ ^]^ Angptl4 exhibits a proangiogenic effect in both VEGF‐dependent and VEGF‐independent manners.^[^ ^64^ ^]^ The antiangiogenic effect of Angptl4 may be ascribed to the posttranslational modification of the protein in the special microenvironment.^[^ ^69^ ^]^ In our experiment, we found that the Angptl4 recombinant protein has a strong effect on angiogenesis. Gene silencing of Angptl4 in ADSC reduced the ability of FXR overexpression to promote angiogenesis. Our results demonstrate for the first time the dependency of the paracrine proangiogenic effect of the bile acid‐FXR axis on Angptl4 upregulation.

Fourth, we demonstrated that FXR overexpression could increase the resistance of ADSC to oxidative stress injury in the presence of its ligands. It was believed that moderate hypoxia preconditioning of MSC could improve their therapeutic potential.^[^ ^70^, ^71^ ^]^ However, as one of the key features in the myocardial ischemic microenvironment, excessive oxidative stress is directly harmful to ADSC survival.^[^ ^26^ ^]^ Inconsistent with the in vivo results, cellular experiments failed to confirm that FXR overexpression promoted ADSC survival under oxidative stress. However, in the presence of bile acids, ADSC‐FXR exhibited a stronger antioxidative damage ability. FXR overexpression combined with OCA administration significantly increased Nqo‐1 expression. Nqo‐1 gene silencing abolished the cardioprotective effect of ADSC through bile acid‐FXR axis activation. Earlier studies demonstrated that Nqo‐1 counteracts intracellular ROS generation, thus attenuating DNA damage and improving cell survivability.^[^ ^26^, ^72^ ^]^ Our experimental results confirm for the first time that bile acid‐FXR axis activation significantly improves the antiapoptotic effect of ADSC by promoting Nqo‐1 expression, thus increasing the quantity of transplanted MSC.

Finally, we revealed that FXR overexpression‐mediated biological effects of ADSC were dependent on the heterodimer formed with RXR*α*, and the transcription of Angptl4 and Nqo‐1 was directly regulated by FXR. As an important metabolic regulator and transcription factor, FXR regulates the expression of most downstream genes in the form of a heterodimer.^[^ ^32^ ^]^ In vivo, FXR operates as a heterodimer with another nuclear receptor named RXR*α*.^[^ ^73^ ^]^ RXRs also act as specific heterodimer partners of many other nuclear receptors, including receptors for retinoic acid (RAR), thyroid hormone (TR), peroxisome proliferator activators (PPARs) and vitamin D.^[^ ^74^, ^75^ ^]^ A study indicated that the conformational conversion of the FXR/RXR*α* heterodimer may be utilized for future drug development targeting FXR.^[^ ^34^ ^]^ The co‐IP assay proved that FXR binds to RXR*α* in ADSC after FXR overexpression. RXR*α* gene silencing significantly blocked Angptl4 and Nqo‐1 expression induced by bile acid‐FXR axis activation, decreased ADSC retention after intramyocardial injection, abolished the proangiogenic effect of ADSC and decreased ADSC survival. Moreover, RXR*α* knockdown partially attenuated the cardioprotection of intramyocardially injected ADSC‐FXR. The ChIP assay and luciferase reporter assay revealed that FXR binds to the promoter regions of the Angptl4 and Nqo‐1 genes and directly regulates Anptl4 and Nqo‐1 transcription.

## 4 Conclusion

We demonstrated that the low expression of FXR in ADSC limits their cardioprotective effect. FXR overexpression failed to improve ADSC survival in vitro and exerted limited paracrine proangiogenic effects. Administration of OCA, a synthetic bile acid, significantly enhances the survival and paracrine proangiogenic effect of ADSC overexpressing FXR. By performing targeted metabolomics using ischemic heart tissue, we identified two endogenous bile acids, CA and DCA, which also enhance the survival and paracrine function of ADSC‐FXR. Based upon the endogenous bile acid pool in the myocardial ischemic microenvironment, the survival of intramyocardially injected ADSC overexpressing FXR was significantly increased, and these cells played a key role in promoting angiogenesis, ameliorating cardiomyocyte apoptosis, and improving post‐MI HF. We demonstrated for the first time that bile acid‐FXR axis activation improves ADSC survival by promoting Nqo‐1 expression and enhances ADSC paracrine angiogenesis by increasing Angptl4 expression and secretion. We revealed that FXR directly regulates the expression of Angptl4 and Nqo‐1 by forming a heterodimer with RXR*α*. Therefore, FXR overexpression in ADSC might be a novel strategy for improving the curative effect of MSC‐based therapy for MI, as this change facilitates the response of MSC to endogenous bile acid signals.

## 5 Experimental Section

An expanded Experimental Section is included in the Supporting Information.

All animal experiment protocols were approved by the Animal Care and Use Committee of the Fourth Military Medical University (FMMU) and strictly abided by the National Institutes of Health Guidelines on the Use of Laboratory Animals (NIH publication No. 85‐23, revised 2011).

### Statistical Analysis

All results were presented as the mean ± SEM. GraphPad Prism 7 software (GraphPad Software, USA) was used for data analysis. The Gehan–Breslow–Wilcoxon test was performed to analyze the survival rate. Student's *t*‐test was performed to analyze two unpaired groups, and one‐way ANOVA followed by the Bonferroni post hoc test was performed to analyze the multiple groups. Differences with *p* < 0.05 were considered statistically significant.

## Conflict of Interest

The authors declare no conflict of interest.

## Author Contributions

Y.X., X.X., and Y.G. contributed equally to this work. L.T. and W.Y. defined the research theme and revised the manuscript critically. Y.X., X.X., and Y.G. designed methods and experiments, carried out the laboratory experiments, and wrote the paper. C.L., X.X., F.Z., M.F., T.Q., C.L., G.H., L.P., S.W., L.Z., C.H., and R.L. collected and analyzed the data, and interpreted the results.

## Supporting information

Supporting Information

Supporting Table 1

## Data Availability

The data that support the findings of this study are available from the corresponding author upon reasonable request.
