# Supplementary material for: Mesenchymal Stromal Cells Overexpressing Farnesoid X Receptor Exert Cardioprotective Effects Against Acute Ischemic Heart Injury by Binding Endogenous Bile Acids
Source: Adv Sci (Weinh). 2022 Jul 3;9(24):2200431. doi: 10.1002/advs.202200431 (PMC9404394; doi:10.1002/advs.202200431)
Supplement: Supplementary file 1 — Supporting Information [file ADVS-9-2200431-s002.pdf]

SUPPLEMENTARY MATERIALS

Experimental Methods

Animal models

All animal experiment protocols were approved by the Animal Care and Use Committee of the Fourth Military Medical University (FMMU) and strictly abided by the National Institutes of Health Guidelines on the Use of Laboratory Animals (NIH publication No. 85-23, revised 2011). Adult male C57BL/6J mice (8-10 weeks of age) were purchased from the Laboratory Animal Center of the FMMU. Male farnesoid X receptor knockout (FXR KO) mice (8-10 weeks of age) were used. The generation, breeding, phenotypic characteristics, and genotyping approach of these mice have previously been described in detail [1]. To establish MI models, the mice were anesthetized by inhalation of 2% isoflurane, and the left anterior descending coronary artery was ligated as described in our previous study [2]. MI mice with left ventricular ejection fraction (LVEF)>40% on day 1 were excluded from the study. The mice were anesthetized using 1%–2% isoflurane, and the hearts were harvested. The heart cavities were perfused with cold phosphate-buffered saline (PBS) and transected along the lower edge of the ligation line. The dissected distal tissue was fixed in 4% paraformaldehyde for histological analysis.

Isolation and culture of adipose tissue-derived MSC

Adipose tissue-derived MSC (ADSC) were isolated from 6- to 8-week-old C57BL/6 mice as we previously described [3]. In brief, the mice were anesthetized by inhalation of 2% isoflurane, and the skin around the groin was disinfected with alcohol-soaked cotton balls. An inverted "Y" incision was made along the groin and midline of the abdomen to expose subcutaneous adipose tissue. Adipose tissue was removed from both sides along the spermatic vein, with care to avoid clipping the epididymis and surrounding blood vessels. After washing thoroughly with cold PBS, the adipose tissue was cut into small pieces approximately 0.8 cm in diameter. The tissue pieces were collected in type I collagenase solution (1 mg/ml) and digested in a 37°C water bath for approximately 1 hour. After complete digestion, the tissue was filtered through a 70-μm cell filter and centrifuged at 800 g for 10 min. After centrifugation, the supernatant was discarded. Red blood cell lysate (approximately 0.5 ml) and DMEM/F12 containing 10% fetal bovine serum (FBS) and 1% penicillin–streptomycin were added to the cell pellet, and the cells were resuspended and inoculated into a 100-mm culture dish. When ADSC reached 90% confluence, they were passaged by trypsin digestion. Cells from passage 3 (P3) were used in the following experiments.

Intramyocardial injection of ADSC

The detailed steps for intramyocardial injection of ADSC were described in our previous study [3]. In brief, P3 ADSC were used for our research. The ADSC were digested and resuspended in PBS containing 0.2 mmol/L EDTA. To trace ADSC in vivo, the lipophilic red fluorescent dye CM-DiI (5 μmol) was added to the cell suspension for 20 min in the dark (37°C). After dyeing, the ADSC were centrifuged and resuspended in PBS containing 0.2 mmol/L EDTA. Each group of ADSC was diluted to 2×10^7 ADSC/mL, and 10 μL was directly injected into the left ventricular free wall using a 30.5-G Hamilton syringe (Hamilton Co., Reno, NV, USA). When establishing the MI model, immediately after ligating the left anterior descending coronary artery, ADSC were injected into the infarcted border zone at 3 sites (3-4 μL per site,

2×10^5 cells/heart). After injection of ADSC, the heart was immediately placed back into the thoracic cavity, followed by manual evacuation of pneumothoraxes, closure of muscle, and skin suture.

FXR overexpression in ADSC by adenoviral vector transfection

To achieve FXR gene overexpression in ADSC, ADSC were transfected with adenoviral vectors (GenePharma Co., Ltd. Suzhou, China) containing FXR cDNA (Ad-FXR). To identify the most suitable multiplicity of infection (MOI), ADSC were transfected with different titers of adenovirus, including 5, 10, 25 and 50 pfu/cell (10 pfu/cell was the most suitable). The control adenovirus vector (Ad-con) was constructed by transfection with adenovirus lacking the FXR sequence. ADSC were incubated with adenovirus that was added to F12-DMEM culture medium containing 10% FBS (fresh medium; FM) for 8 hours. Then, the ADSC were washed with PBS before incubation in FM. Additional treatments were performed 48 hours after transfection.

Small interfering RNA (siRNA) transfection

The target genes were knocked down by using siRNA as we previously described [3]. In this experiment, all siRNAs for the target genes and the negative control siRNA (siRNA NC) were designed and provided by GenePharma Co., Ltd. (Suzhou, China). In brief, ADSC were seeded in 100-mm petri dishes or 6-well plates. When the ADSC reached 70-80% confluence, the siRNAs were applied for transfection using Lipofectamine™ RNAiMAX Transfection Reagent (#13778075, Invitrogen, USA) according to the manufacturer's instructions. ADSC were treated with siRNA for 24 hours and then transfected with Ad-FXR or Ad-con.

Treatment of ADSC and preparation of conditioned medium (CM)

After transfection with adenoviral vectors for 24 hours, ADSC were pretreated with/without obeticholic acid (OCA, 10 μM) or each bile acid (10 μM) in DMEM/F12 for another 24 hours. To induce oxidative stress in vitro, H_2O_2 (200 μM) was added to the above medium, and ADSC were incubated for another 24 hours. To obtain CM, the ADSC were washed with PBS after treatment with OCA and then incubated with DMEM/F12 without FBS and other supplements for another 24 hours. The medium was collected and centrifuged (3000 rpm, 5 min) to remove impurities, and CM was obtained for further experiments.

Determination of cardiac function

To determine the cardiac function of mice, a transthoracic echocardiography system (VisualSonics, Vevo 770) was used as we previously described [3]. In brief, the mice were placed in the supine position on a heating pad after being anesthetized with 1.5% isoflurane. The LVEF and the left ventricular internal diameter at the end of systole (LVIDs) and diastole (LVIDd) were measured under both the long axis and the short axis M-mode.

Determination of cardiomyocyte and injected ADSC apoptosis

Cardiomyocyte apoptosis was evaluated using terminal deoxynucleotidyl transferase dUTP nick-end labeling (TUNEL) staining with the *In Situ* Cell Death Detection Kit (Roche, 11684817910) according to the manufacturer's instructions. ADSC apoptosis was evaluated using a One Step TUNEL Apoptosis Assay Kit (Beyotime, C1090) according to the manufacturer's instructions. As we previously described, the assessed section of myocardial tissue includes the entire MI area [4].

Determination of angiogenesis

Angiogenesis was evaluated by calculating the microvessel density per field in the peri-infarct area as we previously described [3]. The anti-CD31 antibody (1:100, Cat. No. ab28364, Abcam) was used. Only microvessels with a clear lumen structure or linear blood vessel shape were counted. Branching vessel structures were not counted repeatedly.

Determination of capillary-like tube formation

Rat coronary artery endothelial cell (RCAEC) tube formation assays were performed to evaluate the tube formation capacity of endothelial cells as previously described [3]. In brief, Matrigel diluted in DMEM/F12 was laid on the bottom of a 96-well plate, which was then placed in a cell incubator for 40 min. Then, RCAEC resuspended in DMEM without FBS were seeded on the Matrigel at a density of $1 \times 10^4/cm^2$, and the plate was placed in a cell incubator for 4-6 hours. Images of tube formation were obtained with an optical microscope (Nikon, Japan).

Determination of cardiac fibrosis

To evaluate cardiac remodeling, heart tissue was cut into 5-μm-thick sections after fixation in formalin and embedding in paraffin. Masson's Trichrome Stain Kit (Solarbio, G1340) was used to assess myocardial fibrosis according to the manufacturer's instructions. After using an optical microscope (Nikon, Japan) to obtain images, the degree of interstitial fibrosis was analyzed by software (Image-Pro plus 6.0, Media Cybernetics).

Flow cytometric analysis of cultured ADSC apoptosis

The apoptosis of cultured ADSC was assessed with an annexin V-EGFP detection kit (BIOBOX, BA1250) according to the manufacturer's instructions. Briefly, after digestion with 0.25% trypsin and washing with PBS, ADSC were loaded with a saturating concentration of annexin V-EGFP for 10 min at 37°C, and apoptotic cells were counted by flow cytometry (Beckman Coulter, America) and analyzed by EXPO32 ADC.

Isolation of cell-derived exosomes

ADSC were seeded in 100 mm culture dishes (Invitrogen, California) and transfected with Ad-con or Ad-FXR for 24 hours, followed by incubation with vehicle or 10 μM OCA for another 24 hours. Exosomes were extracted from the CM of ADSC by using the Total Exosome Isolation Kit for Cell Culture Medium (Invitrogen, Lithuania) according to the manufacturer's instructions. Briefly, the obtained CM was centrifuged at 3000 g for 30 minutes and transferred to stevile tubes. Following the addition of the reagent to the CM and incubation at 4 °C overnight, the mixture was centrifuged at 4 °C and 10000 g for 1 hour. After the supernatant of the mixture was discarded, the exosomes attached to the tube were suspended in PBS and normalized by the number of cells (the cells they were isolated from) to ensure that 100 μL of PBS suspension contained exosomes isolated from 10^6 cell [5].

Transmission electron microscopy (TEM)

The morphology of the exosomes was evaluated using transmission electron microscopy (TEM) as we previously described [5]. Briefly, purified exosomes were fixed in 2% paraformaldehyde, negatively stained with 2% aqueous uranyl acetate solution, and then loaded on Formvar–carbon-coated grids. Images of the exosomes were obtained under a transmission electron microscope (JEOL JEM-1230, Tokyo Japan) at 80 kV.

Nanoparticle tracking analysis (NTA)

Exosomes from 10^7 cells were diluted in 1 mL PBS for nanoparticle tracking analysis. The size of isolated exosomes was determined although Nanosight Tracking Analysis by utilizing ZetaView PMX 110 (Particle Metrix, Meerbusch, Germany) according to our previous protocol [5].

Flow cytometric analysis for exosomes concentration

The exosome concentration was assessed with a NanoFCM system. By measuring the number of fluorescent exosomes with a calibrated concentration, the volume flow of the sample under a specific pressure was obtained, and then the standard curve of exosome concentration was generated. Under the same sampling conditions, the exosome concentration of the sample to be tested can be obtained according to the standard curve.

Quantitative RT-PCR

The protocols for the extraction and transcription of total RNA were described in our previous study [4]. In brief, RNA was isolated from cultured cells with the MiniBEST Universal RNA Extraction Kit (#9767, Takara). RNA quality and concentration were tested using a SpectraMax QuickDrop Micro-Volume Spectrophotometer (Molecular Devices). cDNA was generated from RNA by using the PrimeScript™ RT reagent Kit with gDNA Eraser (DRR047A, Takara). The expression of each gene was determined in triplicate samples with a PCR detection kit (DRR081A, TaKaRa) and CFX96 system (Bio-Rad). The primers used in this study were designed and provided by TSINGKE Biotech. All primer sequences are shown in Supplementary Table 1.

tdTomato DNA determination by quantitative PCR

The number of cells engrafted to the heart 6 hours, 1 day, 3 days and 7 days after transplantation was determined via quantitative PCR assessments of tdTomato DNA levels as we previously described, with some modificatio [6]. Genomic DNA was prepared from tdTomato-ADSC and normal heart tissues via a Genomic DNA Extraction Kit (TIANGEN, W9426). The exogenous tdTomato gene was amplified to calculate the number of tdTomato-ADSC. Total mouse heart tissue genomic DNA was identified by GAPDH. Purified DNA (300 ng) was amplified in triplicate in 20 µl reactions via SYBR Green PCR Master Mix (Thermo Fisher Scientific, 4472908). Amplification was performed with tdTomato forward (5'-CTCCGAGGACAACAACATGG-3'), tdTomato reverse (5'-CTTGTACAGCTCGTCCATGC-3'), GAPDH forward (5'-GGTGAAGGTCGGTGTGAACG-3'), and GAPDH reverse (5'-CTCGCTCCTGGAAGATGGTG-3') primers. The PCR procedure was as follows: 95°C for 5 minutes, 1 cycle; 95°C for 10 seconds, 55°C for 20 seconds, 72°C for 20 seconds, 40 cycles. After amplification, DNA melting curves were generated by denaturing at 95°C for 15 seconds, cooling to 65°C for 1 minute, and then increasing the temperature to 0.5°C/s until reaching 95°C while continuously monitoring fluorescence. Standard curves of tdTomato and GAPDH genes generated (by diluting the genomic DNA of tdTomato-ADSCs in normal heart tissue genomic DNA at a ratio from 1:1 to 1:1,000) were used as a reference for unknown DNA samples. Cell engraftment was quantified as the number of tdTomato-positive cells per 100 heart cells in the apex region.

LC-MS/MS analysis

After obtaining the CM of each group, four volumes of lysis buffer (8 M urea, 1% protease inhibitor cocktail) were added to the CM. The protein concentration was measured with a BCA kit according to the manufacturer's instructions. Dithiothreitol was added to the protein solution to a final concentration of 5 mM, and the mixture was reacted at 56°C for 30 min. Then, iodoacetamide was added to a final concentration of 11 mM, and the mixture was reacted for 15 min at room temperature in darkness. Finally, the urea concentration of the sample was diluted to less than 2 M. Trypsin was added in a 1:50 mass ratio (trypsin:protein) for the first digestion overnight, after which trypsin was added at a mass ratio of 1:100 (trypsin:protein) for a second 4 h digestion. The tryptic peptides were dissolved in 0.1% formic acid (solvent A), and they were directly loaded onto a homemade reversed-phase analytical column (15-cm length, 75 μm i.d.). The gradient included an increase from 6% to 23% solvent B (0.1% formic acid in 98% acetonitrile) over 26 minutes, an increase from 23% to 35% over 8 minutes, climbing to 80% over 3 minutes, and then holding at 80% for the last 3 minutes. The above steps were performed on the EASY-nLC 1000 UPLC system at a constant flow rate of 400 nL/min. The peptides were passed through an NSI source and then subjected to tandem mass spectrometry (MS/MS) in a Q Exactive^{TM} Plus (Thermo) coupled online to the UPLC. The applied electrospray voltage was 2.0 kV. The m/z scanning range of the full scan was 350 to 1800, and the peptides were detected in the Orbitrap with a resolution of 70000. The NCE setting of 28 was used to select peptides for MS/MS, and the Orbitrap at a resolution of 17500 was used to detect the fragments. The data-dependent procedure alternated between one MS scan and the following 20 MS/MS scans, with a dynamic rejection time of 15 seconds. The automatic gain control (AGC) was set at 5E4. The resulting MS/MS data were processed by the MaxQuant search engine (v1.5.2.8).

Protein extraction and western blotting analysis

The protocols for the extraction of total protein from cultured cells or exosomes and western blotting were described specifically in our previous study [4]. The proteins were separated on SDS–PAGE gels (10% for FXR, Angptl4, Nqo-1, RXRα, calnexin and CD63; 12% for cleaved caspase-3 and cleaved caspase-3). Then, the proteins were transferred to a polyvinylidene fluoride membrane (Millipore). After blocking in 5% milk for 2 hours at room temperature, the membranes were washed three times with PBS and incubated with primary antibodies against FXR (Invitrogen #A9033A, 1:1000), cleaved caspase-3 (CST #9661, 1:1000), caspase-3 (CST #9662, 1:1000), Angptl4 (Affinity #DF6751, 1:1000), Nqo-1 (Abcam #ab80588, 1:1000), HO-1 (Abcam #ab189491, 1:1000), SOD2 (CST #13141, 1:1000), SOD3 (NOVUS #4GG11G6, 1:1000), CD 63 (Sangon Biotech #D360973, 1:1000), calnexin (ABclonal #A4846, 1:1000), β-actin (Affinity #T0022, 1:5000), β-tubulin (Proteintech #66240-1-lg, 1:5000), gapdh (Proteintech #10494-1-AP, 1:5000), and RXRα (Abcam #ab125001, 1:1000). After incubation with the primary antibodies overnight at 4°C, the membranes were washed three times with PBS and incubated with secondary HRP-conjugated anti-rabbit antibody (BioCytoSci SA-10011, 1:5000) or anti-mouse antibody (BioCytoSci 223 #SA-10010, 1:5000) for 1 hour at room temperature. The bands were detected with an enhanced chemiluminescence kit (Millipore, WBKLS0100) and quantified with Quantity One software (Bio-Rad).

Neonatal rat cardiomyocyte isolation and culture

Neonatal rat ventricular myocytes (NRVM) were isolated from 1- to 2-day-old Sprague-Dawley rats as we previously described [4]. After isolation, the NRVM were cultured in DMEM containing 10% FBS, 1%

penicillin-streptomycin and 2 mM L-glutamine. To induce oxidative stress in vitro, NRVM were cultured in DMEM with H_2O_2 (200 μM) for 8 hours.

RNA sequencing (RNA-seq) analysis

Differential gene expression analysis was performed using RNA-seq at Shenzhen BGI Technology. After treatment with vehicle, GW4064 or Ad-FXR for 24 hours, total RNA was extracted from ADSC via TRIzol (Invitrogen, Carlsbad, CA, USA) according to the manufacturer's instructions. Sequencing libraries were constructed as follows: purified mRNA was fragmented into small pieces. First-strand cDNA was generated using random hexamer-primed reverse transcription, and then second-strand cDNA was synthesized. A-tailing mix and RNA index adaptors were added for end repair. After amplification by PCR, the final double-stranded PCR products were heated, denatured and circularized by the splint oligo sequence to obtain the final library (single-stranded circular DNA, ssCir DNA). These ssCir DNAs were amplified with phi29 to make DNA nanoballs (DNBs). DNBs were loaded into the patterned nanoarray, and single-end 50-base reads were generated on the BGISEQ-500 platform (BGI-Shenzhen, China).

Differential expression analysis was performed using DESeq2 (v1.4.5). DESeq2 provides statistical methods for determining differential expression from digital gene expression data using a model based on the negative binomial distribution. The resulting P values were adjusted using Benjamini and Hochberg's approach for controlling the false discovery rate. When the GW4064 group was compared to the control group, genes with an adjusted P value <0.001 found by DESeq2 were assigned as differentially expressed. When the Ad-FXR group was compared to the control group, genes with an adjusted P value <0.05 found by DESeq2 were considered differentially expressed.

Bile acids analysis

Heart tissue samples were collected, and each sample was precisely weighed to the same mass of 25 mg. After the addition of 1000 μL of extract solution (precooled at -40°C, acetonitrile-methanol-water, 2:2:1, containing 0.1% formic acid and isotopically labeled internal standard mixture), the samples were vortexed (30 s), homogenized (35 Hz, 4 min), and sonicated (5 min) three times in an ice-water bath. The samples were incubated for 1 hour at -40°C and then centrifuged for 15 min (12000 rpm, 4°C) to obtain the supernatants. The supernatants were transferred to LC-MS vials for UHPLC-MS/MS analysis. The bile acid concentrations in the heart tissue were measured by a UHPLC system (Vanquish, Thermo Fisher Scientific) equipped with a Waters Acquity BEH C18 column (150 * 2.1 mm, 1.7 μm, Waters). Mobile phase An included a mixture of 0.1% acetic acid and 1 mmol/L ammonium acetate in water, and mobile phase B was acetonitrile. The column temperature was set at 50°C. The autosampler temperature was set at 4°C, and the injection volume was 1 μL. MS detection was determined by a Q Exactive HFX mass spectrometer (Thermo Fisher Scientific) in parallel reaction monitoring (PRM) mode. The different bile acid metabolites detected by LC-MS were identified by each standard.

Chromatin immunoprecipitation (ChIP) assay

ChIP assays were performed using a Simple ChIP Plus Enzymatic Chromatin IP Kit (Cell Signaling Technology, #9003, Danvers, USA) according to the manufacturer's instructions. Briefly, ADSC were fixed with formaldehyde, and the chromatin was sheared. After that, the fragmented chromatin was incubated with an FXR antibody (Invitrogen, #A9033A) and protein G magnetic beads. PCR was performed to analyze DNA

released from the precipitates. The binding sites of FXR to the promoter regions of the Angptl4 and NQO-1 genes were predicted by the JASPAR database. For Angptl4, four possible binding sites were predicted, and four pairs of specific primers were designed accordingly. ChIP and PCR analysis revealed that FXR bound to one of the predicted binding sites of the Angptl4 promoter region in ADSC. The primer sequences specific to the FXR binding region within the Angptl4 promoter region were as follows: forward: 5'-gtgtgcatgtgtgtgagtgc-3' and reverse: 5'-agccaggaggaactggaaat-3'. For Nqo-1, three possible binding sites were predicted and three pairs of specific primers were designed accordingly. The results revealed that FXR bound to one of the predicted binding sites of the Nqo-1 promoter region in ADSC. The primers specific to the Nqo-1 promoter binding region were as follows: forward: 5'-agccagggctacacagagaa-3' and reverse: 5'-gctgcgttctgctcttagaaa-3'.

Co-immunoprecipitation

The protocols for the co-immunoprecipitation were described specifically in our previous study [6]. Briefly, P2 ADSC were transfected with Ad-con or Ad-FXR for 2 days. Cells were washed twice with cold PBS, and lysed with cold lysis buffer (CST #9803) supplemented with 1 mg/mL DTBP (Thermo Fisher Scientific, 20665). The mixture was then incubated with anti-RXRα antibody (Abcam #ab125001) and protein A plus ultralink resin (Thermo Fisher Scientific, 53142) and rocked overnight at 4 °C. The protein A beads were washed extensively with lysis buffer. Proteins were eluted from the beads and resolved by IgG elusion buffer (Thermo Fisher Scientific, 1856202). Proteins were immunoblotted with anti-FXR mouse monoclonal antibody (Invitrogen, #A9033A, 1:1000) as described above.

Luciferase reporter assay

A dual-luciferase reporter assay was performed to determine whether FXR directly regulates Angptl4 and Nqo-1 transcription. The mouse full-length Angptl4 or Nqo-1 promoter (-2000-0) was cloned into the PGL3.0 Basic plasmid upstream of the luciferase reporter gene (PGL-FL-Angptl4 promoter reporter or PGL-FL-Nqo-1 promoter reporter). All plasmids were provided by Tsingke Biotechnology (Beijing, China). HEK-293T cells were transfected with the PGL-FL-Angptl4 or PGL-FL-Nqo-1 promoter constructs, and co-transfected with the Renilla luciferase reporter plasmid. Meanwhile, the cells were co-infected with Ad-FXR or Ad-con. A dual-luciferase reporter assay system (Promega, Madison, Wisconsin) was used to measure the firefly and Renilla luciferase activity of the harvested cells according to the manufacturer's instructions. The luciferase activity was measured and calculated using a GloMax96 plate reader (BioTek, USA).

Supplementary References

[1] S. Seok, H. Sun, Y. C. Kim, B. Kemper, J. K. Kemper, *DIABETES* **2021**, *70*, 733.

[2] E. Gao, Y. H. Lei, X. Shang, Z. M. Huang, L. Zuo, M. Boucher, Q. Fan, J. K. Chuprun, X. L. Ma, W. J. Koch, *CIRC RES* **2010**, *107*, 1445.

[3] W. Yan, C. Lin, Y. Guo, Y. Chen, Du Y, W. B. Lau, Y. Xia, F. Zhang, R. Su, E. Gao, Y. Wang, C. Li, R. Liu, X. L. Ma, L. Tao, *CIRC RES* **2020**, *126*, 857.

[4] Y. Xia, F. Zhang, S. Zhao, Y. Li, X. Chen, E. Gao, X. Xu, Z. Xiong, X. Zhang, J. Zhang, H. Zhao, W. Wang, H. Wang, Y. Guo, Y. Liu, C. Li, S. Wang, L. Zhang, W. Yan, L. Tao, *CARDIOVASC RES* **2018**, *114*, 1335.

[5] X. Y. Xu, B. M. Tian, Y. Xia, Y. L. Xia, X. Li, H. Zhou, Y. Z. Tan, F. M. Chen, *Stem Cells Transl Med* **2020**, *9*, 1414.

[6] W. Yan, Y. Guo, L. Tao, W. B. Lau, L. Gan, Z. Yan, R. Guo, E. Gao, G. W. Wong, W. L. Koch, Y. Wang, X. L. Ma, *CIRCULATION* **2017**, *136*, 2162.

Online Figures and Figure Legends

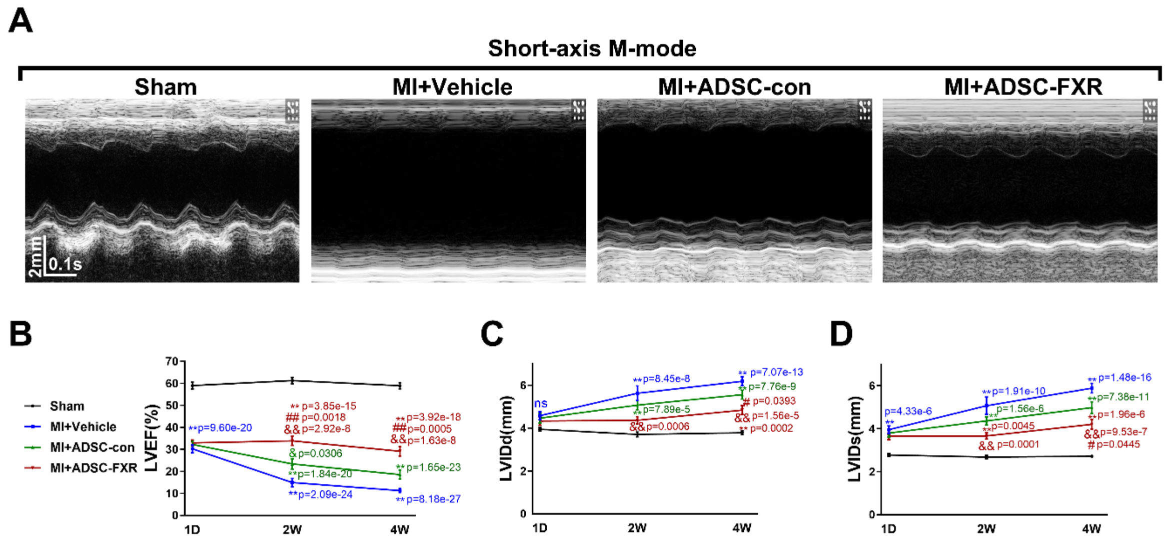

Online Figure 1. Intramyocardial injection of ADSC overexpressing FXR protected the heart against post-MI cardiac dysfunction.

(A) Cardiac function was evaluated by short-axis M-mode echocardiography 4 weeks after MI, and representative images are shown.

(B-D) LVEF, LVIDd and LVIDs were evaluated by short-axis M-mode echocardiography.

(A-D) n=20, 18, 19, 19 at 1 D; n=20, 14, 15, 18 at 2 W and 4 W. *p and **p versus Sham, $^{\&}$p and $^{\&\&}$p versus MI+vehicle, $^{\#}$p and $^{\#\#}$p versus MI+ADSC-con. 1D means 1 day after MI surgery. 2 W means 2 weeks after MI surgery. 4 W means 4 weeks after MI surgery.

Abbreviations: ADSC-con: ADSC transfected with adenovirus control for 2 days. ADSC-FXR: ADSC transfected with adenovirus-FXR for 2 days.

All data were analyzed using one-way ANOVA, followed by Bonferroni post hoc test. Data are presented as the mean ± SEM. *p < 0.05, **p < 0.01. ns: not significant.

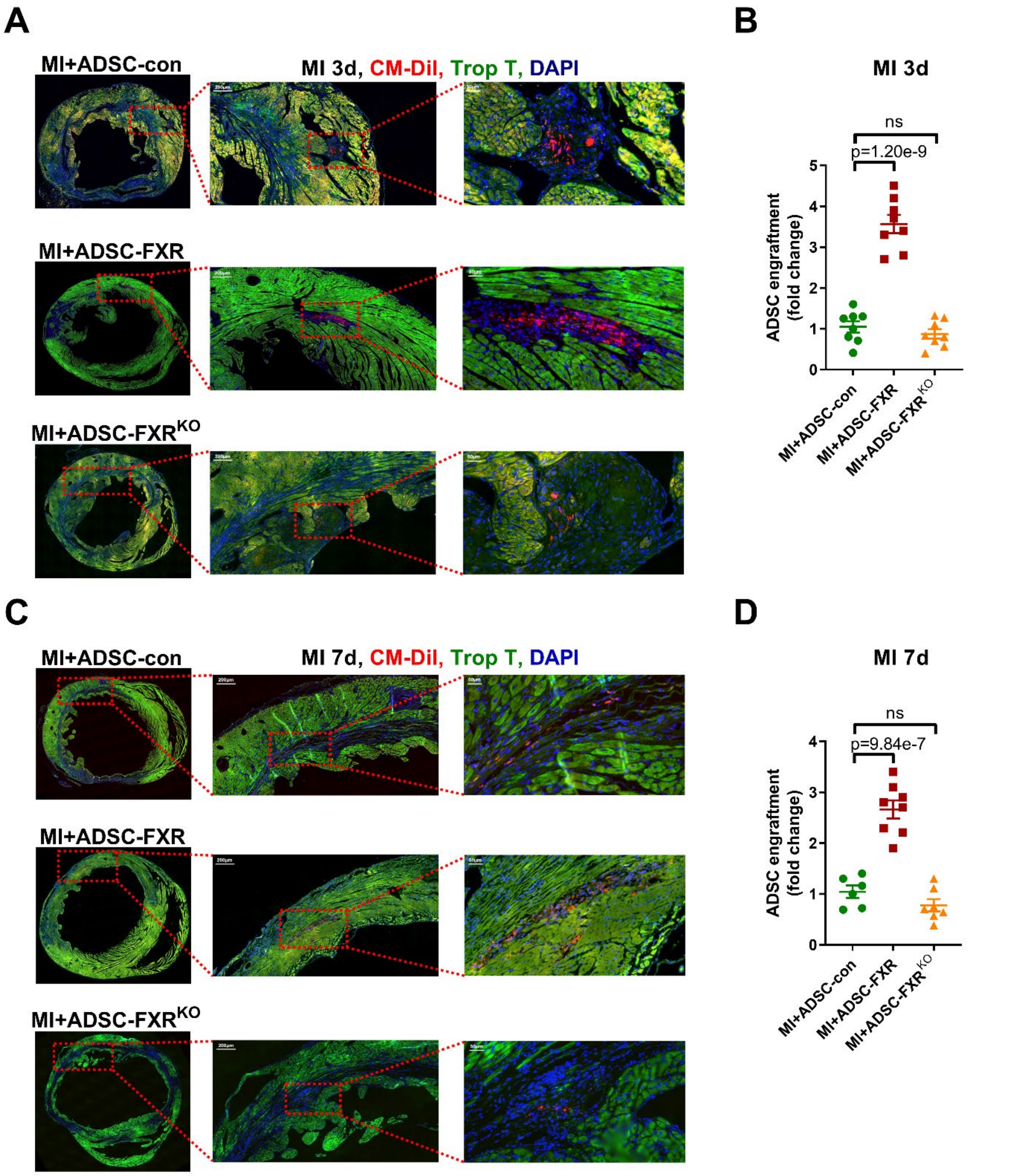

Online Figure 2. FXR overexpression increased the ADSC retention rate in vivo.

(A) The ADSC retention rate was determined by quantifying CM-DiI-labeled ADSC. Representative images of CM-DiI-labeled ADSC (red) in the ischemic heart 3 days after intramyocardial injection are shown. The heart tissue sections were immunofluorescence stained with troponin T (Trop T, green) and DAPI (blue).

(B) Quantification of CM-DiI-labeled ADSC was analyzed by the percentage of red area/infarcted area (Trop T negative area) 3 days after intramyocardial injection. (n=8, 8, 8).

(C) Representative images of CM-DiI-labeled ADSC in the ischemic heart 7 days after intramyocardial injection are shown.

(D) Quantification of CM-DiI-labeled ADSC 7 days after intramyocardial injection. (n=6, 8, 7). Abbreviations: ADSC-con: ADSC transfected with adenovirus-control for 2 days. ADSC-FXR: ADSC transfected with adenovirus-FXR for 2 days. ADSC-FXR KO: ADSC isolated from FXR knockout mice. All data were analyzed using one-way ANOVA, followed by Bonferroni post hoc test. Data are presented as the mean ± SEM. ns: not significant.

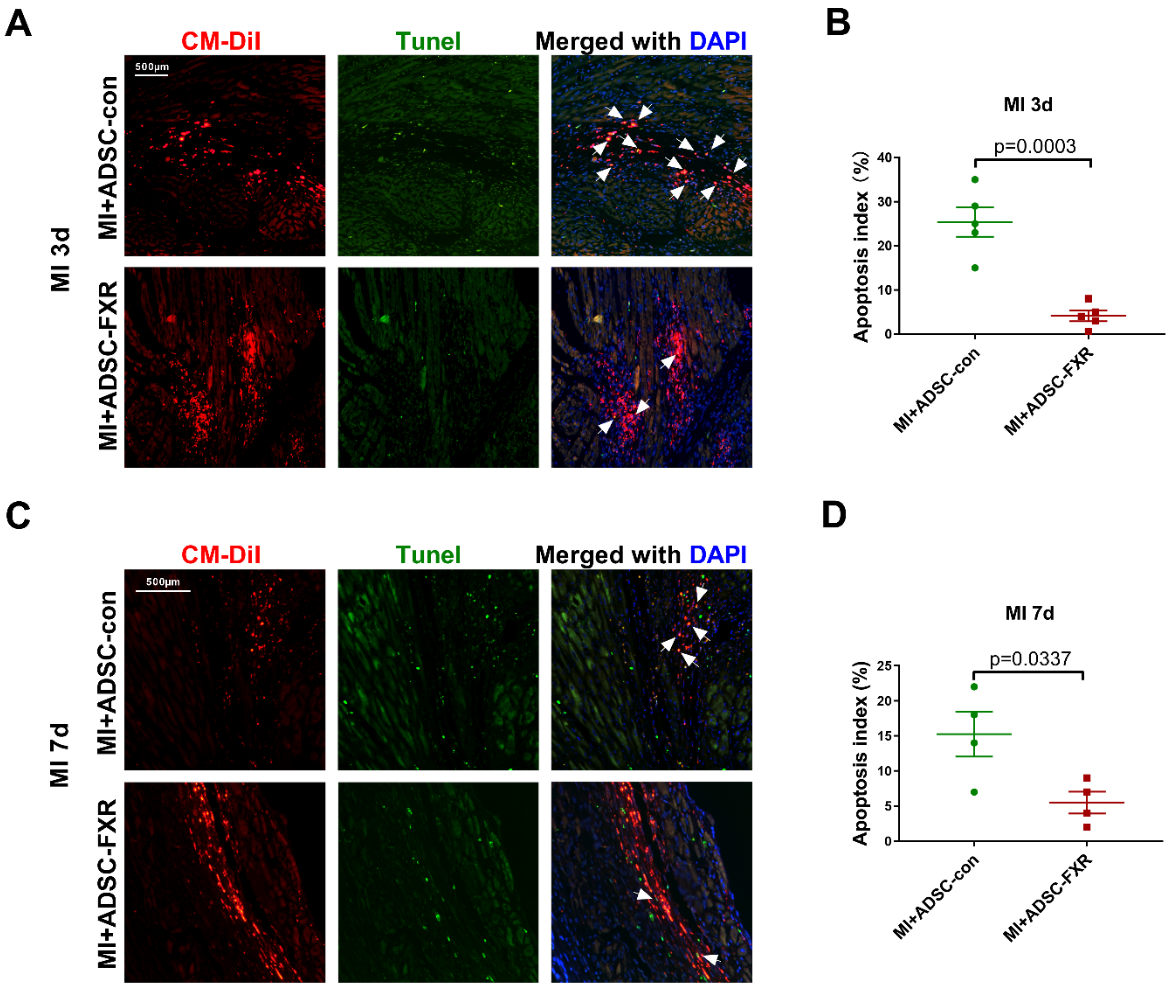

Online Figure 3. FXR overexpression decreased the ADSC apoptosis rate in vivo.

(A) ADSC apoptosis at 3 days after intramyocardial injection was determined by TUNEL staining (green) in the peri-infarct area, and representative images of CM-DiI-labeled ADSC (red) are shown. The nuclei were stained by DAPI (blue).

(B) Quantification of ADSC apoptosis (the percentage of TUNEL-positive nuclei/CM-DiI-positive nuclei) in the peri-infarct area 7 days after MI (n=5 per group).

(C) ADSC apoptosis at 7 days after intramyocardial injection was determined by TUNEL staining (green) in the peri-infarct area, and representative images of CM-DiI-labeled ADSC (red) are shown. The nuclei were stained by DAPI (blue).

(D) Quantification of ADSC apoptosis (the percentage of TUNEL-positive nuclei/CM-DiI-positive nuclei) in the peri-infarct area 7 days after MI (n=4 per group).

Abbreviations: ADSC-con: ADSC transfected with adenovirus control for 2 days. ADSC-FXR: ADSC transfected with adenovirus-FXR for 2 days.

All data were analyzed using unpaired Student's t test. Data are presented as the mean ± SEM.

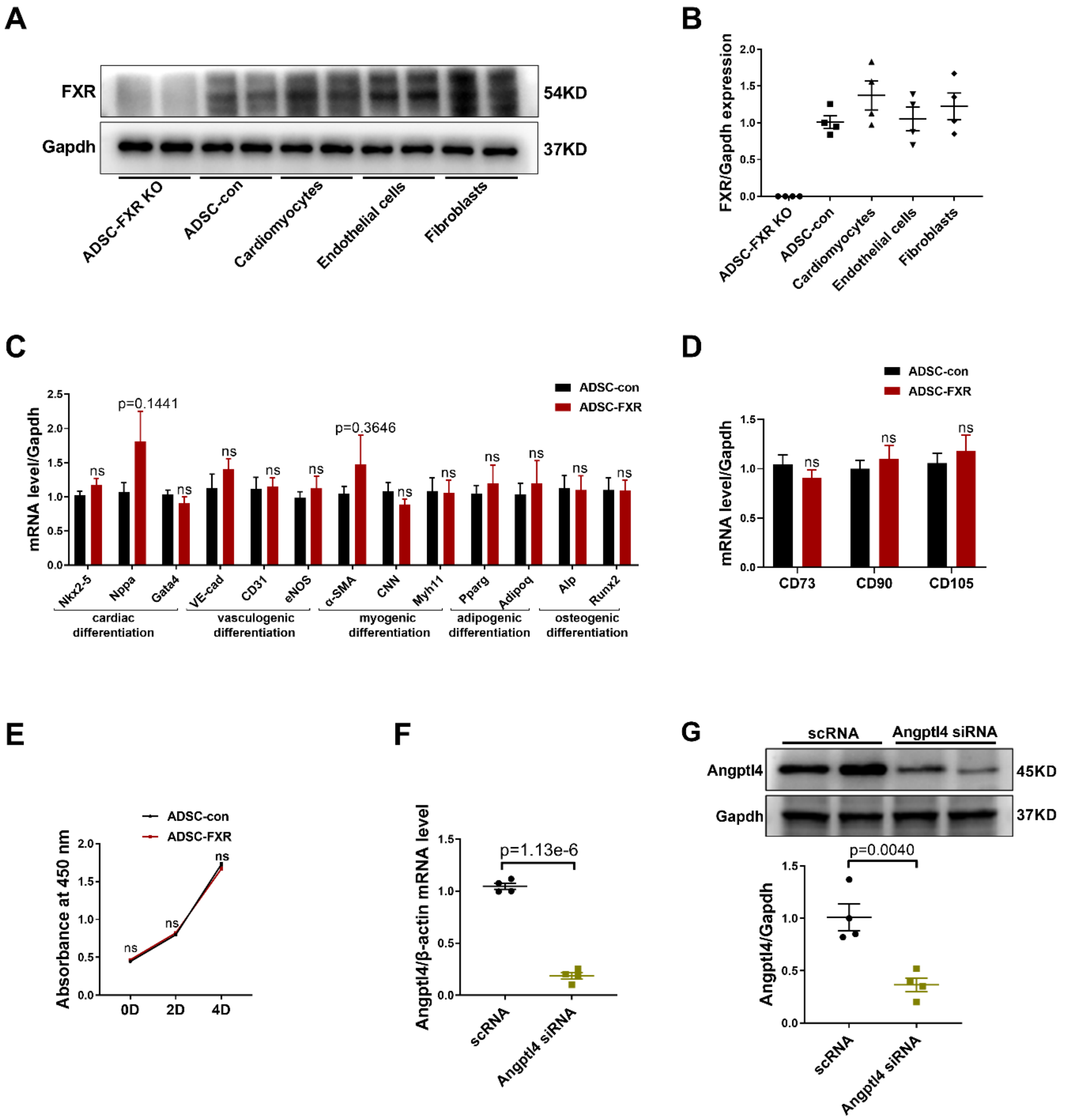

Online Figure 4. Basal FXR expression was low but not absent in ADSC, and FXR overexpression did not affect the surface marker expression, differentiation capacity or proliferation capacity of ADSC.

(A-B) Representative western blotting images and quantification of FXR protein expression in ADSC from FXR knockout mice (ADSC-FXR KO) and ADSC from control mice (ADSC-con), cardiomyocytes, endothelial cells and fibroblasts from control mice (n=4 per group).

(C) Genes related to cell differentiation mRNA levels were determined by RT-PCR (n=5).

(D) The mRNA levels of mesenchymal stem cell surface markers were determined by RT-PCR (n=6). Abbreviations: ADSC-con: ADSC transfected with adenovirus control for 7 days. ADSC-FXR: ADSC transfected with adenovirus-FXR for 7 days.

(E) The proliferative activity of ADSC was determined by the CCK-8 assay (n=15 per group). Abbreviations: ADSC-con: ADSC transfected with adenovirus control for 4 days. ADSC-FXR: ADSC transfected with adenovirus-FXR for 4 days.

(F) Angptl4 mRNA levels were determined by RT-PCR (n=4).

(G) Representative western blotting images and quantification of Angptl4 protein expression in ADSC (n=4 per group).

Data in C-G were analyzed using unpaired, 2-tailed Student's t test. Data are presented as the mean ± SEM. ns: not significant.

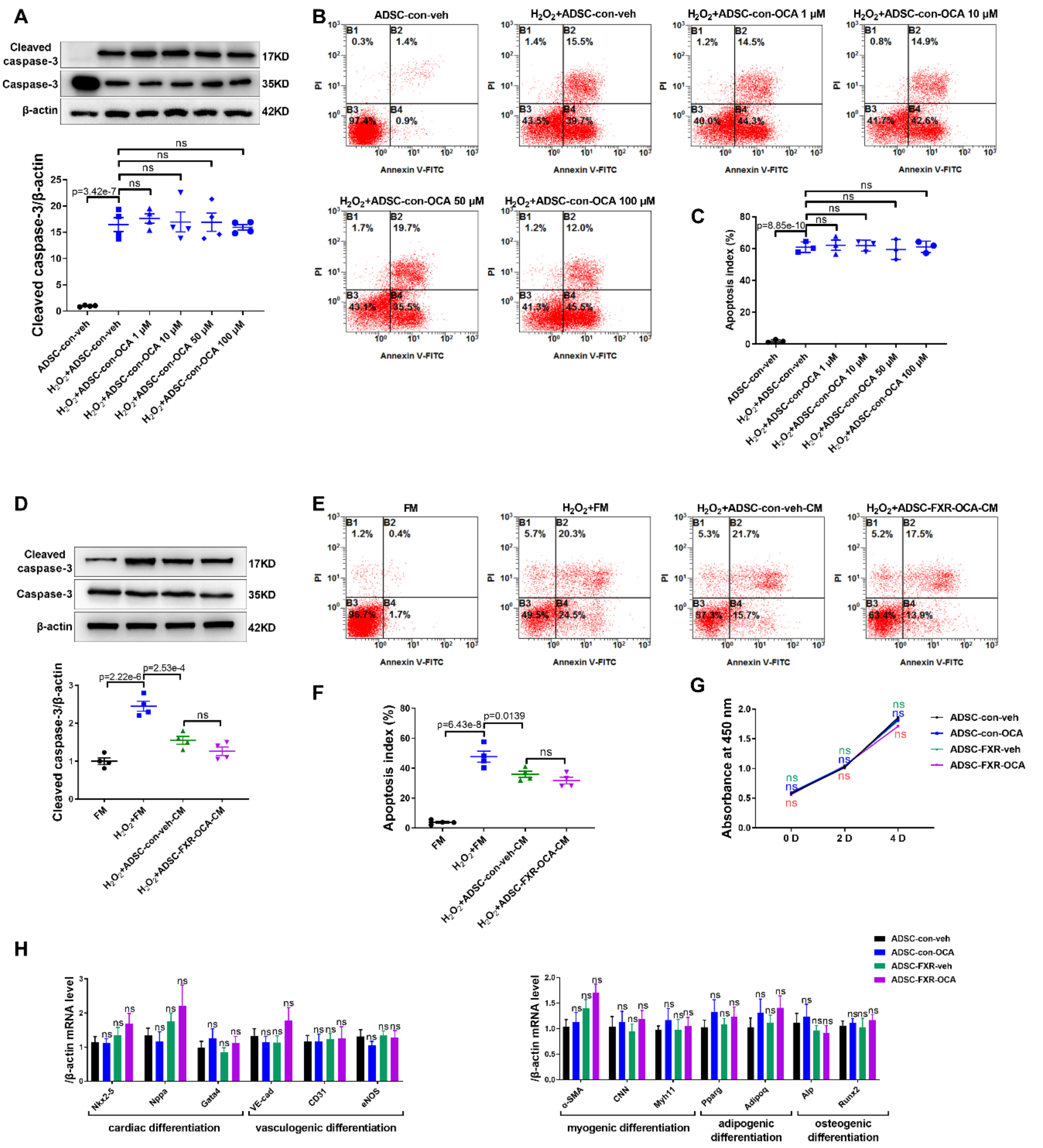

Online Figure 5. OCA administration without FXR overexpression did not improve ADSC survival, and FXR overexpression with OCA administration did not enhance the proangiogenic ability and differentiation capacity of ADSC.

(A) Representative western blotting images and quantification of the protein expression of cleaved caspase-3 and caspase-3 in ADSC. ADSC-con-veh and ADSC-con-OCA were treated with H_2O_2 (200 μM) for 24 hours (n=4 per group).

(B) Flow cytometric analysis of ADSC-con-veh and ADSC-con-OCA treated with H_2O_2 (200 μM) for 24 hours, and representative images are shown.

(C) ADSC apoptosis was quantified as the sum of the proportion of cells in quadrants B2 and B4 (n=3 per group).

(D) Representative western blotting images and quantification of the protein expression of cleaved caspase-3 and caspase-3 in NRVM. NRVM were incubated with FM, CM of ADSC-con-veh (ADSC-con-veh-CM), or CM of ADSC-FXR-OCA (ADSC-FXR-OCA-CM) for 24 hours followed by H_2O_2 (200 μM) treatment for another 8 hours (n=4 per group).
(E) Flow cytometric analysis of NRVM after incubation with FM, ADSC-con-veh-CM or ADSC-FXR-OCA-CM for 24 hours followed by H_2O_2 (200 μM) treatment for another 8 hours, and representative images are shown.
(F) ADSC apoptosis was quantified as the sum of the proportion of cells in quadrants B2 and B4 (n=4 per group).
Abbreviations: OCA: obeticholic acid. ADSC-con-veh: ADSC transfected with adenovirus control for 24 hours, followed by incubation with vehicle for another 24 hours. ADSC-FXR-OCA: ADSC transfected with adenovirus-FXR for 24 hours, followed by incubation with 10 μM OCA for another 24 hours. FM: fresh medium. CM: conditioned medium. NRVM: neonatal rat ventricular myocytes.
(G) The proliferative activity of ADSC was determined by the CCK-8 assay (n=15 per group). Abbreviations: ADSC-con-veh: ADSC transfected with adenovirus control for 24 hours, followed by incubation with vehicle for another 3 days. ADSC-FXR-veh: ADSC transfected with adenovirus-FXR for 24 hours, followed by incubation with vehicle for another 3 days. ADSC-con-OCA: ADSC transfected with adenovirus control for 24 hours, followed by incubation with 10 μM OCA for another 3 days. ADSC-FXR-OCA: ADSC transfected with adenovirus-FXR for 24 hours, followed by incubation with 10 μM OCA for another 3 days.
(H) Genes related to cell differentiation mRNA levels were determined by RT-PCR (n=5 per group). Abbreviations: ADSC-con-veh: ADSC transfected with adenovirus control for 24 hours, followed by incubation with vehicle for another 6 days. ADSC-FXR-veh: ADSC transfected with adenovirus-FXR for 24 hours, followed by incubation with vehicle for another 6 days. ADSC-con-OCA: ADSC transfected with adenovirus control for 24 hours, followed by incubation with 10 μM OCA for another 6 days. ADSC-FXR-OCA: ADSC transfected with adenovirus-FXR for 24 hours, followed by incubation with 10 μM OCA for another 6 days.
All data were analyzed using one-way ANOVA, followed by Bonferroni post hoc test. Data are presented as the mean ± SEM. ns: not significant.

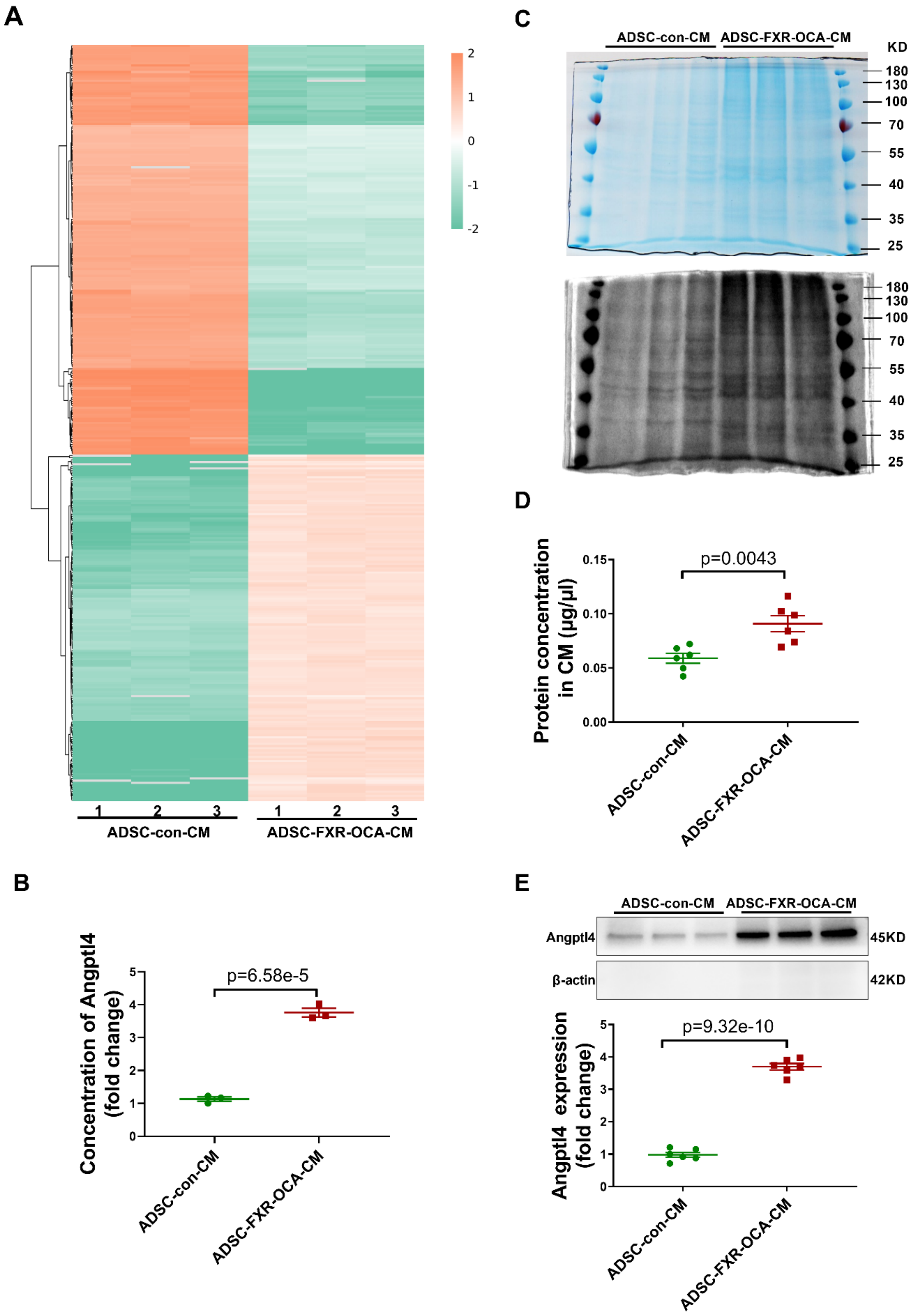

Online Figure 6. Bile acid-FXR axis activation promoted Angptl4 expression and secretion.

(A) The differential clustering heatmap between samples from ADSC-con-CM and ADSC-FXR-OCA-CM by LC-MS/MS (n=3 per group, fold change > 3, p < 0.0001).

(B) Quantification of Angptl4 concentration in CM (n=3 per group).

(C) Colloidal blue staining gel and image demonstrating the total amounts of protein between the two groups (n=3 per group).

(D) Total protein concentration in CM measured by Bio-Rad Protein Assay (n=6 per group).

(E) Representative western blotting images and quantification of Angptl4 protein expression in CM (n=6 per group).

Abbreviations: ADSC-con: ADSC transfected with adenovirus control for 2 days. ADSC-FXR-OCA: ADSC transfected with adenovirus-FXR for 24 hours, followed by incubation with 10 μM OCA for another 24 hours. FM: fresh medium. CM: conditioned medium.

All data were analyzed using unpaired Student's t test. Data are presented as the mean ± SEM.

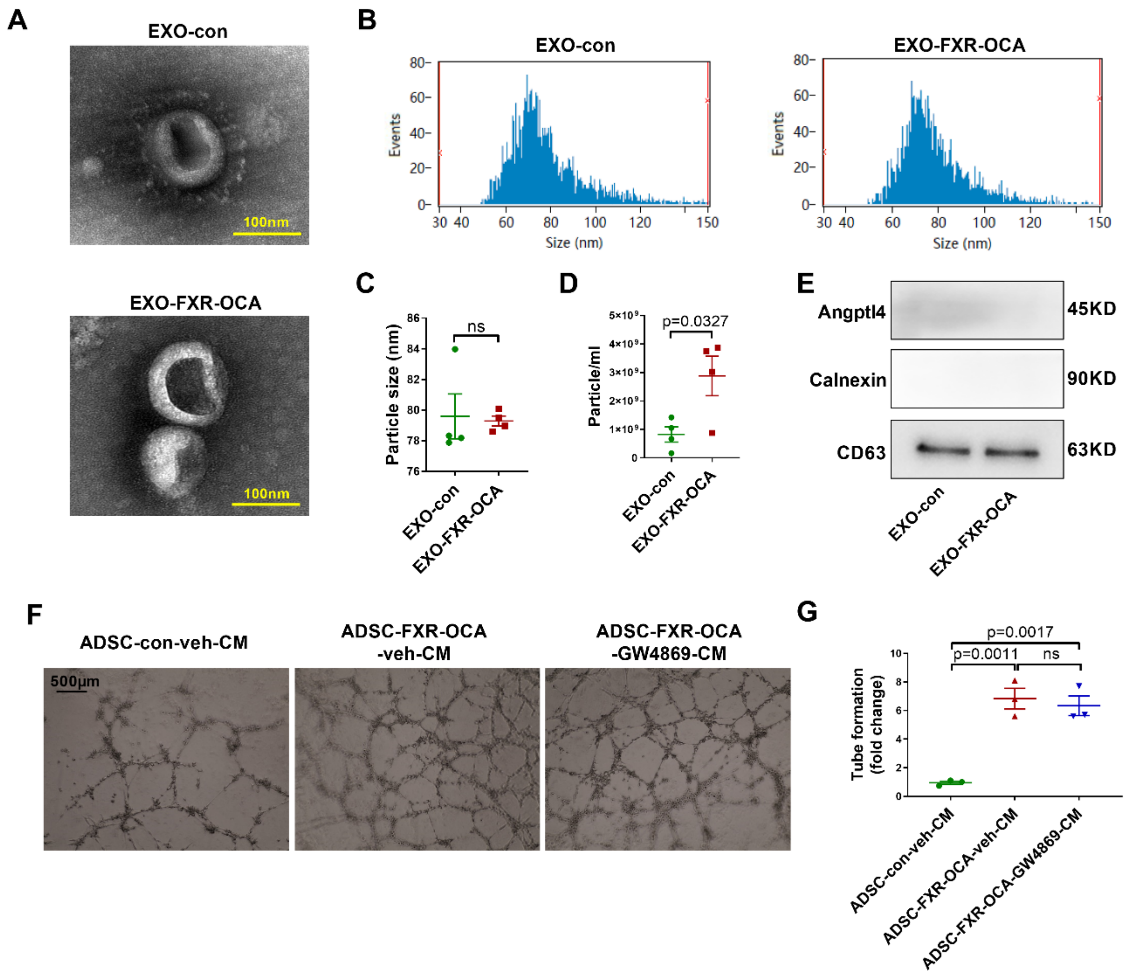

Online Figure 7. Exosome inhibitors failed to impair the proangiogenic effect of ADSC with bile acid-FXR axis activation.

(A) Representative TEM images of EXO-con and EXO-FXR-OCA (scale bar=100 nm).

(B) Size distribution profile of EXO-con and EXO-FXR-OCA as determined by nanoparticle tracking analysis.

(C) Quantification of the particle size of EXO-con and EXO-FXR-OCA (n=4 per group).

(D) Quantification of the particle concentrations of EXO-con and EXO-FXR-OCA (n=4 per group).

(E) Representative western blotting images of the protein expression of Angptl4, calnexin, and CD63 in EXO-con and EXO-FXR-OCA.

Abbreviations: EXO-con: exosomes extracted from control ADSC. EXO-FXR-OCA: exosomes extracted from ADSC transfected with adenovirus-FXR for 24 hours, followed by incubation with 10 μM OCA for another 24 hours.

(F) A tube formation assay of RCAEC was performed to evaluate the proangiogenic capability of ADSC-con-veh, ADSC-FXR-OCA-veh and ADSC-FXR-OCA-GW4869, and representative images are shown.

(G) Quantification of tube formation (n=3 per group).

Abbreviations: ADSC-con-veh: ADSC transfected with adenovirus control for 24 hours, followed by incubation with vehicle for another 24 hours. ADSC-FXR-OCA-veh: ADSC transfected with adenovirus-

FXR for 24 hours, followed by incubation with 10 μM OCA for another 24 hours. ADSC-FXR-OCA-GW4869: ADSC transfected with adenovirus-FXR for 24 hours, followed by incubation with 10 μM OCA and GW4869 for another 24 hours. FM: fresh medium. CM: conditioned medium.

Data in H were analyzed using one-way ANOVA, followed by Bonferroni post hoc test. Other data were analyzed using unpaired Student's t test. Data are presented as the mean ± SEM. ns: not significant.

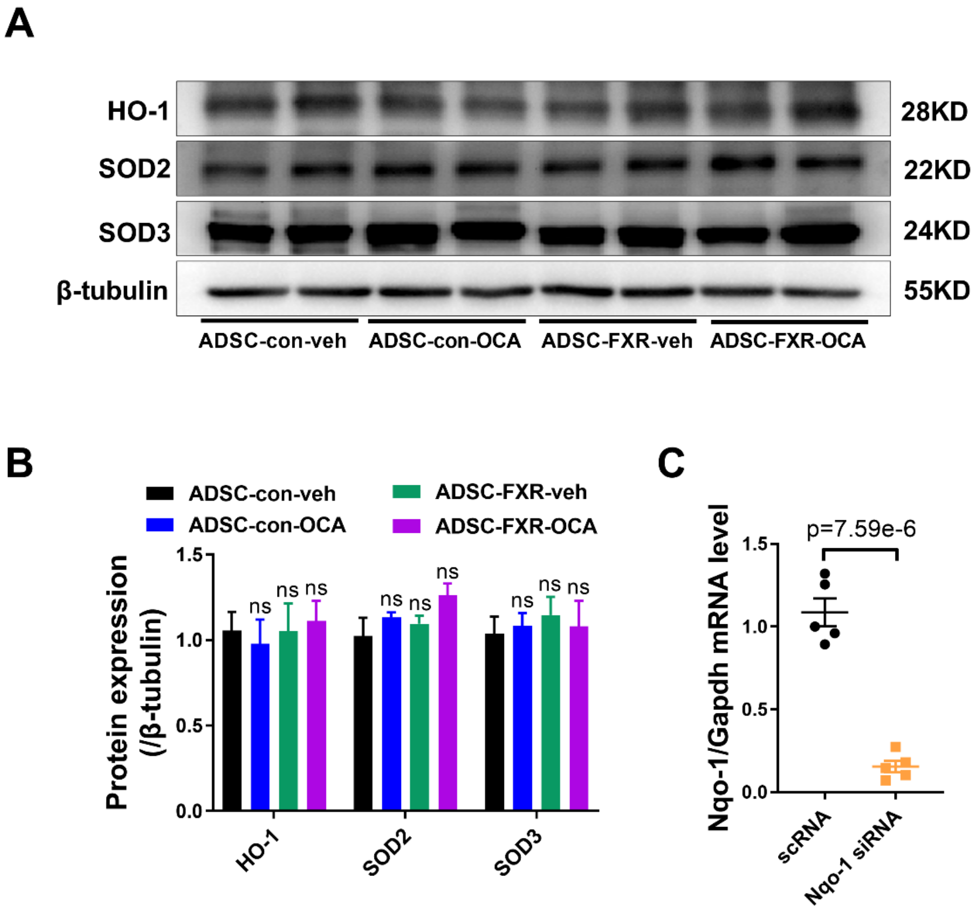

Online Figure 8. FXR overexpression with OCA administration did not increase the protein expression of HO-1, SOD2 or SOD3.

(A and B) Representative western blotting images and quantification of HO-1, SOD2 and SOD3 protein expression in ADSC (n=4 per group).

(C) The Nqo-1 mRNA level was determined by RT-PCR (n=5 per group).

Abbreviations: OCA: obeticholic acid. ADSC-con-veh: ADSC transfected with adenovirus control for 24 hours, followed by incubation with vehicle for another 24 hours. ADSC-FXR-veh: ADSC transfected with adenovirus-FXR for 24 hours, followed by incubation with vehicle for another 24 hours. ADSC-con-OCA: ADSC transfected with adenovirus control for 24 hours, followed by incubation with 10 μM OCA for another 24 hours. ADSC-FXR-OCA: ADSC transfected with adenovirus-FXR for 24 hours, followed by incubation with 10 μM OCA for another 24 hours.

Data in C were analyzed using unpaired Student's t test. Other data were analyzed using one-way ANOVA, followed by Bonferroni post hoc test. Data are presented as the mean ± SEM. ns: not significant.

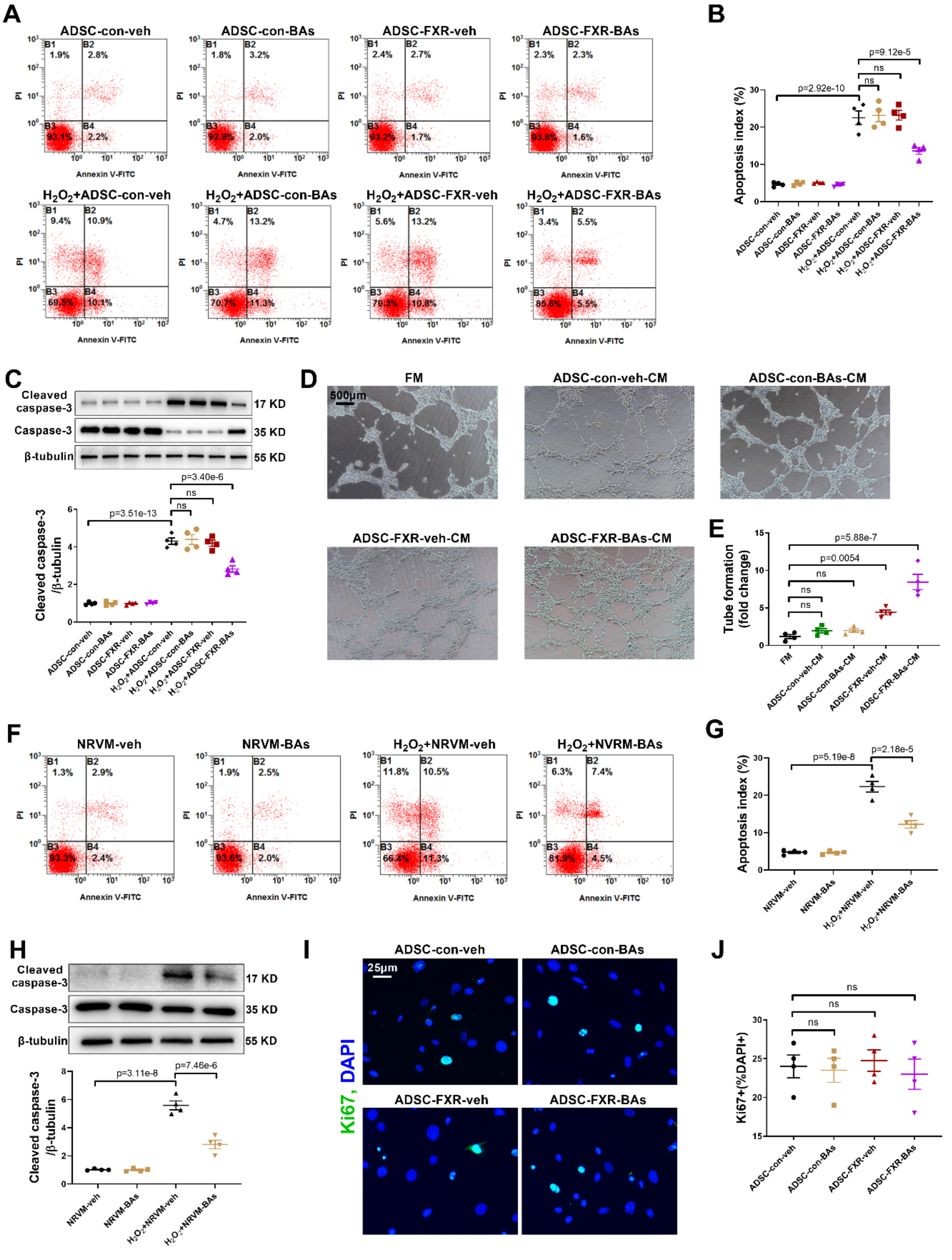

Online Figure 9. Bile acids at pathological concentrations promoted ADSC-FXR survival and paracrine angiogenesis and NRVM survival, but failed to affect ADSC-FXR proliferation.

(A) Flow cytometric analysis of ADSC-con-veh, ADSC-con-BAs, ADSC-FXR-veh and ADSC-FXR-BAs after treatment with H_2O_2 (200 µM) for 24 hours.

(B) ADSC apoptosis was quantified as the sum of the proportion of cells in quadrants B2 and B4 (n=4 per group).

(C) Representative western blotting images and quantification of the protein expression of cleaved caspase-3 and caspase-3 in ADSC. ADSC-con-veh, ADSC-con-BAs, ADSC-FXR-veh and ADSC-FXR-BAs were treated with H_2O_2 (200 μM) for 24 hours (n=4 per group).

(D) Tube formation assay of RCAEC was performed to evaluate the paracrine proangiogenic capability of ADSC-con-veh, ADSC-con-BAs, ADSC-FXR-veh and ADSC-FXR-BAs, and representative images are shown.

(E) Quantification of tube formation (n=4 per group).

(F) Flow cytometric analysis of NRVM-veh and NRVM-BAs after treatment with H_2O_2 (200 μM) for 8 hours.

(G) NRVM apoptosis was quantified as the sum of the proportion of cells in quadrants B2 and B4 (n=4 per group).

(H) Representative western blotting images and quantification of the protein expression of cleaved caspase-3 and caspase-3 in NRVM. NRVM-veh and NRVM-BAs were treated with H_2O_2 (200 μM) for 8 hours (n=4 per group).

(I) Representative images of Ki67+ stained ADSC.

(J) Quantification of Ki67+/DAPI+ ADSC (n=4 per group).

Abbreviations: BAs: a mixture of bile acids (1.5 μM CA, 40 nM DCA, 10 nM UCA, 30 nM UDCA, and 25 nM ACA). ADSC-con-veh: ADSC transfected with adenovirus control for 24 hours, followed by incubation with vehicle for another 24 hours. ADSC-FXR-veh: ADSC transfected with adenovirus-FXR for 24 hours, followed by incubation with vehicle for another 24 hours. ADSC-con-BAs: ADSC transfected with adenovirus control for 24 hours, followed by incubation with bile acids for another 24 hours. ADSC-FXR-BAs: ADSC transfected with adenovirus-FXR for 24 hours, followed by incubation with bile acids for another 24 hours. FM: fresh medium. CM: conditioned medium. NRVM: neonatal rat ventricular myocytes. NRVM-veh: NRVM were incubated with vehicle for 24 hours. NRVM-BAs: NRVM were incubated with bile acids for 24 hours.

All data were analyzed using one-way ANOVA, followed by Bonferroni post hoc test. Data are presented as the mean ± SEM. ns: not significant.

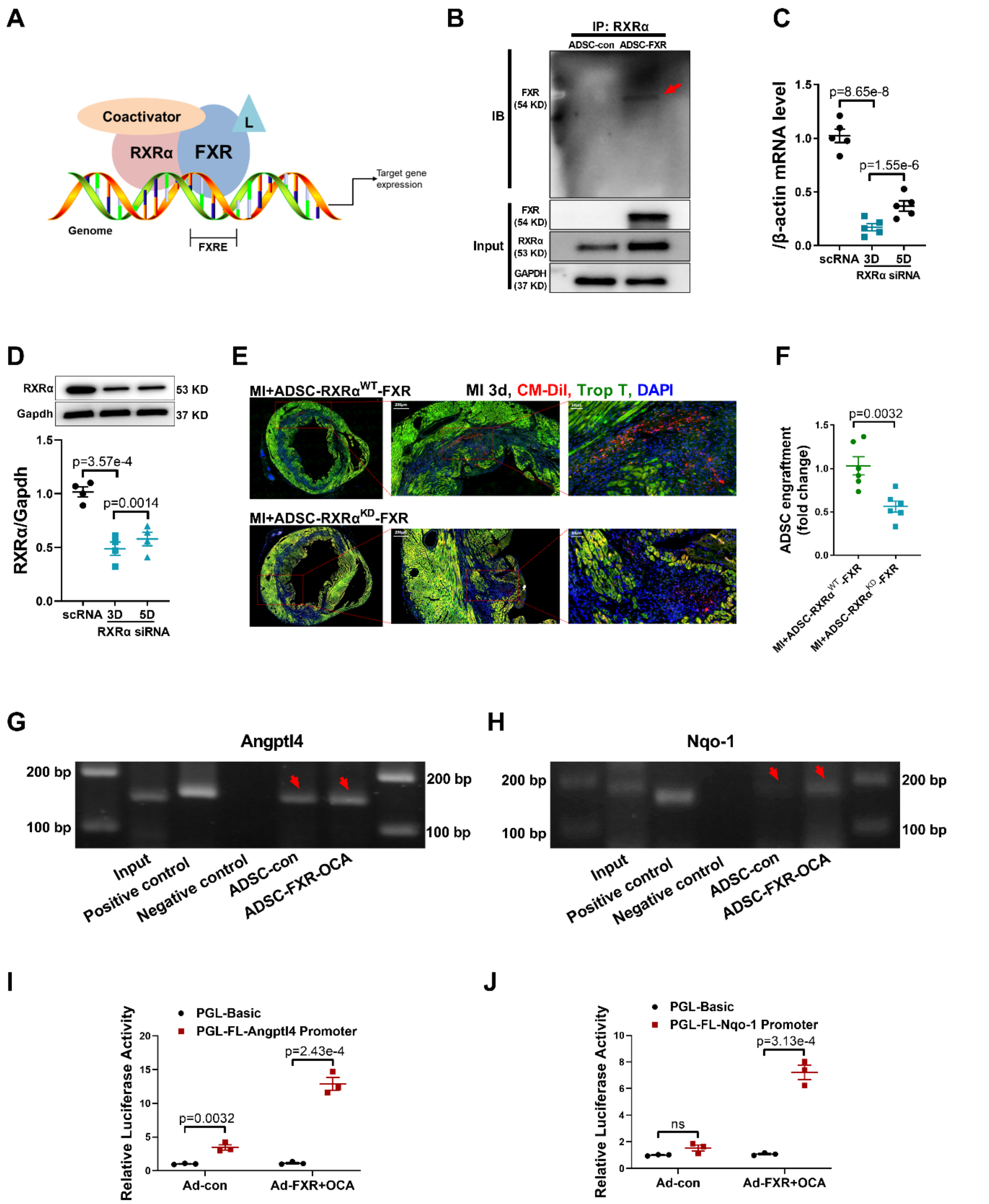

Online Figure 10. FXR overexpression-mediated promotion of the retention rate of ADSC were dependent on the heterodimer formed with RXRα, and the transcription of Angptl4 and Nqo-1 was directly regulated by FXR.

(A) The classical model by which FXR regulates downstream gene transcription. L means ligands.

(B) Co-immunoprecipitation (Co-IP) and immunoblot (IB) analysis of FXR with RXRα in ADSC.

(C) The RXRα mRNA level was determined by RT-PCR (n=5 per group).

(D) Representative western blotting images and quantification of RXRα protein expression in ADSC (n=4 per group).

(E) Representative images of CM-DiI-labeled ADSC in the ischemic heart 3 days after intramyocardial injection are shown.

(F) Quantification of CM-DiI-labeled ADSC 3 days after intramyocardial injection (n=6 per group).

(G) ChIP and PCR analysis of FXR binding to the Angptl4 promoter in ADSC. Anti-Histone H3 was used for positive control, and anti-IgG was used for negative control.

(H) ChIP and PCR analysis of FXR binding to the Nqo-1 promoter in ADSC. Anti-Histone H3 was used for positive control, and anti-IgG was used for negative control.

(I) Responses of the Full-length Angptl4 promoter reporter to Ad-con or Ad-FXR+OCA (n=3 per group).

(J) Responses of the Full-length Nqo-1 promoter reporter to Ad-con or Ad-FXR+OCA (n=3 per group).

Abbreviations: OCA: obeticholic acid. ADSC-con: ADSC transfected with adenovirus control for 2 days. ADSC-FXR: ADSC transfected with adenovirus-FXR for 2 days. ADSC-RXR^{WT}-FXR: ADSC transfected with scramble RNA for 24 hours and then transfected with adenovirus-FXR for another 24 hours. ADSC-RXR^{KD}-FXR: ADSC transfected with RXR siRNA for 24 hours and then transfected with adenovirus-FXR for another 24 hours. ADSC-FXR-OCA: ADSC transfected with adenovirus-FXR for 24 hours, followed by incubation with 10 μM OCA for another 24 hours. Data in F were analyzed using unpaired Student's t test. Other data were analyzed using one-way ANOVA, followed by Bonferroni post hoc test. Data are presented as the mean ± SEM. ns: not significant.

Online Table 1. Real Time PCR Primers

Genes	Forward primer (5'-3')	Reverse primer (5'-3')
**Angptl4**	CACCCACTTACACAGGCCG	GAAGTCCACAGAGCCGTTCA
**Nqo-1**	TCTCTGGCCGATTCAGAGTG	CCAGACGGTTTCCAGACGTT
**HO-1**	GAATCGAGCAGAACCAGCCT	AAGGAAGCCATCACCAGCTTA
**SOD2**	AGGAGAGTTGCTGGAGGCTA	AGCGGAATAAGGCCTGTTGTT
**SOD3**	TGCTGCTCGCTCACATAACA	GAGGTTCTCTGCACCTGTCA
**RXRα**	CATTTCCTGCCGCTCGACTT	GGGTGCTGATAGGAGAGTGC
**Nkx2-5**	TGACCCAGCCAAAGACCCT	CCATCCGTCTCGGCTTTGT
**Nppa**	GCTTCCAGGCCATATTGGAG	GGGGGCATGACCTCATCTT
**Gata4**	CCCTACCCAGCCTACATGG	ACATATCGAGATTGGGGTGTCT
**VE-cad**	CACTGCTTTGGGAGCCTTC	GGGGCAGCGATTCATTTTTCT
**CD31**	CTGCCAGTCCGAAAATGGAAC	CTTCATCCACCGGGGCTATC
**eNOS**	GGCTGGGTTTAGGGCTGTG	CTGAGGGTGTCGTAGGTGATG
**a-SMA**	GTCCCAGACATCAGGGAGTAA	TCGGATACTTCAGCGTCAGGA
**CNN**	TCTGCACATTTTAACCGAGGTC	GCCAGCTTGTTCTTTACTTCAGC
**Myh11**	AAGCTGCGGCTAGAGGTCA	CCCTCCCTTTGATGGCTGAG
**Pparg**	TCGCTGATGCACTGCCTATG	GAGAGGTCCACAGAGCTGATT
**Adipoq**	TGTTCCTCTTAATCCTGCCCA	CCAACCTGCACAAGTTCCCTT
**Alp**	TGGGCATTGTGACTACCACTCGG	CCTCTGGTGGCATCTCGTTATCC
**Runx2**	GGGAACCAAGAAGGCACAGA	ACTTGGTGCAGAGTTCAGGG
**CD73**	TCCTGCAAGTGGGTGGAATC	AGATGGGCACTCGACACTTG
**CD90**	TCTCCTGCTCTCAGTCTTGC	TATTCTCATGGCGGCAGTCC
**CD105**	GATACCGGATAAGGCCCAGC	TTCTGCGAGACCTGTTGTGG
**β-actin**	CATCCGTAAAGACCTCTATGCCAAC	ATGGAGCCACCGATCCACA
**tdTomato**	CTCCGAGGACAACAACATGG	CTTGTACAGCTCGTCCATGC
**Gapdh**	GGTGAAGGTCGGTGTGAACG	CTCGCTCCTGGAAGATGGTG

Online Table 2. Full-length Angptl4 and Nqo-1 promoter sequences

Genes	Promoter sequence
Angptl4	GTGGCCTGCATGTCAGCCTTGAGGCATGCAGTTTCAGATTCCAGAGGTTTGC<br>TGCTGGCTCCCAGCCCTGCTGACTCAACTTGGGGGTTTTTCAGTCAATTTGC<br>CCTAAGGGTCTTCTTGTCAGACAGGTTTTCTGTGCCAGGCTTCAGGGGAACT<br>GTCAGAAAAAGGAACACCAATGCAAACTTGTTGAATAAAAACTTAACCAGG<br>CAGCATAAACAAGGAAAGCTGTCTGCCCCACCCCAGTCCTCTTTTGTCTCTG<br>CCTTGTCCCTCTCCCCTGCTCAAAGGTAGGATTTATACACAAACTAAAAAAC<br>TCAGTACAGGATCTAGGCTGTATGGAATGAGTGAATGAATGGATGGGTGTG<br>GAAGGGAAATCTTTGCTGATGTTCTCTAGATTGTTTGCTTTTTAAATTTTTTT<br>TAAAAAAGATGTATTTATTTTATTTATATGAGTACATTGTAGCTGTCTTCAG<br>ACACACCGGAAGAGGGTATCAGATCCTATTACAGATGGTTGTAAGCCACCA<br>TGTGGTTGCTGGGAATTAACTCAGGACCTTTGGAAGAGCAGTCAGTACTCTT<br>AACTGCTGAACCATCTCTCCAGCCTGCTTTTTAAATGTTTAAATGACTGTTTT<br>AAATTGTGTGCATGTGTGTGAGTGCCTGAGGAGGTCAAAGTCACCATAGCT<br>CCTGGAGCTGGAATTATAGGTGACTGTGAGCTGTTGGACTTAGATCCCCTGC<br>AAAAGATGTAAATGCTCTTAACTGGTAAGATAGATTTCCAGTTCCTCCTGGC<br>TTTTTTAGTTGTTGTTGTTGTTGTTTTGTGACAAGGTCTTCTTCCTGGATGGC<br>CTGGAACTTCACTATGTAGACCAGGCTGACCTCAAACTCACAGAGCTTCCTC<br>CAATTGCTTCTTGCTCTGCAGTGCTGGGAGTTAAAGTATGCATAACTATGAA<br>TTGTTTTTGTTTTGTTTTGAAACAAGGTCTTGCTGTTTCAGCGTTGACTGGTC<br>TGTAACTTACTATGTAGAGCAGGCTAGCTTGTAGCTTGTACTATTCCCCTGT<br>GTCCTGCGTGATGGGTTTACAGGTGCGTGCCACCAGTTTTGTTGTTGCTGTT<br>GTTGTTTGGAGACCTGGCTCAGAGTAGCCCAGGCTCTCTTAAACTCACTTGT<br>GTAGCTCAAGATGAATTCCACACTCTTCTGCTCCATGCCCCAAGAGCTGGGA<br>TGACAGGTCTGTCCTCAGACACTGATCCTCCTTTAACGCAGGTAAATTCCTG<br>GACACCAAGACCCCTGGTGGTGGAGGACAGGCAACAGGAGGAAGCCTAGG<br>GTTGCAATCTCTACAATGTAGACAGAAATCCCAGCTATTGACTATTTGGCCA<br>GGAAGAGGTGACTAATTGAACAAGGGTGTTAGTGATCCTCACAAACACATC<br>TGCAATGACGAATGCTTGAGCTGGAAAGAGCGTGGGAGCCAGAAAGGTGGT<br>GGAAGTGAGAGTGGGGGCAGGGGTTCCGCCTGGTCTGTGGATTAAAGATCT<br>CAGGAAGCCTCTAAGAGGCAGAGAGCAAGGTTCTGTAATCCCCCACTTGCC<br>ATCTGAACTCAGCCTACCAGGGAGAGAACTTTCTGCCACGTAACTCTGCCCT<br>ACTTTGCTTTTGACTTGAGAAGGGGTCAGTCAAGAAGGCATCCACGTAGCG<br>GGAAGGGGAAAAAAAAAAAAGAAGGCATCTACGTCCTGATTTGTACGCCCT<br>TTCCTCCATTGAACCGGGCTGGGATGCGGGCTAGGGCTGGGAATTTCCGGCC<br>TTAGGATGAACCATGCAGTGCACAGACAGTCACGTCGCTTATTAGGTCGCA<br>AGGAAGGCGAAGCAGCCATCCCCAAGAACTCTTCATACACACACACACACA<br>CACACACACACACACACACACACACGCCACACACACACACTCGTAACTACA

	AAGCCTGTGGCATTGCACCCTAAAACTTGGCCACTCGCTCCGCCCCCGGGCC<br>CCGCCTCCAATGCTCTCCCTCCCACTCCCACACCC
**Nqo-1**	CAATCCAAGAGCTGAGAGTGTCATCAGATAGCATCTTGGACCATAAACCCC<br>ACCCCCCCATCTCGATGTACCCACCTCTCCAGTGTACCCACCTCTCCAGGGT<br>TCCCACCAACGTACTTTAAAGTCGCCTTAACTTATTACTTTGTTCTGTAAATT<br>GTAAAAATCTTGTGAGGCTGCACCCACATTGGAACATAAAATTTGGGGTAC<br>CCTATATTGGTGTTCCCAGGCCGTGGCCACTCAAAATGGCTCCGGAATAAAC<br>TGTGGTTTTTATGTTGGTATTTCTTTACAGCTGAGAGATGACCCTCAGGCAC<br>AAACTGTAAGGAAACACCAGAAAACCCAATGTTGAACATTGTAGTGTGGTG<br>TGTTTGGTATTTGTCTGATGCTGAGACAGAAGCAGCCTGTGGAAACTACTTT<br>AGCACGTTGTTTGAGAGGAGAGTCTGACATCGCAGACGAAGCTGCCTGGTC<br>ACACAGCATCCAGTGAGAAAGCAGATAGCCTTTCCTGATGAAGCTATATCC<br>CCAGCTCCTATTTTTGATTTTTGAAATGAGGTCTCACGTATCTTCAACTGTAG<br>TAGTTAAGCAAGGCCAAGCCTTCAAGGGTCAAGGGTCCCTGTGTTAAGAAA<br>CCTTCAGGGTATCCTTTTATTTCCAGATAAAGCTTTGTGGTGCAAACTGGGC<br>ATGTGCAGGAATGTACCCTCCCCAAAGTACCTCCCCCATAGGAACAAGAAT<br>CTGCCTCCCTAATAAGTTCTCACCTTTTCTTTGTTTGGGAAGTGCGTTGCTTT<br>AAAAATTGTCCTTGGTCTCTTTGCCTGCTGCAGCTGATATTTCTTTCTTCCCT<br>CTTATAGCCCATTGTGTTACTTCGGCTTTGCCCTAACCGGAATGAGACTCCA<br>CTGAGATCCATCACAAGGGCTGTCACTAGCCTGTCACCACAGGAGACTGCA<br>CGTGTGTCTTTCCCTCACTGTCAGAATTTATCAGATAACAGGAAAGTTGAGC<br>TCTTTTAATGATGGCAATCTGTTATAATGTCTACCCTACTTTGGGAGCTTGAC<br>CAAGGCACACACATACATGTAGGGTCTTTATTCCAACCCTTTTTCTTTCTTTC<br>TCTGTTTTTGAGACAGGGTCTCACCTATGTAGCCCTGGCTGTTGTTCTAGCAC<br>TAGCTATGTGGACCAGTCTGGCCTTAAACTCAGAGATCTGCCTGCTTCTGGC<br>ACCCAAGTGATGGGATTAAAAGCATGTACTAGTCGTGGTGGCGCACTCCTTT<br>AATCCCAGCACTTGGGAGGCAGAGGCAGGTGGATTTCTGAGTTCGAGGCCA<br>GCCTGGTCTACAGAGTGAGTTCCAGGACAGCCAGGGCTACACAGAGAAACA<br>CTTCCCTGTGGCTGCTGTCTCCTGCCAGCTTTGCTCTGGCTTCCTAGAGAGTC<br>GCACAGATGAGTTCCCCGTTACCTGGTCACCGGGGGCAAAGTTGAGGAGAC<br>CCAAGTGTGTATACCCAGGGAGCAGTTTTTGCAGTTTCTAAGAGCAGAACG<br>CAGCACGAATTCATTTCACACGAGGACAAGTCTCTCTGAACTTTCAGTCTAG<br>AGTCACAGTGAGTCGGCAAAATTTGAGCCCATCCGTTTTGCTGCCCCACCCT<br>TCCCCTAGCGTGCAAAGGTGACTTCCCACGGCTGAGTGAGGACTAATCTAC<br>ACAGGCTGATTATGTAGGCAGGTCCCACGAAGCTCGAAAAATTCTGTTGGA<br>AATTTCCATTTTGTACCCAGGAGGTCTTGGGACAGGGAGCAGATGAATTTAT<br>TCAATATGTCCCCATATCCTCAAATTCTCTCTCACTCCCTTGCTCCCGGGGAA<br>CCCTTTTGACTTCCATATACAGGAGTCCTAGTCCAGCCCCAAACTGCTTCTC<br>CCTGCCAAAAACTTGGTATCTTCCCAAGATGCCTCTGGGTTCTGGAGTCCAG

	CCCCGCCCTCGCTGGCTGCTCTGCACAGTGGGCTGGGCGGGCATAAGCAGG ATATAAAGCCTTCGCTCAGCCCATACCCCAA
