# Supplementary material for: Mesenchymal Stromal Cells Overexpressing Farnesoid X Receptor Exert Cardioprotective Effects Against Acute Ischemic Heart Injury by Binding Endogenous Bile Acids
Source: Adv Sci (Weinh). 2022 Jul 3;9(24):2200431. doi: 10.1002/advs.202200431 (PMC9404394; doi:10.1002/advs.202200431)
Supplement: Supplementary file 2 — Supporting Table 1 [file ADVS-9-2200431-s001.pdf]

SUPPLEMENTARY TABLES OF LC-MS/MS

Protein accession	Protein description	Gene name	Ratio	P.value	Regulated.Type
P55097	Cathepsin K OS=Mus musculus OX=10090 GN=Ctsk PE=1 SV=2	Ctsk	23.158	9.59156E-05	Up
Q6PIP5	NudC domain-containing protein 1 OS=Mus musculus OX=10090 GN=Nudcd1 PE=1 SV=2	Nudcd1	4.444	9.25721E-05	Up
Q58A65	C-Jun-amino-terminal kinase-interacting protein 4 OS=Mus musculus OX=10090 GN=Spag9 PE=1 SV=2	Spag9	9.216	9.08153E-05	Up
Q05D44	Eukaryotic translation initiation factor 5B OS=Mus musculus OX=10090 GN=Eif5b PE=1 SV=2	Eif5b	3.094	8.62175E-05	Up
Q9D281	Protein Noxp20 OS=Mus musculus OX=10090 GN=Fam114a1 PE=1 SV=1	Fam114a1	3.534	8.38088E-05	Up
P13597	Intercellular adhesion molecule 1 OS=Mus musculus OX=10090 GN=Icam1 PE=1 SV=1	Icam1	4.889	8.07037E-05	Up
Q9Z2M7	Phosphomannomutase 2 OS=Mus musculus OX=10090 GN=Pmm2 PE=1 SV=1	Pmm2	5.486	7.73271E-05	Up
Q3UE37	Ubiquitin-conjugating enzyme E2 Z OS=Mus musculus OX=10090 GN=Ube2z PE=1 SV=2	Ube2z	5.022	7.5263E-05	Up
P97311	DNA replication licensing factor MCM6 OS=Mus musculus OX=10090 GN=Mcm6 PE=1 SV=1	Mcm6	3.749	7.36627E-05	Up
P52624	Uridine phosphorylase 1 OS=Mus musculus OX=10090 GN=Upp1 PE=1 SV=2	Upp1	5.03	6.71458E-05	Up
Q9Z1P8	Angiopoietin-related protein 4 OS=Mus musculus OX=10090 GN=Angptl4 PE=1 SV=1	Angptl4	3.326	6.58374E-05	Up
Q7TSS2	Ubiquitin-conjugating enzyme E2 Q1 OS=Mus musculus OX=10090 GN=Ube2q1 PE=1 SV=2	Ube2q1	3.015	6.476E-05	Up
Q91YS8	Calcium/calmodulin-dependent protein kinase type 1 OS=Mus musculus OX=10090 GN=Camk1 PE=1 SV=1	Camk1	11.29	6.42331E-05	Up
Q9D358	Low molecular weight phosphotyrosine protein phosphatase OS=Mus musculus OX=10090 GN=Acp1 PE=1 SV=3	Acp1	6.192	6.40464E-05	Up
Q9R1Q8	Transgelin-3 OS=Mus musculus OX=10090 GN=Tagln3 PE=1 SV=1	Tagln3	4.517	6.36965E-05	Up
O08808	Protein diaphanous homolog 1 OS=Mus musculus OX=10090 GN=Diaph1 PE=1 SV=1	Diaph1	3.766	6.23522E-05	Up
Q8CJ53	Cdc42-interacting protein 4 OS=Mus musculus OX=10090 GN=Trip10 PE=1 SV=2	Trip10	6.461	6.14083E-05	Up
P19096	Fatty acid synthase OS=Mus musculus OX=10090 GN=Fasn PE=1 SV=2	Fasn	4.757	6.10107E-05	Up
P63028	Translationally-controlled tumor protein OS=Mus musculus OX=10090 GN=Tpt1 PE=1 SV=1	Tpt1	3.209	6.03381E-05	Up
P10630	Eukaryotic initiation factor 4A-II OS=Mus musculus OX=10090 GN=Eif4a2 PE=1 SV=2	Eif4a2	4.005	5.99038E-05	Up
Q8K310	Matrin-3 OS=Mus musculus OX=10090 GN=Matr3 PE=1 SV=1	Matr3	3.728	5.8691E-05	Up
Q9Z2V5	Histone deacetylase 6 OS=Mus musculus OX=10090 GN=Hdac6 PE=1 SV=3	Hdac6	3.168	5.82039E-05	Up
Q9EQQ9	Protein O-GlcNAcase OS=Mus musculus OX=10090 GN=Oga PE=1 SV=2	Oga	6.658	5.75781E-05	Up
P47811	Mitogen-activated protein kinase 14 OS=Mus musculus OX=10090 GN=Mapk14 PE=1 SV=3	Mapk14	16.517	5.74251E-05	Up
Q9CR86	Calcium-regulated heat stable protein 1 OS=Mus musculus OX=10090 GN=Carhsp1 PE=1 SV=1	Carhsp1	5.363	5.58117E-05	Up
Q8CJG0	Protein argonaute-2 OS=Mus musculus OX=10090 GN=Ago2 PE=1 SV=3	Ago2	5.344	5.5674E-05	Up
Q07813	Apoptosis regulator BAX OS=Mus musculus OX=10090 GN=Bax PE=1 SV=1	Bax	3.928	5.21324E-05	Up
Q9DAS9	Guanine nucleotide-binding protein G(I)/G(S)/G(O) subunit gamma-12 OS=Mus musculus OX=10090 GN=Gng12 PE=1 SV=3	Gng12	3.341	4.86436E-05	Up

P60521	Gamma-aminobutyric acid receptor-associated protein-like 2 OS=Mus musculus OX=10090 GN=Gabarapl2 PE=1 SV=1	Gabarapl2	7.603	4.81891E-05	Up
P97351	40S ribosomal protein S3a OS=Mus musculus OX=10090 GN=Rps3a PE=1 SV=3	Rps3a	3.237	4.79205E-05	Up
Q9CXW4	60S ribosomal protein L11 OS=Mus musculus OX=10090 GN=Rpl11 PE=1 SV=4	Rpl11	3.183	4.68924E-05	Up
Q6PB44	Tyrosine-protein phosphatase non-receptor type 23 OS=Mus musculus OX=10090 GN=Ptpn23 PE=1 SV=2	Ptpn23	3.666	4.55299E-05	Up
Q8K2K6	Arf-GAP domain and FG repeat-containing protein 1 OS=Mus musculus OX=10090 GN=Agfg1 PE=1 SV=2	Agfg1	3.89	4.52155E-05	Up
P12382	ATP-dependent 6-phosphofructokinase, liver type OS=Mus musculus OX=10090 GN=Pfkl PE=1 SV=4	Pfkl	3.115	4.4851E-05	Up
Q6ZQ88	Lysine-specific histone demethylase 1A OS=Mus musculus OX=10090 GN=Kdm1a PE=1 SV=2	Kdm1a	5.23	4.10609E-05	Up
Q811D0	Disks large homolog 1 OS=Mus musculus OX=10090 GN=Dlg1 PE=1 SV=1	Dlg1	5.697	3.90405E-05	Up
Q921I2	Kelch domain-containing protein 4 OS=Mus musculus OX=10090 GN=Klhdc4 PE=2 SV=2	Klhdc4	8.299	3.76453E-05	Up
Q8BUV3	Gephyrin OS=Mus musculus OX=10090 GN=Gphn PE=1 SV=2	Gphn	3.189	3.64128E-05	Up
Q8CD91	SPARC-related modular calcium-binding protein 2 OS=Mus musculus OX=10090 GN=Smoc2 PE=1 SV=1	Smoc2	14.436	3.61129E-05	Up
Q9JLJ2	4-trimethylaminobutyraldehyde dehydrogenase OS=Mus musculus OX=10090 GN=Aldh9a1 PE=1 SV=1	Aldh9a1	5.624	3.60971E-05	Up
Q6PDQ2	Chromodomain-helicase-DNA-binding protein 4 OS=Mus musculus OX=10090 GN=Chd4 PE=1 SV=1	Chd4	9.678	3.60142E-05	Up
P31230	Aminoacyl tRNA synthase complex-interacting multifunctional protein 1 OS=Mus musculus OX=10090 GN=Aimp1 PE=1 SV=2	Aimp1	4.75	3.53661E-05	Up
Q9D0I8	mRNA turnover protein 4 homolog OS=Mus musculus OX=10090 GN=Mrto4 PE=1 SV=1	Mrto4	22.932	3.48124E-05	Up
Q99KR3	Endoribonuclease LACTB2 OS=Mus musculus OX=10090 GN=Lactb2 PE=1 SV=1	Lactb2	7.329	3.43105E-05	Up
P60670	Nuclear protein localization protein 4 homolog OS=Mus musculus OX=10090 GN=Nploc4 PE=1 SV=3	Nploc4	3.631	3.40865E-05	Up
O08528	Hexokinase-2 OS=Mus musculus OX=10090 GN=Hk2 PE=1 SV=1	Hk2	9.369	3.29025E-05	Up
Q9D5V6	Synapse-associated protein 1 OS=Mus musculus OX=10090 GN=Syap1 PE=1 SV=1	Syap1	13.721	3.19072E-05	Up
Q99KB8	Hydroxyacylglutathione hydrolase, mitochondrial OS=Mus musculus OX=10090 GN=Hagh PE=1 SV=2	Hagh	126.046	3.03101E-05	Up
Q921H8	3-ketoacyl-CoA thiolase A, peroxisomal OS=Mus musculus OX=10090 GN=Acaa1a PE=1 SV=1	Acaa1a	8.103	2.93407E-05	Up
P35278	Ras-related protein Rab-5C OS=Mus musculus OX=10090 GN=Rab5c PE=1 SV=2	Rab5c	3.001	2.91798E-05	Up
Q9QZ08	N-acetyl-D-glucosamine kinase OS=Mus musculus OX=10090 GN=Nagk PE=1 SV=3	Nagk	4.406	2.75335E-05	Up
P34022	Ran-specific GTPase-activating protein OS=Mus musculus OX=10090 GN=Ranbp1 PE=1 SV=2	Ranbp1	3.019	2.7202E-05	Up

P55302	Alpha-2-macroglobulin receptor-associated protein OS=Mus musculus OX=10090 GN=Lrpap1 PE=1 SV=1	Lrpap1	5.209	2.60167E-05	Up
Q3UMT1	Protein phosphatase 1 regulatory subunit 12C OS=Mus musculus OX=10090 GN=Ppp1r12c PE=1 SV=1	Ppp1r12c	4.951	2.54839E-05	Up
Q9Z0E6	Guanylate-binding protein 2 OS=Mus musculus OX=10090 GN=Gbp2 PE=1 SV=1	Gbp2	3.62	2.43296E-05	Up
O88587	Catechol O-methyltransferase OS=Mus musculus OX=10090 GN=Comt PE=1 SV=2	Comt	3.025	2.43292E-05	Up
P50429	Arylsulfatase B OS=Mus musculus OX=10090 GN=Arsb PE=1 SV=3	Arsb	4.812	2.39064E-05	Up
Q08091	Calponin-1 OS=Mus musculus OX=10090 GN=Cnn1 PE=1 SV=1	Cnn1	3.771	2.35274E-05	Up
Q9JHU9	Inositol-3-phosphate synthase 1 OS=Mus musculus OX=10090 GN=Isyna1 PE=1 SV=1	Isyna1	6.597	2.29186E-05	Up
P98078	Disabled homolog 2 OS=Mus musculus OX=10090 GN=Dab2 PE=1 SV=2	Dab2	4.822	2.23354E-05	Up
Q60676	Serine/threonine-protein phosphatase 5 OS=Mus musculus OX=10090 GN=Ppp5c PE=1 SV=3	Ppp5c	4.163	2.15205E-05	Up
P61750	ADP-ribosylation factor 4 OS=Mus musculus OX=10090 GN=Arf4 PE=1 SV=2	Arf4	4.465	2.13859E-05	Up
P60867	40S ribosomal protein S20 OS=Mus musculus OX=10090 GN=Rps20 PE=1 SV=1	Rps20	4.212	1.98814E-05	Up
P62911	60S ribosomal protein L32 OS=Mus musculus OX=10090 GN=Rpl32 PE=1 SV=2	Rpl32	5.846	1.96393E-05	Up
Q8BK67	Protein RCC2 OS=Mus musculus OX=10090 GN=Rcc2 PE=1 SV=1	Rcc2	5.09	1.87201E-05	Up
P61164	Alpha-centractin OS=Mus musculus OX=10090 GN=Actr1a PE=1 SV=1	Actr1a	8.042	1.75575E-05	Up
Q3U7R1	Extended synaptotagmin-1 OS=Mus musculus OX=10090 GN=Esyt1 PE=1 SV=2	Esyt1	8.84	1.74233E-05	Up
Q9D0M1	Phosphoribosyl pyrophosphate synthase-associated protein 1 OS=Mus musculus OX=10090 GN=Prpsap1 PE=1 SV=1	Prpsap1	9.598	1.73756E-05	Up
Q9D7G0	Ribose-phosphate pyrophosphokinase 1 OS=Mus musculus OX=10090 GN=Prps1 PE=1 SV=4	Prps1	10.613	1.69694E-05	Up
P62334	26S proteasome regulatory subunit 10B OS=Mus musculus OX=10090 GN=Psmc6 PE=1 SV=1	Psmc6	4.514	1.68423E-05	Up
Q9D832	DnaJ homolog subfamily B member 4 OS=Mus musculus OX=10090 GN=Dnajb4 PE=1 SV=1	Dnajb4	3.047	1.6583E-05	Up
Q9WVJ3	Carboxypeptidase Q OS=Mus musculus OX=10090 GN=Cpq PE=1 SV=1	Cpq	4.104	1.56117E-05	Up
P51125	Calpastatin OS=Mus musculus OX=10090 GN=Cast PE=1 SV=2	Cast	6.04	1.5188E-05	Up
Q9CX56	26S proteasome non-ATPase regulatory subunit 8 OS=Mus musculus OX=10090 GN=Psmd8 PE=1 SV=2	Psmd8	5.5	1.4845E-05	Up
Q99MR6	Serrate RNA effector molecule homolog OS=Mus musculus OX=10090 GN=Srrt PE=1 SV=1	Srrt	7.934	1.47573E-05	Up
P10711	Transcription elongation factor A protein 1 OS=Mus musculus OX=10090 GN=Tcea1 PE=1 SV=2	Tcea1	3.04	1.46797E-05	Up
O70310	Glycylpeptide N-tetradecanoyltransferase 1 OS=Mus musculus OX=10090 GN=Nmt1 PE=1 SV=1	Nmt1	3.122	1.4272E-05	Up
P62751	60S ribosomal protein L23a OS=Mus musculus OX=10090 GN=Rpl23a PE=1 SV=1	Rpl23a	3.644	1.41151E-05	Up
Q921J2	GTP-binding protein Rheb OS=Mus musculus OX=10090 GN=Rheb PE=1 SV=1	Rheb	5.092	1.2941E-05	Up
P84244	Histone H3.3 OS=Mus musculus OX=10090 GN=H3-3a PE=1 SV=2	H3-3a	3.872	1.2702E-05	Up
Q3UGS4	Mapk-regulated corepressor-interacting protein 1 OS=Mus musculus OX=10090 GN=Mcrip1 PE=1 SV=1	Mcrip1	6.394	1.25306E-05	Up

Q60716	Prolyl 4-hydroxylase subunit alpha-2 OS=Mus musculus OX=10090 GN=P4ha2 PE=1 SV=1	P4ha2	3.072	1.22261E-05	Up
P21981	Protein-glutamine gamma-glutamyltransferase 2 OS=Mus musculus OX=10090 GN=Tgm2 PE=1 SV=4	Tgm2	7.167	1.18019E-05	Up
Q8VE88	Protein FAM114A2 OS=Mus musculus OX=10090 GN=Fam114a2 PE=1 SV=2	Fam114a2	10.43	1.15786E-05	Up
Q9D6R2	Isocitrate dehydrogenase [NAD] subunit alpha, mitochondrial OS=Mus musculus OX=10090 GN=Idh3a PE=1 SV=1	Idh3a	6.628	1.10175E-05	Up
Q91VH6	Protein MEMO1 OS=Mus musculus OX=10090 GN=Memo1 PE=1 SV=1	Memo1	4.505	1.0582E-05	Up
P99029	Peroxiredoxin-5, mitochondrial OS=Mus musculus OX=10090 GN=Prdx5 PE=1 SV=2	Prdx5	3.738	1.05279E-05	Up
Q921M7	CYFIP-related Rac1 interactor B OS=Mus musculus OX=10090 GN=Cyrib PE=1 SV=1	Cyrib	3.696	1.00677E-05	Up
O08539	Myc box-dependent-interacting protein 1 OS=Mus musculus OX=10090 GN=Bin1 PE=1 SV=1	Bin1	9.317	1.00547E-05	Up
Q6P069	Sorcin OS=Mus musculus OX=10090 GN=Sri PE=1 SV=1	Sri	9.658	9.29966E-06	Up
P62254	Ubiquitin-conjugating enzyme E2 G1 OS=Mus musculus OX=10090 GN=Ube2g1 PE=1 SV=3	Ube2g1	3.218	9.17292E-06	Up
P14602	Heat shock protein beta-1 OS=Mus musculus OX=10090 GN=Hspb1 PE=1 SV=3	Hspb1	3.636	8.94461E-06	Up
Q9EQU5	Protein SET OS=Mus musculus OX=10090 GN=Set PE=1 SV=1	Set	4.766	8.93505E-06	Up
P14148	60S ribosomal protein L7 OS=Mus musculus OX=10090 GN=Rpl7 PE=1 SV=2	Rpl7	4.771	8.66208E-06	Up
Q8K1J6	CCA tRNA nucleotidyltransferase 1, mitochondrial OS=Mus musculus OX=10090 GN=Trnt1 PE=1 SV=1	Trnt1	7.852	8.60281E-06	Up
Q9R1P4	Proteasome subunit alpha type-1 OS=Mus musculus OX=10090 GN=Psma1 PE=1 SV=1	Psma1	3.74	8.35477E-06	Up
Q80SW1	S-adenosylhomocysteine hydrolase-like protein 1 OS=Mus musculus OX=10090 GN=Ahcyl1 PE=1 SV=1	Ahcyl1	5.139	8.18787E-06	Up
P97384	Annexin A11 OS=Mus musculus OX=10090 GN=Anxa11 PE=1 SV=2	Anxa11	3.511	6.81441E-06	Up
P28667	MARCKS-related protein OS=Mus musculus OX=10090 GN=Marcksl1 PE=1 SV=2	Marcksl1	4.193	6.58384E-06	Up
P62918	60S ribosomal protein L8 OS=Mus musculus OX=10090 GN=Rpl8 PE=1 SV=2	Rpl8	3.352	5.9286E-06	Up
Q04447	Creatine kinase B-type OS=Mus musculus OX=10090 GN=Ckb PE=1 SV=1	Ckb	5.412	5.81545E-06	Up
P47753	F-actin-capping protein subunit alpha-1 OS=Mus musculus OX=10090 GN=Capza1 PE=1 SV=4	Capza1	4.122	5.38691E-06	Up
P62307	Small nuclear ribonucleoprotein F OS=Mus musculus OX=10090 GN=Snrpf PE=1 SV=1	Snrpf	3.672	5.33918E-06	Up
Q9QZQ1	Afadin OS=Mus musculus OX=10090 GN=Afdn PE=1 SV=3	Afdn	8.371	5.31709E-06	Up
Q9DB05	Alpha-soluble NSF attachment protein OS=Mus musculus OX=10090 GN=Napa PE=1 SV=1	Napa	4.417	5.25258E-06	Up
Q9DB29	Isoamyl acetate-hydrolyzing esterase 1 homolog OS=Mus musculus OX=10090 GN=Iah1 PE=1 SV=1	Iah1	3.469	5.12276E-06	Up
P26883	Peptidyl-prolyl cis-trans isomerase FKBP1A OS=Mus musculus OX=10090 GN=Fkbp1a PE=1 SV=2	Fkbp1a	3.269	4.88652E-06	Up
Q8BP92	Reticulocalbin-2 OS=Mus musculus OX=10090 GN=Rcn2 PE=1 SV=1	Rcn2	3.439	4.71585E-06	Up
P35821	Tyrosine-protein phosphatase non-receptor type 1 OS=Mus musculus OX=10090 GN=Ptpn1 PE=1 SV=2	Ptpn1	3.9	4.52634E-06	Up

P47911	60S ribosomal protein L6 OS=Mus musculus OX=10090 GN=Rpl6 PE=1 SV=3	Rpl6	4.362	3.88479E-06	Up
Q99JF5	Diphosphomevalonate decarboxylase OS=Mus musculus OX=10090 GN=Mvd PE=1 SV=2	Mvd	4.795	3.7699E-06	Up
P83870	PHD finger-like domain-containing protein 5A OS=Mus musculus OX=10090 GN=Phf5a PE=1 SV=1	Phf5a	3.103	3.76168E-06	Up
Q91V12	Cytosolic acyl coenzyme A thioester hydrolase OS=Mus musculus OX=10090 GN=Acot7 PE=1 SV=2	Acot7	3.9	3.53131E-06	Up
P97461	40S ribosomal protein S5 OS=Mus musculus OX=10090 GN=Rps5 PE=1 SV=3	Rps5	4.842	3.48121E-06	Up
P08030	Adenine phosphoribosyltransferase OS=Mus musculus OX=10090 GN=Aprt PE=1 SV=2	Aprt	3.319	3.39372E-06	Up
Q9Z1Q9	Valine--tRNA ligase OS=Mus musculus OX=10090 GN=Vars1 PE=1 SV=1	Vars1	4.563	3.35647E-06	Up
Q9EQK5	Major vault protein OS=Mus musculus OX=10090 GN=Mvp PE=1 SV=4	Mvp	3.439	3.34582E-06	Up
Q9R0G6	Cartilage oligomeric matrix protein OS=Mus musculus OX=10090 GN=Comp PE=1 SV=2	Comp	253.867	3.29889E-06	Up
P63038	60 kDa heat shock protein, mitochondrial OS=Mus musculus OX=10090 GN=Hspd1 PE=1 SV=1	Hspd1	4.451	3.02999E-06	Up
Q9CYZ2	Tumor protein D54 OS=Mus musculus OX=10090 GN=Tpd52l2 PE=1 SV=1	Tpd52l2	3.355	3.01661E-06	Up
Q9R0N0	Galactokinase OS=Mus musculus OX=10090 GN=Galk1 PE=1 SV=2	Galk1	3.227	2.87171E-06	Up
P30681	High mobility group protein B2 OS=Mus musculus OX=10090 GN=Hmgb2 PE=1 SV=3	Hmgb2	4.54	2.80996E-06	Up
Q9CWZ3	RNA-binding protein 8A OS=Mus musculus OX=10090 GN=Rbm8a PE=1 SV=4	Rbm8a	3.004	2.69493E-06	Up
Q8R081	Heterogeneous nuclear ribonucleoprotein L OS=Mus musculus OX=10090 GN=Hnrnpl PE=1 SV=2	Hnrnpl	3.843	2.4934E-06	Up
P14824	Annexin A6 OS=Mus musculus OX=10090 GN=Anxa6 PE=1 SV=3	Anxa6	4.163	2.26779E-06	Up
P23927	Alpha-crystallin B chain OS=Mus musculus OX=10090 GN=Cryab PE=1 SV=2	Cryab	9.772	2.23273E-06	Up
P47962	60S ribosomal protein L5 OS=Mus musculus OX=10090 GN=Rpl5 PE=1 SV=3	Rpl5	7.426	2.20986E-06	Up
P62242	40S ribosomal protein S8 OS=Mus musculus OX=10090 GN=Rps8 PE=1 SV=2	Rps8	3.506	2.19644E-06	Up
Q8BKC5	Importin-5 OS=Mus musculus OX=10090 GN=Ipo5 PE=1 SV=3	Ipo5	3.029	2.19445E-06	Up
Q9D8E6	60S ribosomal protein L4 OS=Mus musculus OX=10090 GN=Rpl4 PE=1 SV=3	Rpl4	3.095	2.07472E-06	Up
Q61699	Heat shock protein 105 kDa OS=Mus musculus OX=10090 GN=Hsph1 PE=1 SV=2	Hsph1	4.207	2.02476E-06	Up
Q9DD02	Protein Hikeshi OS=Mus musculus OX=10090 GN=Hikeshi PE=1 SV=1	Hikeshi	8.866	2.02446E-06	Up
Q91ZJ5	UTP--glucose-1-phosphate uridylyltransferase OS=Mus musculus OX=10090 GN=Ugp2 PE=1 SV=3	Ugp2	3.372	1.88972E-06	Up
O54988	STE20-like serine/threonine-protein kinase OS=Mus musculus OX=10090 GN=Slk PE=1 SV=2	Slk	13.097	1.70352E-06	Up
Q6ZWX6	Eukaryotic translation initiation factor 2 subunit 1 OS=Mus musculus OX=10090 GN=Eif2s1 PE=1 SV=3	Eif2s1	3.397	1.52562E-06	Up
Q9CYL5	Golgi-associated plant pathogenesis-related protein 1 OS=Mus musculus OX=10090 GN=Glipr2 PE=1 SV=3	Glipr2	7.829	1.52344E-06	Up
P43276	Histone H1.5 OS=Mus musculus OX=10090 GN=H1-5 PE=1 SV=2	H1-5	3.638	1.22029E-06	Up
Q8BJ71	Nuclear pore complex protein Nup93 OS=Mus musculus OX=10090 GN=Nup93 PE=1 SV=1	Nup93	7.922	1.1944E-06	Up
P62245	40S ribosomal protein S15a OS=Mus musculus OX=10090 GN=Rps15a PE=1 SV=2	Rps15a	3.223	1.09738E-06	Up

P62267	40S ribosomal protein S23 OS=Mus musculus OX=10090 GN=Rps23 PE=1 SV=3	Rps23	10.971	1.06331E-06	Up
O70340	Neuronal pentraxin-2 OS=Mus musculus OX=10090 GN=Nptx2 PE=2 SV=1	Nptx2	14.605	1.03425E-06	Up
Q8R180	ERO1-like protein alpha OS=Mus musculus OX=10090 GN=Ero1a PE=1 SV=2	Ero1a	5.02	9.54211E-07	Up
Q8K183	Pyridoxal kinase OS=Mus musculus OX=10090 GN=Pdxk PE=1 SV=1	Pdxk	4.015	8.5303E-07	Up
Q6ZWV3	60S ribosomal protein L10 OS=Mus musculus OX=10090 GN=Rpl10 PE=1 SV=3	Rpl10	6.559	7.94614E-07	Up
P58044	Isopentenyl-diphosphate Delta-isomerase 1 OS=Mus musculus OX=10090 GN=Idi1 PE=1 SV=1	Idi1	5.052	6.58108E-07	Up
Q6ZWN5	40S ribosomal protein S9 OS=Mus musculus OX=10090 GN=Rps9 PE=1 SV=3	Rps9	3.076	6.22018E-07	Up
O88685	26S proteasome regulatory subunit 6A OS=Mus musculus OX=10090 GN=Psmc3 PE=1 SV=2	Psmc3	3.286	6.06312E-07	Up
Q922J3	CAP-Gly domain-containing linker protein 1 OS=Mus musculus OX=10090 GN=Clip1 PE=1 SV=1	Clip1	9.554	5.81209E-07	Up
Q62433	Protein NDRG1 OS=Mus musculus OX=10090 GN=Ndrg1 PE=1 SV=1	Ndrg1	4.1	5.19615E-07	Up
P47963	60S ribosomal protein L13 OS=Mus musculus OX=10090 GN=Rpl13 PE=1 SV=3	Rpl13	3.861	5.02399E-07	Up
C0HKE6	Histone H2A type 1-I OS=Mus musculus OX=10090 GN=H2ac13 PE=1 SV=1	H2ac13	3.548	4.59881E-07	Up
Q61696	Heat shock 70 kDa protein 1A OS=Mus musculus OX=10090 GN=Hspa1a PE=1 SV=2	Hspa1a	10.082	4.48277E-07	Up
P62852	40S ribosomal protein S25 OS=Mus musculus OX=10090 GN=Rps25 PE=1 SV=1	Rps25	3.026	3.96588E-07	Up
Q9CZX8	40S ribosomal protein S19 OS=Mus musculus OX=10090 GN=Rps19 PE=1 SV=3	Rps19	6.833	2.93947E-07	Up
Q07235	Glia-derived nexin OS=Mus musculus OX=10090 GN=Serpine2 PE=1 SV=2	Serpine2	27.787	2.93841E-07	Up
Q64442	Sorbitol dehydrogenase OS=Mus musculus OX=10090 GN=Sord PE=1 SV=3	Sord	3.846	2.85127E-07	Up
Q9CQ19	Myosin regulatory light polypeptide 9 OS=Mus musculus OX=10090 GN=Myl9 PE=1 SV=3	Myl9	3.284	2.20355E-07	Up
P07901	Heat shock protein HSP 90-alpha OS=Mus musculus OX=10090 GN=Hsp90aa1 PE=1 SV=4	Hsp90aa1	3.073	1.50526E-07	Up
O70475	UDP-glucose 6-dehydrogenase OS=Mus musculus OX=10090 GN=Ugdh PE=1 SV=1	Ugdh	3.801	6.15547E-08	Up
Q8CIN4	Serine/threonine-protein kinase PAK 2 OS=Mus musculus OX=10090 GN=Pak2 PE=1 SV=1	Pak2	4.719	5.83622E-08	Up
Q80X19	Collagen alpha-1(XIV) chain OS=Mus musculus OX=10090 GN=Col14a1 PE=1 SV=2	Col14a1	0.138	5.72346E-10	Down
P54320	Elastin OS=Mus musculus OX=10090 GN=Eln PE=1 SV=2	Eln	0.11	8.44552E-10	Down
Q00493	Carboxypeptidase E OS=Mus musculus OX=10090 GN=Cpe PE=1 SV=2	Cpe	0.226	1.23486E-09	Down
P97300	Neuroplastin OS=Mus musculus OX=10090 GN=Nptn PE=1 SV=3	Nptn	0.231	1.47105E-09	Down
P48759	Pentraxin-related protein PTX3 OS=Mus musculus OX=10090 GN=Ptx3 PE=1 SV=2	Ptx3	0.044	2.06621E-09	Down
Q06890	Clusterin OS=Mus musculus OX=10090 GN=Clu PE=1 SV=1	Clu	0.059	2.4777E-09	Down
Q60847	Collagen alpha-1(XII) chain OS=Mus musculus OX=10090 GN=Col12a1 PE=2 SV=3	Col12a1	0.145	3.56446E-09	Down
Q9JLB4	Cubilin OS=Mus musculus OX=10090 GN=Cubn PE=1 SV=3	Cubn	0.162	8.99824E-09	Down
P55288	Cadherin-11 OS=Mus musculus OX=10090 GN=Cdh11 PE=1 SV=1	Cdh11	0.078	9.1136E-09	Down
P10493	Nidogen-1 OS=Mus musculus OX=10090 GN=Nid1 PE=1 SV=2	Nid1	0.26	1.14904E-08	Down
P10810	Monocyte differentiation antigen CD14 OS=Mus musculus OX=10090 GN=Cd14 PE=1 SV=1	Cd14	0.138	1.28911E-08	Down
P29268	CCN family member 2 OS=Mus musculus OX=10090 GN=Ccn2 PE=1 SV=3	Ccn2	0.132	1.29603E-08	Down

P97946	Vascular endothelial growth factor D OS=Mus musculus OX=10090 GN=Vegfd PE=2 SV=1	Vegfd	0.052	1.42242E-08	Down
P22777	Plasminogen activator inhibitor 1 OS=Mus musculus OX=10090 GN=Serpine1 PE=1 SV=1	Serpine1	0.132	2.79284E-08	Down
Q01149	Collagen alpha-2(I) chain OS=Mus musculus OX=10090 GN=Col1a2 PE=1 SV=2	Col1a2	0.17	2.94725E-08	Down
Q80YX1	Tenascin OS=Mus musculus OX=10090 GN=Tnc PE=1 SV=1	Tnc	0.057	3.00385E-08	Down
P11087	Collagen alpha-1(I) chain OS=Mus musculus OX=10090 GN=Col1a1 PE=1 SV=4	Col1a1	0.199	3.25738E-08	Down
P02468	Laminin subunit gamma-1 OS=Mus musculus OX=10090 GN=Lamc1 PE=1 SV=2	Lamc1	0.333	3.40667E-08	Down
Q05793	Basement membrane-specific heparan sulfate proteoglycan core protein OS=Mus musculus OX=10090 GN=Hspg2 PE=1 SV=1	Hspg2	0.256	3.52488E-08	Down
P35441	Thrombospondin-1 OS=Mus musculus OX=10090 GN=Thbs1 PE=1 SV=1	Thbs1	0.108	4.07928E-08	Down
Q04592	Proprotein convertase subtilisin/kexin type 5 OS=Mus musculus OX=10090 GN=Pcsk5 PE=1 SV=3	Pcsk5	0.162	4.82078E-08	Down
Q6ZWV7	60S ribosomal protein L35 OS=Mus musculus OX=10090 GN=Rpl35 PE=1 SV=1	Rpl35	0.133	6.28876E-08	Down
O35598	Disintegrin and metalloproteinase domain-containing protein 10 OS=Mus musculus OX=10090 GN=Adam10 PE=1 SV=2	Adam10	0.221	6.63544E-08	Down
P08121	Collagen alpha-1(III) chain OS=Mus musculus OX=10090 GN=Col3a1 PE=1 SV=4	Col3a1	0.127	6.69709E-08	Down
Q61245	Collagen alpha-1(XI) chain OS=Mus musculus OX=10090 GN=Col11a1 PE=1 SV=2	Col11a1	0.074	6.86524E-08	Down
P82198	Transforming growth factor-beta-induced protein ig-h3 OS=Mus musculus OX=10090 GN=Tgfbi PE=1 SV=1	Tgfbi	0.121	7.0761E-08	Down
Q3U962	Collagen alpha-2(V) chain OS=Mus musculus OX=10090 GN=Col5a2 PE=1 SV=1	Col5a2	0.116	7.22778E-08	Down
Q6GQT1	Alpha-2-macroglobulin-P OS=Mus musculus OX=10090 GN=A2m PE=2 SV=2	A2m	0.093	7.32092E-08	Down
Q00780	Collagen alpha-1(VIII) chain OS=Mus musculus OX=10090 GN=Col8a1 PE=1 SV=3	Col8a1	0.182	7.73425E-08	Down
Q61554	Fibrillin-1 OS=Mus musculus OX=10090 GN=Fbn1 PE=1 SV=2	Fbn1	0.177	7.7354E-08	Down
P08122	Collagen alpha-2(IV) chain OS=Mus musculus OX=10090 GN=Col4a2 PE=1 SV=4	Col4a2	0.288	7.95095E-08	Down
P97857	A disintegrin and metalloproteinase with thrombospondin motifs 1 OS=Mus musculus OX=10090 GN=Adamts1 PE=1 SV=4	Adamts1	0.124	8.6714E-08	Down
Q9JI75	Ribosyldihydronicotinamide dehydrogenase [quinone] OS=Mus musculus OX=10090 GN=Nqo2 PE=1 SV=3	Nqo2	0.146	9.13066E-08	Down
P11152	Lipoprotein lipase OS=Mus musculus OX=10090 GN=Lpl PE=1 SV=3	Lpl	0.203	9.6982E-08	Down
Q8QZR4	Out at first protein homolog OS=Mus musculus OX=10090 GN=Oaf PE=2 SV=1	Oaf	0.245	1.16123E-07	Down
Q9Z2Q6	Septin-5 OS=Mus musculus OX=10090 GN=Septin5 PE=1 SV=2	Septin5	0.074	1.1624E-07	Down
Q8R2G6	Coiled-coil domain-containing protein 80 OS=Mus musculus OX=10090 GN=Ccdc80 PE=1 SV=2	Ccdc80	0.164	1.22998E-07	Down
Q62059	Versican core protein OS=Mus musculus OX=10090 GN=Vcan PE=1 SV=2	Vcan	0.241	1.52187E-07	Down
Q8R054	Sushi repeat-containing protein SRPX2 OS=Mus musculus OX=10090 GN=Srpx2 PE=1 SV=2	Srpx2	0.078	1.54609E-07	Down
Q01279	Epidermal growth factor receptor OS=Mus musculus OX=10090 GN=Egfr PE=1 SV=1	Egfr	0.202	1.58739E-07	Down
Q62381	Tolloid-like protein 1 OS=Mus musculus OX=10090 GN=Tll1 PE=1 SV=1	Tll1	0.094	1.60039E-07	Down

P97873	Lysyl oxidase homolog 1 OS=Mus musculus OX=10090 GN=Loxl1 PE=2 SV=3	Loxl1	0.142	1.6505E-07	Down
Q9Z175	Lysyl oxidase homolog 3 OS=Mus musculus OX=10090 GN=Loxl3 PE=1 SV=2	Loxl3	0.226	1.66782E-07	Down
Q8C4U3	Secreted frizzled-related protein 1 OS=Mus musculus OX=10090 GN=Sfrp1 PE=1 SV=3	Sfrp1	0.271	1.80065E-07	Down
Q03350	Thrombospondin-2 OS=Mus musculus OX=10090 GN=Thbs2 PE=1 SV=2	Thbs2	0.112	1.85524E-07	Down
O35674	Disintegrin and metalloproteinase domain-containing protein 19 OS=Mus musculus OX=10090 GN=Adam19 PE=2 SV=2	Adam19	0.151	1.87167E-07	Down
P97927	Laminin subunit alpha-4 OS=Mus musculus OX=10090 GN=Lama4 PE=1 SV=2	Lama4	0.255	1.9703E-07	Down
Q811B3	A disintegrin and metalloproteinase with thrombospondin motifs 12 OS=Mus musculus OX=10090 GN=Adamts12 PE=2 SV=2	Adamts12	0.041	1.99916E-07	Down
Q9WV91	Prostaglandin F2 receptor negative regulator OS=Mus musculus OX=10090 GN=Ptgfrn PE=1 SV=2	Ptgfrn	0.177	2.11607E-07	Down
Q8BX17	Gem-associated protein 5 OS=Mus musculus OX=10090 GN=Gemin5 PE=1 SV=2	Gemin5	0.137	2.23701E-07	Down
Q01721	Growth arrest-specific protein 1 OS=Mus musculus OX=10090 GN=Gas1 PE=1 SV=2	Gas1	0.158	2.60176E-07	Down
Q8CG16	Complement C1r-A subcomponent OS=Mus musculus OX=10090 GN=C1ra PE=1 SV=1	C1ra	0.298	2.67017E-07	Down
Q8BUK6	Protein Hook homolog 3 OS=Mus musculus OX=10090 GN=Hook3 PE=1 SV=2	Hook3	0.187	2.88137E-07	Down
P21180	Complement C2 OS=Mus musculus OX=10090 GN=C2 PE=1 SV=2	C2	0.16	2.90331E-07	Down
Q68FF6	ARF GTPase-activating protein GIT1 OS=Mus musculus OX=10090 GN=Git1 PE=1 SV=1	Git1	0.209	2.93443E-07	Down
Q8BPB5	EGF-containing fibulin-like extracellular matrix protein 1 OS=Mus musculus OX=10090 GN=Efemp1 PE=1 SV=1	Efemp1	0.162	2.93935E-07	Down
Q8K2Z2	Pre-mRNA-processing factor 39 OS=Mus musculus OX=10090 GN=Prpf39 PE=1 SV=3	Prpf39	0.1	2.97012E-07	Down
P28654	Decorin OS=Mus musculus OX=10090 GN=Dcn PE=1 SV=1	Dcn	0.221	3.00282E-07	Down
Q61508	Extracellular matrix protein 1 OS=Mus musculus OX=10090 GN=Ecm1 PE=1 SV=2	Ecm1	0.31	3.01744E-07	Down
P11276	Fibronectin OS=Mus musculus OX=10090 GN=Fn1 PE=1 SV=4	Fn1	0.235	3.09442E-07	Down
Q08761	Vitamin K-dependent protein S OS=Mus musculus OX=10090 GN=Pros1 PE=2 SV=1	Pros1	0.154	3.23671E-07	Down
P27090	Transforming growth factor beta-2 proprotein OS=Mus musculus OX=10090 GN=Tgfb2 PE=1 SV=2	Tgfb2	0.228	3.52648E-07	Down
O88207	Collagen alpha-1(V) chain OS=Mus musculus OX=10090 GN=Col5a1 PE=1 SV=2	Col5a1	0.242	3.67136E-07	Down
P97953	Vascular endothelial growth factor C OS=Mus musculus OX=10090 GN=Vegfc PE=1 SV=1	Vegfc	0.223	4.02531E-07	Down
O08712	Tumor necrosis factor receptor superfamily member 11B OS=Mus musculus OX=10090 GN=Tnfrsf11b PE=1 SV=1	Tnfrsf11b	0.186	4.1369E-07	Down
Q9EPL2	Calsyntenin-1 OS=Mus musculus OX=10090 GN=Clstn1 PE=1 SV=1	Clstn1	0.193	4.19956E-07	Down
P47878	Insulin-like growth factor-binding protein 3 OS=Mus musculus OX=10090 GN=Igfbp3 PE=2 SV=2	Igfbp3	0.144	4.65123E-07	Down
P25785	Metalloproteinase inhibitor 2 OS=Mus musculus OX=10090 GN=Timp2 PE=1 SV=2	Timp2	0.288	4.67201E-07	Down
Q6PE55	Platelet-derived growth factor receptor-like protein OS=Mus musculus OX=10090 GN=Pdgfrl PE=2 SV=1	Pdgfrl	0.059	4.72699E-07	Down
D3YXG0	Hemicentin-1 OS=Mus musculus OX=10090 GN=Hmcn1 PE=1 SV=1	Hmcn1	0.03	4.76546E-07	Down

P28301	Protein-lysine 6-oxidase OS=Mus musculus OX=10090 GN=Lox PE=1 SV=1	Lox	0.18	4.9359E-07	Down
P50608	Fibromodulin OS=Mus musculus OX=10090 GN=Fmod PE=2 SV=1	Fmod	0.024	5.13584E-07	Down
P08226	Apolipoprotein E OS=Mus musculus OX=10090 GN=Apoe PE=1 SV=2	Apoe	0.15	5.33888E-07	Down
Q6PB93	Polypeptide N-acetylgalactosaminyltransferase 2 OS=Mus musculus OX=10090 GN=Galnt2 PE=1 SV=1	Galnt2	0.2	5.79303E-07	Down
Q03366	C-C motif chemokine 7 OS=Mus musculus OX=10090 GN=Ccl7 PE=3 SV=1	Ccl7	0.154	6.18053E-07	Down
Q61398	Procollagen C-endopeptidase enhancer 1 OS=Mus musculus OX=10090 GN=Pcolce PE=1 SV=2	Pcolce	0.288	6.2148E-07	Down
Q9R0M3	Sushi-repeat-containing protein SRPX OS=Mus musculus OX=10090 GN=Srpx PE=2 SV=1	Srpx	0.1	6.95815E-07	Down
Q8BLX7	Collagen alpha-1(XVI) chain OS=Mus musculus OX=10090 GN=Col16a1 PE=1 SV=2	Col16a1	0.194	7.12592E-07	Down
Q61292	Laminin subunit beta-2 OS=Mus musculus OX=10090 GN=Lamb2 PE=1 SV=2	Lamb2	0.292	7.60397E-07	Down
Q8K4G1	Latent-transforming growth factor beta-binding protein 4 OS=Mus musculus OX=10090 GN=Ltbp4 PE=1 SV=2	Ltbp4	0.128	7.64789E-07	Down
P27046	Alpha-mannosidase 2 OS=Mus musculus OX=10090 GN=Man2a1 PE=1 SV=2	Man2a1	0.227	7.72882E-07	Down
Q9Z100	Probable carboxypeptidase X1 OS=Mus musculus OX=10090 GN=Cpxm1 PE=2 SV=2	Cpxm1	0.017	7.80935E-07	Down
Q9DC11	Plexin domain-containing protein 2 OS=Mus musculus OX=10090 GN=Plxdc2 PE=1 SV=1	Plxdc2	0.255	7.99729E-07	Down
Q8BV57	Soluble scavenger receptor cysteine-rich domain-containing protein SSC5D OS=Mus musculus OX=10090 GN=Ssc5d PE=1 SV=1	Ssc5d	0.072	8.52236E-07	Down
Q07079	Insulin-like growth factor-binding protein 5 OS=Mus musculus OX=10090 GN=Igfbp5 PE=1 SV=1	Igfbp5	0.258	8.52466E-07	Down
P07214	SPARC OS=Mus musculus OX=10090 GN=Sparc PE=1 SV=1	Sparc	0.277	9.03558E-07	Down
Q8R4K8	Pappalysin-1 OS=Mus musculus OX=10090 GN=Pappa PE=2 SV=2	Pappa	0.023	9.07529E-07	Down
A2ASQ1	Agrin OS=Mus musculus OX=10090 GN=Agrn PE=1 SV=1	Agrn	0.252	9.17371E-07	Down
Q64299	CCN family member 3 OS=Mus musculus OX=10090 GN=Ccn3 PE=1 SV=1	Ccn3	0.192	9.73448E-07	Down
O88968	Transcobalamin-2 OS=Mus musculus OX=10090 GN=Tcn2 PE=1 SV=1	Tcn2	0.101	1.07122E-06	Down
Q9QUN9	Dickkopf-related protein 3 OS=Mus musculus OX=10090 GN=Dkk3 PE=2 SV=1	Dkk3	0.168	1.11307E-06	Down
O89103	Complement component C1q receptor OS=Mus musculus OX=10090 GN=Cd93 PE=1 SV=1	Cd93	0.285	1.31511E-06	Down
Q9WVJ9	EGF-containing fibulin-like extracellular matrix protein 2 OS=Mus musculus OX=10090 GN=Efemp2 PE=1 SV=1	Efemp2	0.216	1.37509E-06	Down
Q921I1	Serotransferrin OS=Mus musculus OX=10090 GN=Tf PE=1 SV=1	Tf	0.082	1.42411E-06	Down
P21956	Lactadherin OS=Mus musculus OX=10090 GN=Mfge8 PE=1 SV=3	Mfge8	0.309	1.43501E-06	Down
P28653	Biglycan OS=Mus musculus OX=10090 GN=Bgn PE=1 SV=1	Bgn	0.209	1.58037E-06	Down
P02469	Laminin subunit beta-1 OS=Mus musculus OX=10090 GN=Lamb1 PE=1 SV=3	Lamb1	0.328	1.61433E-06	Down
O35206	Collagen alpha-1(XV) chain OS=Mus musculus OX=10090 GN=Col15a1 PE=1 SV=2	Col15a1	0.205	1.63329E-06	Down
A2AVA0	Sushi, von Willebrand factor type A, EGF and pentraxin domain-containing protein 1 OS=Mus musculus OX=10090 GN=Svep1 PE=1 SV=1	Svep1	0.052	1.66785E-06	Down

P21460	Cystatin-C OS=Mus musculus OX=10090 GN=Cst3 PE=1 SV=2	Cst3	0.317	1.70401E-06	Down
Q7TQ62	Podocan OS=Mus musculus OX=10090 GN=Podn PE=2 SV=1	Podn	0.201	1.89872E-06	Down
Q9D6X6	Serine protease 23 OS=Mus musculus OX=10090 GN=Prss23 PE=2 SV=2	Prss23	0.274	1.97133E-06	Down
Q9WVB4	Slit homolog 3 protein OS=Mus musculus OX=10090 GN=Slit3 PE=2 SV=2	Slit3	0.087	2.08883E-06	Down
Q8BK62	Olfactomedin-like protein 3 OS=Mus musculus OX=10090 GN=Olfml3 PE=2 SV=2	Olfml3	0.123	2.13664E-06	Down
O08999	Latent-transforming growth factor beta-binding protein 2 OS=Mus musculus OX=10090 GN=Ltbp2 PE=1 SV=2	Ltbp2	0.167	2.1834E-06	Down
Q9EQC7	Follistatin-related protein 3 OS=Mus musculus OX=10090 GN=Fstl3 PE=1 SV=1	Fstl3	0.243	2.23612E-06	Down
O88322	Nidogen-2 OS=Mus musculus OX=10090 GN=Nid2 PE=1 SV=2	Nid2	0.248	2.78299E-06	Down
Q61805	Lipopolysaccharide-binding protein OS=Mus musculus OX=10090 GN=Lbp PE=1 SV=2	Lbp	0.318	2.87136E-06	Down
Q99K41	EMILIN-1 OS=Mus musculus OX=10090 GN=Emilin1 PE=1 SV=1	Emilin1	0.184	3.13075E-06	Down
Q62356	Follistatin-related protein 1 OS=Mus musculus OX=10090 GN=Fstl1 PE=1 SV=2	Fstl1	0.194	3.16671E-06	Down
Q9QZJ6	Microfibrillar-associated protein 5 OS=Mus musculus OX=10090 GN=Mfap5 PE=1 SV=1	Mfap5	0.08	3.30428E-06	Down
Q8CG19	Latent-transforming growth factor beta-binding protein 1 OS=Mus musculus OX=10090 GN=Ltbp1 PE=1 SV=2	Ltbp1	0.063	3.32381E-06	Down
P70428	Exostosin-2 OS=Mus musculus OX=10090 GN=Ext2 PE=1 SV=2	Ext2	0.109	3.35028E-06	Down
Q9DBV4	Matrix remodeling-associated protein 8 OS=Mus musculus OX=10090 GN=Mxra8 PE=1 SV=1	Mxra8	0.091	3.36687E-06	Down
Q3UQ28	Peroxidasin homolog OS=Mus musculus OX=10090 GN=Pxdn PE=1 SV=2	Pxdn	0.257	3.44839E-06	Down
P20722	Bone morphogenetic protein 6 OS=Mus musculus OX=10090 GN=Bmp6 PE=1 SV=2	Bmp6	0.043	3.6522E-06	Down
P51655	Glypican-4 OS=Mus musculus OX=10090 GN=Gpc4 PE=1 SV=2	Gpc4	0.268	4.04339E-06	Down
Q05895	Thrombospondin-3 OS=Mus musculus OX=10090 GN=Thbs3 PE=1 SV=2	Thbs3	0.214	4.11938E-06	Down
Q9JIX8	Apoptotic chromatin condensation inducer in the nucleus OS=Mus musculus OX=10090 GN=Acin1 PE=1 SV=3	Acin1	0.197	4.97165E-06	Down
Q99JR5	Tubulointerstitial nephritis antigen-like OS=Mus musculus OX=10090 GN=Tinagl1 PE=1 SV=1	Tinagl1	0.299	5.07524E-06	Down
P81117	Nucleobindin-2 OS=Mus musculus OX=10090 GN=Nucb2 PE=1 SV=2	Nucb2	0.193	5.22523E-06	Down
Q9QYK5	Heparan-sulfate 6-O-sulfotransferase 1 OS=Mus musculus OX=10090 GN=Hs6st1 PE=1 SV=4	Hs6st1	0.111	5.26007E-06	Down
Q9JM58	Cytokine receptor-like factor 1 OS=Mus musculus OX=10090 GN=Crlf1 PE=1 SV=1	Crlf1	0.267	5.34046E-06	Down
Q9R1B9	Slit homolog 2 protein OS=Mus musculus OX=10090 GN=Slit2 PE=2 SV=2	Slit2	0.089	5.34088E-06	Down
P37889	Fibulin-2 OS=Mus musculus OX=10090 GN=Fbln2 PE=1 SV=2	Fbln2	0.309	5.52242E-06	Down
P08032	Spectrin alpha chain, erythrocytic 1 OS=Mus musculus OX=10090 GN=Spta1 PE=1 SV=3	Spta1	0.259	5.53073E-06	Down
Q8VDV0	Integrin beta-like protein 1 OS=Mus musculus OX=10090 GN=Itgbl1 PE=2 SV=2	Itgbl1	0.082	5.57575E-06	Down
P01029	Complement C4-B OS=Mus musculus OX=10090 GN=C4b PE=1 SV=3	C4b	0.168	5.62579E-06	Down
Q8C9W3	A disintegrin and metalloproteinase with thrombospondin motifs 2 OS=Mus musculus OX=10090 GN=Adamts2 PE=1 SV=2	Adamts2	0.117	5.68648E-06	Down

Q62181	Semaphorin-3C OS=Mus musculus OX=10090 GN=Sema3c PE=1 SV=2	Sema3c	0.049	5.82891E-06	Down
Q61749	Translation initiation factor eIF-2B subunit delta OS=Mus musculus OX=10090 GN=Eif2b4 PE=1 SV=2	Eif2b4	0.085	6.66249E-06	Down
Q60870	Receptor expression-enhancing protein 5 OS=Mus musculus OX=10090 GN=Reep5 PE=1 SV=1	Reep5	0.272	6.84642E-06	Down
Q91VU7	Pseudouridylate synthase 7 homolog OS=Mus musculus OX=10090 GN=Pus7 PE=2 SV=2	Pus7	0.272	7.27223E-06	Down
Q9CQF3	Cleavage and polyadenylation specificity factor subunit 5 OS=Mus musculus OX=10090 GN=Nudt21 PE=1 SV=1	Nudt21	0.203	7.71741E-06	Down
P47880	Insulin-like growth factor-binding protein 6 OS=Mus musculus OX=10090 GN=Igfbp6 PE=2 SV=2	Igfbp6	0.202	7.90065E-06	Down
P97290	Plasma protease C1 inhibitor OS=Mus musculus OX=10090 GN=Serping1 PE=1 SV=3	Serping1	0.206	8.06322E-06	Down
Q9WVH9	Fibulin-5 OS=Mus musculus OX=10090 GN=Fbln5 PE=1 SV=1	Fbln5	0.161	8.66975E-06	Down
Q62000	Mimecan OS=Mus musculus OX=10090 GN=Ogn PE=1 SV=1	Ogn	0.062	9.05469E-06	Down
P04202	Transforming growth factor beta-1 proprotein OS=Mus musculus OX=10090 GN=Tgfb1 PE=1 SV=1	Tgfb1	0.178	9.11052E-06	Down
A2A5R2	Brefeldin A-inhibited guanine nucleotide-exchange protein 2 OS=Mus musculus OX=10090 GN=Arfgef2 PE=1 SV=1	Arfgef2	0.029	9.52133E-06	Down
Q60675	Laminin subunit alpha-2 OS=Mus musculus OX=10090 GN=Lama2 PE=1 SV=2	Lama2	0.054	9.65692E-06	Down
P39053	Dynamin-1 OS=Mus musculus OX=10090 GN=Dnm1 PE=1 SV=2	Dnm1	0.091	9.97592E-06	Down
Q8CJ69	BMP-binding endothelial regulator protein OS=Mus musculus OX=10090 GN=Bmper PE=1 SV=1	Bmper	0.114	1.00774E-05	Down
Q8BLU0	Leucine-rich repeat transmembrane protein FLRT2 OS=Mus musculus OX=10090 GN=Flrt2 PE=1 SV=1	Flrt2	0.235	1.02271E-05	Down
Q3UTJ2	Sorbin and SH3 domain-containing protein 2 OS=Mus musculus OX=10090 GN=Sorbs2 PE=1 SV=2	Sorbs2	0.173	1.05358E-05	Down
Q640N1	Adipocyte enhancer-binding protein 1 OS=Mus musculus OX=10090 GN=Aebp1 PE=1 SV=1	Aebp1	0.182	1.05876E-05	Down
P07141	Macrophage colony-stimulating factor 1 OS=Mus musculus OX=10090 GN=Csf1 PE=1 SV=2	Csf1	0.32	1.06168E-05	Down
Q80V53	Carbohydrate sulfotransferase 14 OS=Mus musculus OX=10090 GN=Chst14 PE=2 SV=2	Chst14	0.156	1.10489E-05	Down
Q00731	Vascular endothelial growth factor A OS=Mus musculus OX=10090 GN=Vegfa PE=1 SV=2	Vegfa	0.268	1.23346E-05	Down
P15116	Cadherin-2 OS=Mus musculus OX=10090 GN=Cdh2 PE=1 SV=2	Cdh2	0.227	1.25902E-05	Down
Q8VCF1	Soluble calcium-activated nucleotidase 1 OS=Mus musculus OX=10090 GN=Cant1 PE=2 SV=1	Cant1	0.22	1.2726E-05	Down
Q8BLI0	ADAMTS-like protein 1 OS=Mus musculus OX=10090 GN=Adamtsl1 PE=2 SV=2	Adamtsl1	0.218	1.31237E-05	Down
P33434	72 kDa type IV collagenase OS=Mus musculus OX=10090 GN=Mmp2 PE=1 SV=1	Mmp2	0.159	1.53115E-05	Down
Q8BLY1	SPARC-related modular calcium-binding protein 1 OS=Mus musculus OX=10090 GN=Smoc1 PE=2 SV=2	Smoc1	0.087	1.55569E-05	Down
P51885	Lumican OS=Mus musculus OX=10090 GN=Lum PE=1 SV=2	Lum	0.131	1.56449E-05	Down
Q8BT60	Copine-3 OS=Mus musculus OX=10090 GN=Cpne3 PE=1 SV=2	Cpne3	0.044	1.59839E-05	Down
Q9JLL0	Cysteine-rich motor neuron 1 protein OS=Mus musculus OX=10090 GN=Crim1 PE=2 SV=2	Crim1	0.084	1.73006E-05	Down
Q66K08	Cartilage intermediate layer protein 1 OS=Mus musculus OX=10090 GN=Cilp PE=1 SV=1	Cilp	0.099	1.82246E-05	Down

Q6ZQ73	Cullin-associated NEDD8-dissociated protein 2 OS=Mus musculus OX=10090 GN=Cand2 PE=1 SV=2	Cand2	0.055	1.85981E-05	Down
P98156	Very low-density lipoprotein receptor OS=Mus musculus OX=10090 GN=Vldlr PE=1 SV=1	Vldlr	0.22	2.03745E-05	Down
Q9CYN9	Renin receptor OS=Mus musculus OX=10090 GN=Atp6ap2 PE=1 SV=2	Atp6ap2	0.074	2.29912E-05	Down
P06802	Ectonucleotide pyrophosphatase/phosphodiesterase family member 1 OS=Mus musculus OX=10090 GN=Enpp1 PE=1 SV=4	Enpp1	0.319	2.35549E-05	Down
Q8VHY0	Chondroitin sulfate proteoglycan 4 OS=Mus musculus OX=10090 GN=Cspg4 PE=1 SV=3	Cspg4	0.296	2.43949E-05	Down
Q99JP6	Homer protein homolog 3 OS=Mus musculus OX=10090 GN=Homer3 PE=1 SV=2	Homer3	0.104	2.50184E-05	Down
P39061	Collagen alpha-1(XVIII) chain OS=Mus musculus OX=10090 GN=Col18a1 PE=1 SV=4	Col18a1	0.261	2.59607E-05	Down
P45700	Mannosyl-oligosaccharide 1,2-alpha-mannosidase IA OS=Mus musculus OX=10090 GN=Man1a1 PE=1 SV=1	Man1a1	0.265	2.79724E-05	Down
P06797	Procathepsin L OS=Mus musculus OX=10090 GN=Ctsl PE=1 SV=2	Ctsl	0.169	2.82359E-05	Down
P97467	Peptidyl-glycine alpha-amidating monooxygenase OS=Mus musculus OX=10090 GN=Pam PE=1 SV=2	Pam	0.101	2.84472E-05	Down
Q9ER58	Testican-2 OS=Mus musculus OX=10090 GN=Spock2 PE=1 SV=1	Spock2	0.265	2.89929E-05	Down
Q8K482	EMILIN-2 OS=Mus musculus OX=10090 GN=Emilin2 PE=1 SV=1	Emilin2	0.082	3.29733E-05	Down
Q9DBD0	Inhibitor of carbonic anhydrase OS=Mus musculus OX=10090 GN=Ica PE=1 SV=1	Ica	0.122	3.37638E-05	Down
O88676	Matrix metalloproteinase-23 OS=Mus musculus OX=10090 GN=Mmp23 PE=2 SV=1	Mmp23	0.099	3.7303E-05	Down
P98063	Bone morphogenetic protein 1 OS=Mus musculus OX=10090 GN=Bmp1 PE=1 SV=2	Bmp1	0.181	3.81403E-05	Down
Q8BWP8	Beta-1,4-glucuronyltransferase 1 OS=Mus musculus OX=10090 GN=B4gat1 PE=1 SV=1	B4gat1	0.306	3.84669E-05	Down
P97464	Exostosin-1 OS=Mus musculus OX=10090 GN=Ext1 PE=1 SV=1	Ext1	0.088	4.62957E-05	Down
P23249	Putative helicase MOV-10 OS=Mus musculus OX=10090 GN=Mov10 PE=1 SV=2	Mov10	0.143	5.05371E-05	Down
P22005	Proenkephalin-A OS=Mus musculus OX=10090 GN=Penk PE=1 SV=2	Penk	0.229	5.08094E-05	Down
Q8K007	Extracellular sulfatase Sulf-1 OS=Mus musculus OX=10090 GN=Sulf1 PE=2 SV=1	Sulf1	0.057	5.27508E-05	Down
P47931	Follistatin OS=Mus musculus OX=10090 GN=Fst PE=2 SV=1	Fst	0.067	5.82187E-05	Down
Q8VCC9	Spondin-1 OS=Mus musculus OX=10090 GN=Spon1 PE=1 SV=1	Spon1	0.084	6.1013E-05	Down
P46414	Cyclin-dependent kinase inhibitor 1B OS=Mus musculus OX=10090 GN=Cdkn1b PE=1 SV=2	Cdkn1b	0.071	6.78051E-05	Down
Q61555	Fibrillin-2 OS=Mus musculus OX=10090 GN=Fbn2 PE=1 SV=2	Fbn2	0.143	7.46739E-05	Down
Q6S5C2	N-acetylglucosamine-1-phosphotransferase subunit gamma OS=Mus musculus OX=10090 GN=Gnptg PE=1 SV=1	Gnptg	0.128	7.80889E-05	Down
Q8R2Z5	von Willebrand factor A domain-containing protein 1 OS=Mus musculus OX=10090 GN=Vwa1 PE=1 SV=1	Vwa1	0.159	8.02063E-05	Down
P25318	Collagen alpha-2(VIII) chain OS=Mus musculus OX=10090 GN=Col8a2 PE=2 SV=2	Col8a2	0.043	8.22323E-05	Down

Q8BJ66	Kazal-type serine protease inhibitor domain-containing protein 1 OS=Mus musculus OX=10090 GN=Kazald1 PE=1 SV=2	Kazald1	0.09	8.56111E-05	Down
P55065	Phospholipid transfer protein OS=Mus musculus OX=10090 GN=Pltp PE=1 SV=1	Pltp	0.112	8.61949E-05	Down
Q99KG5	Lipolysis-stimulated lipoprotein receptor OS=Mus musculus OX=10090 GN=Lsr PE=1 SV=1	Lsr	0.213	8.97591E-05	Down
P04925	Major prion protein OS=Mus musculus OX=10090 GN=Prnp PE=1 SV=2	Prnp	0.164	9.95101E-05	Down
